# Bioinspired Total Synthesis of Erectones A and B, and the Revised Structure of Hyperelodione D

**DOI:** 10.1002/anie.202200420

**Published:** 2022-03-14

**Authors:** Liam J. Franov, Jacob D. Hart, Glenn A. Pullella, Christopher J. Sumby, Jonathan H. George

**Affiliations:** ^1^ Department of Chemistry University of Adelaide Adelaide SA 5000 Australia

**Keywords:** Biomimetic Synthesis, Cascade Reactions, Diels–Alder Reactions, Structure Elucidation, Total Synthesis

## Abstract

The field of biomimetic synthesis seeks to apply biosynthetic hypotheses to the efficient construction of complex natural products. This approach can also guide the revision of incorrectly assigned structures. Herein, we describe the evolution of a concise total synthesis and structural reassignment of hyperelodione D, a tetracyclic meroterpenoid derived from a *Hypericum* plant, alongside some biogenetically related natural products, erectones A and B. The key step in the synthesis of hyperelodione D forms six stereocentres and three rings in a bioinspired cascade reaction that features an intermolecular Diels–Alder reaction, an intramolecular Prins reaction and a terminating cycloetherification.

## Graphical abstract

Chemical speculation on the biosynthesis of hyperelodione D leads to its structural reassignment via a concise total synthesis. The key step is a bioinspired, Diels–Alder cascade reaction between *E*,*E*‐α‐farnesene and a naturally occurring quinone that generates three rings and six stereocentres.

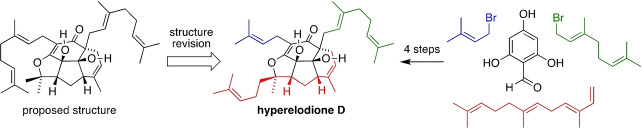

Polycyclic polyprenylated acylphloroglucinols (PPAPs) are a vast family of plant derived meroterpenoid natural products with a broad spectrum of biological activity.^[1]^ Although most PPAPs contain a bicyclo[3.3.1]nonane ring system (e.g. hyperforin),^[2]^ a growing number of “non‐canonical” PPAPs with diverse and often unique polycyclic structures have recently been isolated.^[3]^ For example, in 2021 the structural elucidation of a complex di‐geranylated tetracycle, hyperelodione D (**1**), from *Hypericum elodeoides* Choisy was reported (Figure 1).^[4]^ The proposed structure of hyperelodione D has the same scaffold as two PPAP meroterpenoids previously found in *Hypericum erectum*, erectones A and B (**2** and **3**), which possess either prenyl or geranyl side chains at C6a and C8.^[5]^

**Figure 1:**
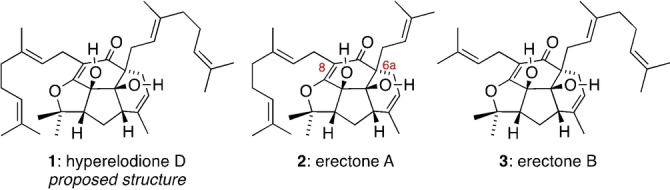
Non‐canonical PPAP natural products of interest in this work.

As suggested in the original isolation work,[^4^, ^5^] the complex tetracyclic structures of **1**, **2** and **3** could arise from a Diels–Alder based cascade reaction between *E*‐β‐ocimene and a suitable dihydroxyquinone dienophile such as **4** (Scheme 1a). Cascade reactions founded on the Diels–Alder cycloaddition are some of the most powerful methods in organic chemistry for the rapid generation of molecular complexity.^[6]^ Furthermore, two of the most spectacular cascade reactions applied in total synthesis employ *E*‐β‐ocimene,^[7]^ a naturally abundant monoterpene which serves as a reactive diene in intermolecular Diels–Alder reactions (Scheme 1b). In a 2011 full paper based on an earlier communication,^[8]^ Majetich and co‐workers showed that hydroxyquinone **5** and *E*‐β‐ocimene underwent a Lewis acid catalysed cascade initiated by an intermolecular Diels–Alder reaction to give perovskone (**6**). In 2013, Liu and co‐workers reported the bioinspired synthesis of bolivianine (**8**) from unsaturated aldehyde **7** and *E*‐β‐ocimene via consecutive intermolecular and intramolecular Diels–Alder reactions.^[9]^ Inspired by these classic total syntheses, we initially targeted a concise synthesis of the proposed structure of hyperelodione D (**1**) using a Diels–Alder cascade reaction between *E*‐β‐ocimene and the relatively simple geranylated dihydroxyquinone **4**. This bioinspired strategy would employ a complex cascade reaction of *E*‐β‐ocimene after just a few steps, thus maximizing its impact.

**Scheme 1:**
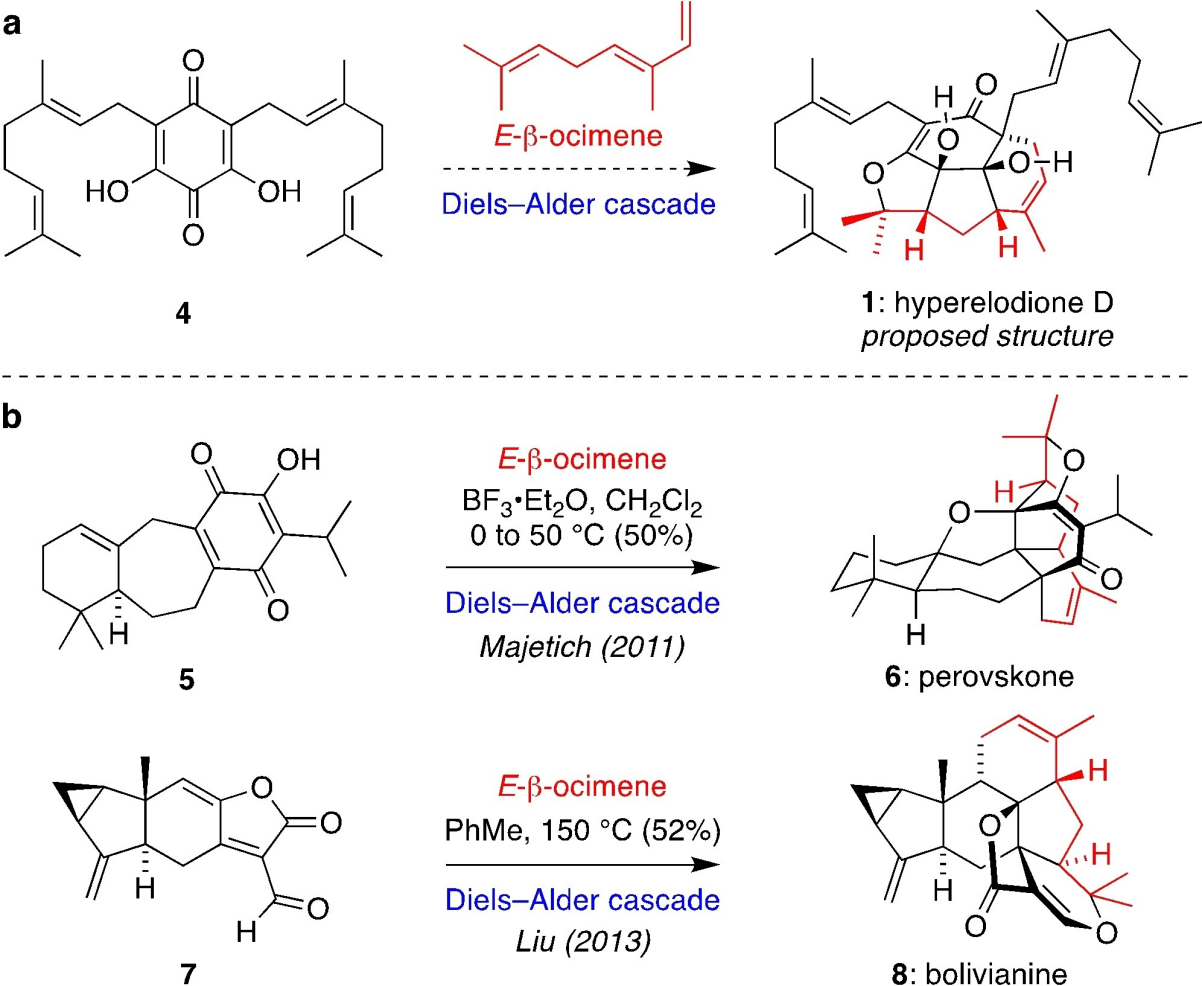
a) Proposed bioinspired synthesis of hyperelodione D. b) Some previous Diels–Alder cascades Involving *E*‐β‐ocimene.

A detailed biosynthetic proposal for hyperelodione D (**1**) is outlined in Scheme 2. As an example of a non‐canonical PPAP, **1** could derive from di‐geranylation of an acylphloroglucinol such as **9**,^[10]^ a common polyketide biosynthetic intermediate in *Hypericum* plants, to give **10** (Scheme 2). Indeed, hyperelodiones E and F (which were co‐isolated with **1**) both contain isobutyryl groups that could also be derived from **9**.^[4]^ A Dakin oxidation of **10** with concomitant loss of isobutyric acid, followed by aerobic oxidation of the resultant hydroquinone, would give the dearomatized dihydroxyquinone **4**.^[11]^ Finally, an intermolecular Diels–Alder reaction between **4** and *E*‐β‐ocimene would form the *endo* adduct **11**,^[12]^ which is primed for an intramolecular Prins reaction^[13]^ and cyclotherification to give **1**.

**Scheme 2:**
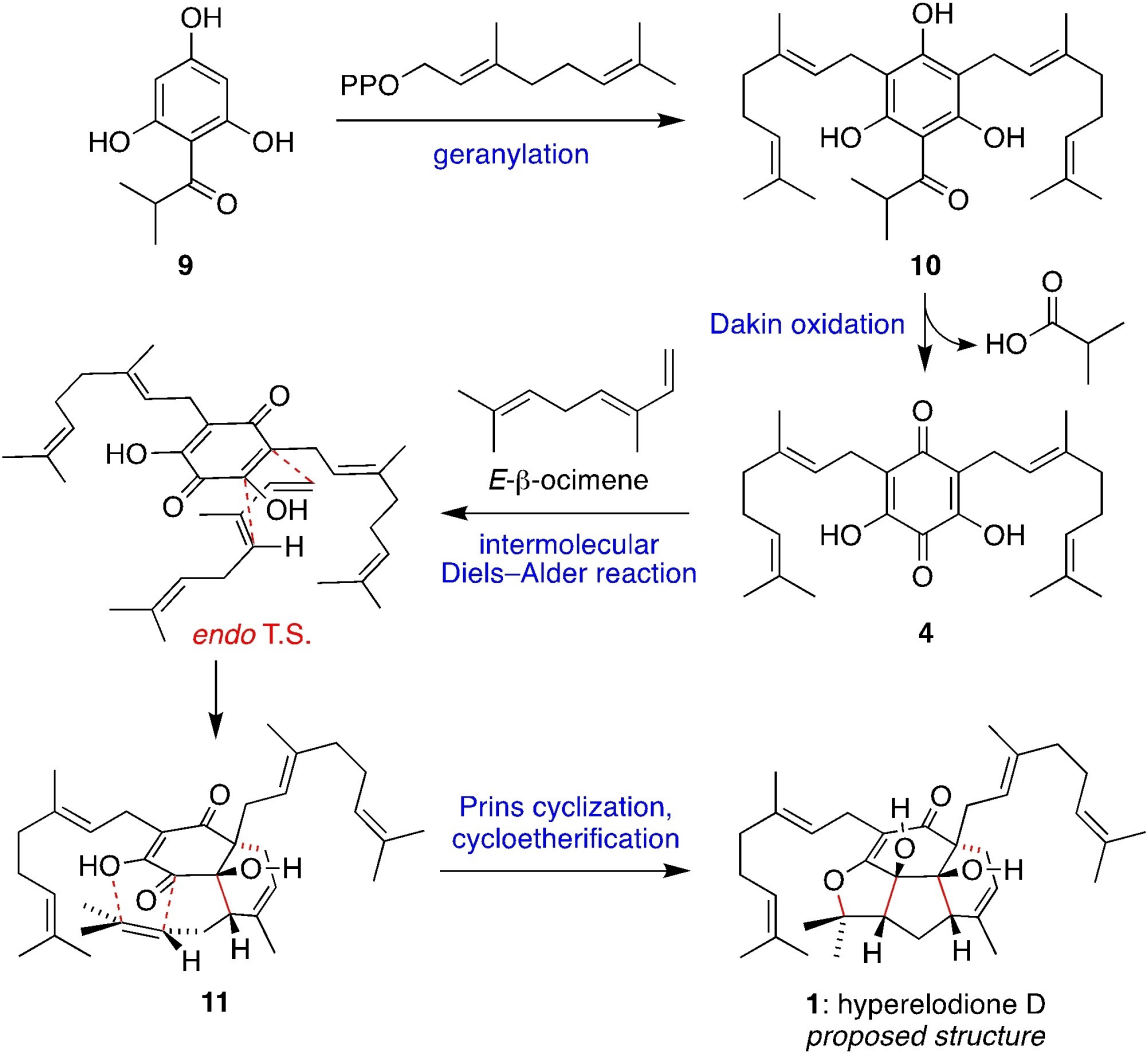
Proposed biosynthesis of hyperelodione D.

The biosynthesis of the proposed structure of hyperelodione D (**1**) served as the blueprint for its concise total synthesis, alongside a di‐prenylated analogue **15** (Scheme 3). Commercially available 2,4,6‐trihydroxybenzaldehyde was di‐prenylated or di‐geranylated under aqueous conditions to give **12** and **13**.^[14]^ Dakin oxidation of aldehydes **12** and **13** under acidic conditions^[15]^ then afforded dihydroxyquinones **14** and **4** in good yield via FeCl_3_‐mediated oxidation of the intermediate hydroquinones. The key Diels–Alder cascades between **14** or **4** and *E*‐β‐ocimene^[16]^ were achieved using Eu(fod)_3_ as a mild Lewis acid catalyst^[17]^ in C_6_H_6_ at 85 °C to give **15** (56 % yield) and **1** (58 % yield). The Diels–Alder cascade reactions could also be achieved less efficiently in 10 % aq. HCl at 50 °C or in PhMe at 150 °C. These highly predisposed cascade reactions generate five stereocentres, four skeletal bonds and three rings in a single step. The initial Diels–Alder reaction of the cascade is both regioselective and diastereoselective, with the less hindered methylene group of *E*‐β‐ocimene attacking the more hindered prenyl/geranyl‐bearing carbon atom of **14** or **4** via an *endo* transition state. Conducting the reaction between quinones **4** or **14** and *E*‐β‐ocimene “on‐water” at neutral pH and at 50 °C gave the *endo* Diels–Alder adducts **11** and **16** as single diastereomers,^[18]^ which were converted into **1** and **15** using catalytic Eu(fod)_3_ in C_6_H_6_. The bowl‐shaped, tetracyclic structure of **15** was confirmed by single crystal X‐ray crystallography.^[19]^ However, at this point we were disappointed to observe that NMR data for synthetic **1** did not quite match the data reported for natural hyperelodione D.

**Scheme 3:**
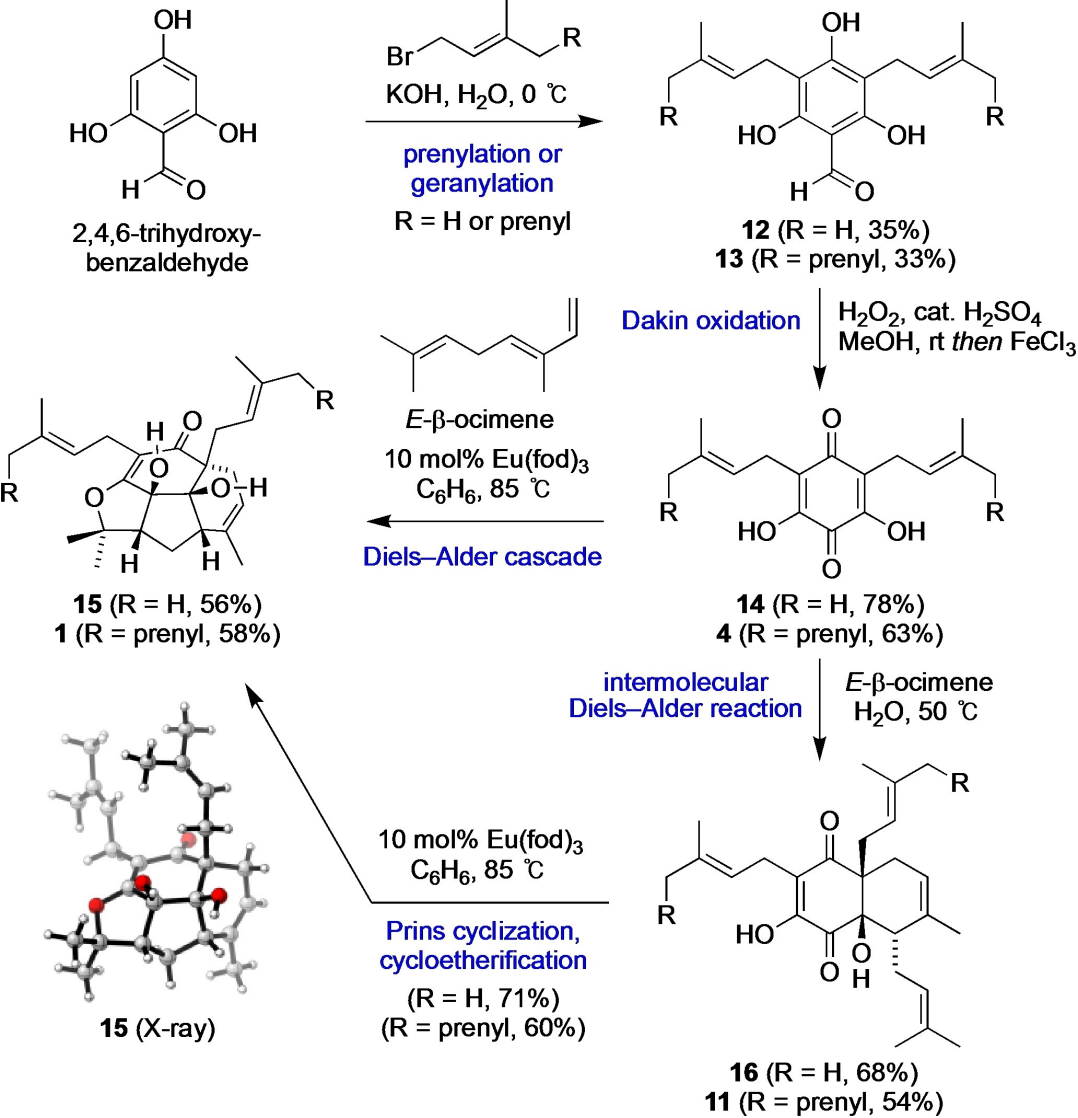
Total synthesis of the proposed structure of hyperelodione D.

Although ^1^H and ^13^C NMR spectra for synthetic **1** are similar to those reported for natural hyperelodione D, there are slight differences in the chemical shifts at C2, C2a, C29 and C30. In addition, the HMBC spectrum of hyperelodione D shows a correlation between C2 and only one methyl substituent. Furthermore, 2D NMR spectra of natural hyperelodione D indicate a prenyl side chain at C8, rather than a geranyl group. We therefore proposed that **17** might be the true structure of hyperelodione D (Scheme 4a). The revised structure **17** is biosynthetically plausible, arising from an intermolecular Diels–Alder reaction between erectquione A (**18**) and *E*,*E‐*α‐farnesene to give **19**, followed by Prins cyclization and cycloetherification. Erectquinone A, which is the proposed biosynthetic precursor of erectones A and B (**2** and **3**), was also isolated from *Hypericum erectum*,^[20]^ and *E*,*E‐*α‐farnesene is found in the essential oils of several *Hypericum* species.^[21]^ Next, we validated *E*,*E,‐*α‐farnesene (which was synthesized as a single stereoisomer according to a known procedure^[22]^) as an effective participant in the Lewis acid catalysed Diels–Alder cascade with quinone **14** to give **20** in 43 % yield (Scheme 4b). NMR spectra of **20** showed a much closer match to the data reported for natural hyperelodione D than our synthetic **1**, which gave confidence in the suggested reassignment to **17**. The relative configuration of the sixth stereocentre formed in the cascade at C2 of **20** was assigned by NOE correlations.

**Scheme 4:**
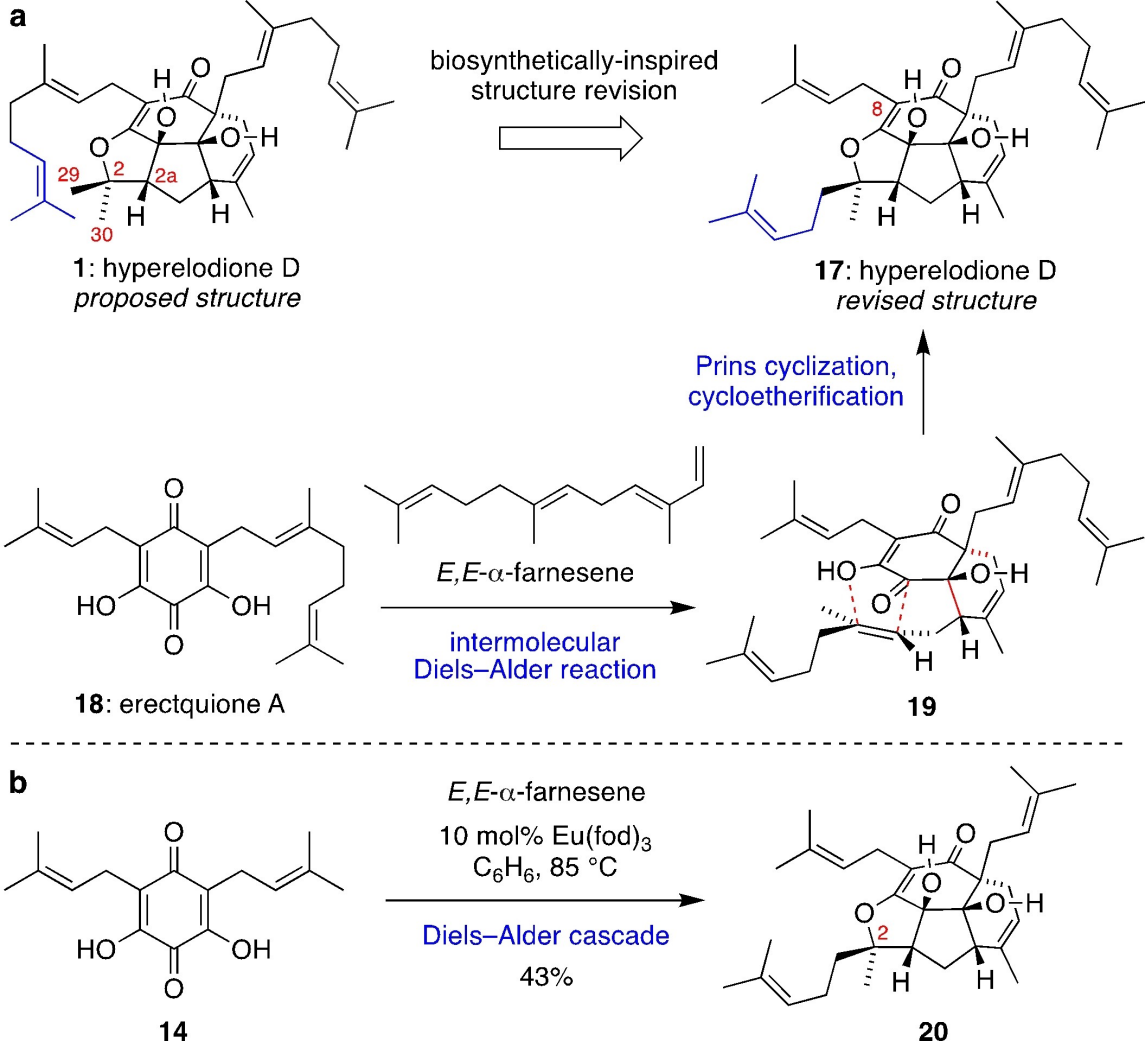
a) Structural reassignment and revised biosynthesis of hyperelodione D. b) Validation of *E*,*E*‐α‐farnesene as a suitable diene in the Diels–Alder cascade.

To confirm the proposed structure revision of hyperelodione D through synthesis, we needed access to erectquione A (**18**) as the quinone dienophile for a bioinspired Diels–Alder cascade with *E*,*E‐*α‐farnesene. In addition, reaction between **18** and *E*‐β‐ocimene should form the constitutionally isomeric erectones A and B (**2** and **3**). Synthesis of **18** was achieved via a stepwise sequence of alkylations that began with a challenging mono‐geranylation of 2,4,6‐trihydroxybenzaldehyde in K_2_CO_3_/acetone to give **21**, in low yield due to competing *O*‐alkylation and di‐alkylations (Scheme 5).^[23]^ Mono‐prenylation of **21** under aqueous conditions then gave **22** in 60 % yield, and finally Dakin oxidation of **22** gave erectquione A (**18**) in 70 % yield.

**Scheme 5:**
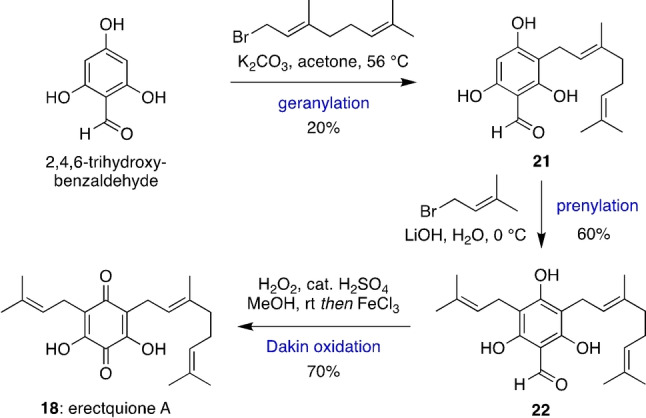
Total synthesis of erectquinone A.

With **18** in hand, we investigated its Diels–Alder reactions with *E*‐β‐ocimene and *E*,*E‐*α‐farnesene (Scheme 6). First, an on‐water catalysed, *endo* Diels–Alder reaction between **18** and *E*‐β‐ocimene gave **23** and **24** in 70 % yield as an inseparable 1 : 1 mixture of regioisomers. We propose that these Diels–Alder adducts could be undiscovered natural products in *Hypericum erectum*. Treating this mixture of **23** and **24** with catalytic Eu(fod)_3_ in C_6_H_6_ at 85 °C then formed a 1 : 1 mixture of erectones A and B (**2** and **3**) in 64 % combined yield via Prins cyclization and cycloetherification. Alternatively, a one‐pot cascade reaction, also catalysed by Eu(fod)_3_, allowed the direct formation of **2** and **3** in 55 % combined yield, which were separable by flash chromatography on silica gel. Similarly, an on‐water, intermolecular Diels–Alder reaction between **18** and *E*,*E‐*α‐farnesene gave regioisomers **19** and **25**, which potentially exist as natural products in *Hypericum elodeoides* Choisy. Treating **19** and **25** with catalytic Eu(fod)_3_ in C_6_H_6_ at 85 °C then gave a separable 1 : 1 mixture of tetracycles **17** and **26**, which could also be formed directly from **18** and *E*,*E‐*α‐farnesene under identical conditions. Pleasingly, NMR data for **17** fully matched that for natural hyperelodione D, thus proving our structural reassignment of this complex meroterpenoid. The prenyl/geranyl substitution pattern of **17** and **26** was established by HMBC correlations and comparison to NMR data for erectones A and B. Alongside the Diels–Alder adducts **19**, **23**, **24** and **25**, it is also probable that tetracycle **26** occurs in nature, and our characterization of these compounds could aid their future discovery in *Hypericum* plants.^[24]^ Finally, the Diels–Alder cascade between erectquione A and *E*‐β‐ocimene was also conducted in 10 % aq. HCl at 50 °C to give erectones A and B in 40 % yield, alongside the Diels–Alder adducts. (The corresponding aqueous Diels–Alder cascade with *E*,*E‐*α‐farnesene gave an inseparable mixture of products.)

**Scheme 6:**
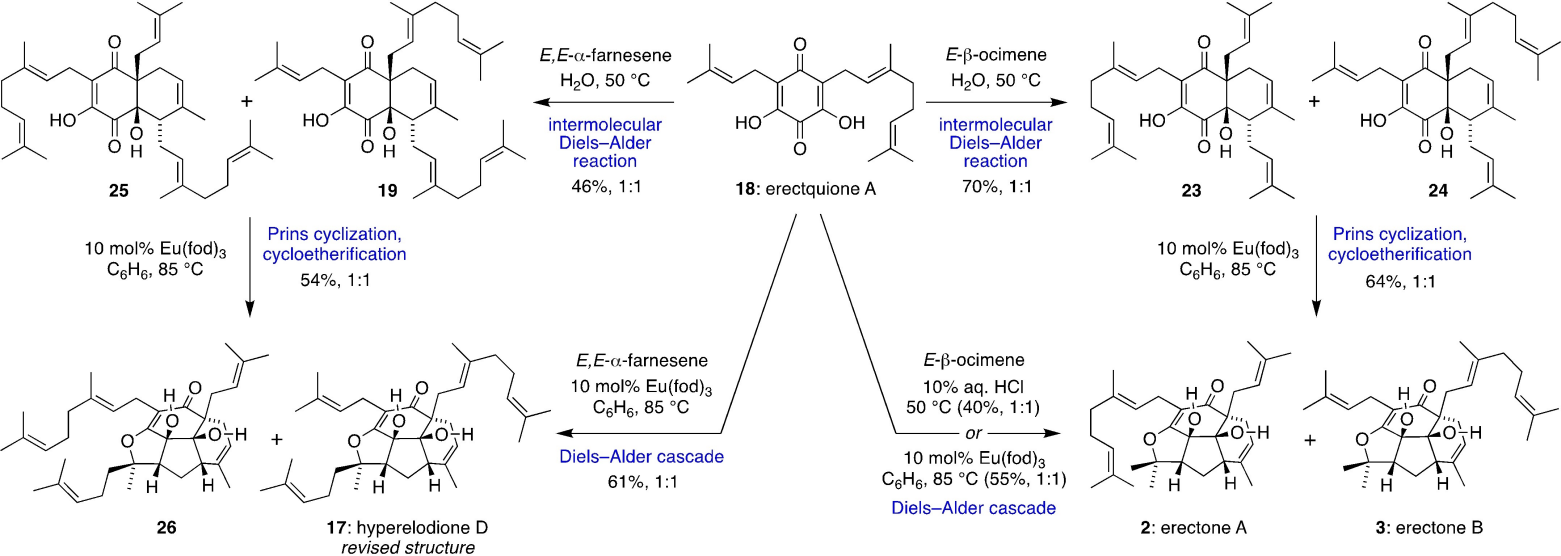
Bioinspired divergent total synthesis of erectones A and B, and the revised structure of hyperelodione D.

In summary, we used biosynthetic speculation to guide the total synthesis and structure revision^[25]^ of hyperelodione D via a series of cascade reactions of gradually increasing complexity. The final Diels–Alder cascade between erectquione A and *E*,*E‐*α‐farnesene to give hyperelodione D constructs six stereocentres and three rings in a single step, thus showcasing the power of biomimetic synthesis. Furthermore, the divergent nature of this strategy was exemplified by the synthesis of erectones A and B and five possible natural products, alongside hyperelodione D, from a common intermediate.

## Conflict of interest

The authors declare no conflict of interest.

## Supporting information

As a service to our authors and readers, this journal provides supporting information supplied by the authors. Such materials are peer reviewed and may be re‐organized for online delivery, but are not copy‐edited or typeset. Technical support issues arising from supporting information (other than missing files) should be addressed to the authors.

Supporting Information

Supporting Information

## Data Availability

The data that support the findings of this study are available in the supplementary material of this article.
