# Supplementary material for: Bioinspired Total Synthesis of Erectones A and B, and the Revised Structure of Hyperelodione D
Source: Angew Chem Int Ed Engl. 2022 Mar 14;61(19):e202200420. doi: 10.1002/anie.202200420 (PMC9314102; doi:10.1002/anie.202200420)
Supplement: Supplementary file 2 — Supporting Information [file ANIE-61-0-s002.pdf]

Angewandte Chemie
Eine Zeitschrift der Gesellschaft Deutscher Chemiker

Supporting Information

Bioinspired Total Synthesis of Erectones A and B, and the Revised Structure of Hyperelodione D

*L. J. Franov, J. D. Hart, G. A. Pullella, C. J. Sumby, J. H. George**

Supporting Information

Table of Contents

1. General Methods 2
2. Experimental Procedures 3
3. NMR Spectra 24
4. Tables of NMR Data 62
5. Single Crystal X-Ray Data 68

1. General Methods

All chemicals were purchased from commercial suppliers and used as received. We used a commercially available 2:1 mixture of *E*- and *Z*-isomers of β-ocimene purchased from Sigma-Aldrich (product number W353901, 100 g = $168 AUD). All reactions were performed under an inert atmosphere of N_2. Room temperature in our laboratory was approximately 20 °C. All organic extracts were dried over anhydrous magnesium sulfate. Dry acetone refers to acetone stored over 4Å molecular sieves for under 24 h. Thin layer chromatography was performed using Merck aluminium sheets coated with silica gel 60 F_{254}. Visualisation was aided by viewing under a UV lamp and staining with *para*-anisaldehyde stain, followed by heating. All R_f values were measured to the nearest 0.05. Flash column chromatography was performed using Carl Roth silica gel 60 (40-62.87 Micron grade). Melting points were recorded on a Stanford Research Systems Digimelt MPA161 digital melting point apparatus and are uncorrected. Infrared spectra were recorded using a Perkin Elmer Spectrum 100 FT-IR spectrometer as the neat compounds. NMR spectra were recorded using either an Agilent 500 MHz DD2 console (^{1}H at 500 MHz, ^{13}C at 125 MHz) or Agilent 600 MHz DD2 console (^{1}H at 600 MHz, ^{13}C at 150 MHz), as specified. Chemical shifts are reported in parts per million (ppm) relative to the tetramethylsilane peak recorded as δ 0.00 ppm in $CDCl_3$/TMS solvent, or the residual acetone (δ 2.05 ppm), chloroform (δ 7.26 ppm), methanol (δ 3.31 ppm) or DMSO (δ 2.50 ppm) peaks. The ^{13}C NMR values were referenced to the residual acetone (δ 29.9 ppm), chloroform (δ 77.16 ppm), methanol (δ 49.00 ppm) or DMSO (δ 39.52 ppm) peaks. ^{13}C NMR values are reported as chemical shift δ. ^{1}H NMR shift values are reported as chemical shift δ, relative integral, multiplicity (s, singlet; d, doublet; t, triplet; q, quartet; m, multiplet) and coupling constant (*J*-values rounded to the nearest 0.1 Hz). Structural assignments were made using COSY, NOESY, HSQC and HMBC experiments. ESI high resolution mass spectra were recorded on an Agilent 6230 TOF LC/MS system.

2. Experimental Procedures

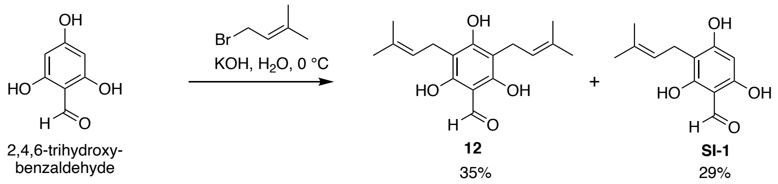

A stirred solution of 2,4,6-trihydroxybenzaldehyde (200 mg, 1.30 mmol) and KOH (182 mg, 3.24 mmol) in water (5 mL) was cooled to 0 °C and treated dropwise with prenyl bromide (0.32 mL, 2.7 mmol). The resulting mixture was stirred for 16 h before being acidified with 1 M HCl (5 mL) and extracted with EtOAc (3 × 10 mL). The organic extracts were washed with brine (20 mL), dried over $MgSO_4$, filtered and concentrated in vacuo. The crude residue was subjected to flash chromatography on SiO_2 (petrol/EtOAc, 9:1 → 4:1 gradient elution) to give aldehyde **12** (127 mg, 34%) as a pale-yellow solid. Further elution afforded aldehyde **SI-1** (84.2 mg, 29%) as an orange oil and recovered 2,4,6-trihydroxybenzaldehyde (26.8 mg, 13%) as an orange solid. Yield of **12** is 39% based on recovered starting material.

Data for 12:
R_f: 0.40 (4:1 petrol/EtOAc).
Mp: 111–114 °C.
IR (neat) ν_{max}: 3375, 2965, 2923, 2855, 1632, 1451, 1375, 1309, 1177, 1097, 746 cm^{-1}.
^{1}H NMR (500 MHz, CD_3OD) δ: 10.06 (s, 1H), 5.12 (t, J = 6.8 Hz, 2H), 3.27 (d, J = 6.8 Hz, 4H), 1.76 (s, 6H), 1.67 (s, 6H) ppm.
^{13}C NMR (125 MHz, CD_3OD) δ: 193.3, 163.8, 160.2, 132.7, 123.7, 108.8, 107.5, 25.9, 22.2, 17.9 ppm.
HRMS (ESI) m/z: Calculated for $C_{17}H_{22}O_4$ $[M+H]^+$ 291.1591; found 291.1589.

Data for SI-1:
R_f: 0.3 (3:2) petrol/EtOAc).
IR (neat) ν_{max}: 3175, 2969, 2914, 1633, 1621, 1520, 1490, 1448, 1372, 1255, 1171, 1128, 1100, 1070, 578 cm^{-1}.
^{1}H NMR (500 MHz, CD_3OD) δ: 10.00 (s, 1H), 5.87 (s, 1H), 5.17 (t, J = 7.2, 7.2 Hz, 1H), 3.16 (d, J = 7.3 Hz, 2H), 1.73 (s, 3H), 1.64 (s, 3H) ppm.
^{13}C NMR (125 MHz, CD_3OD) δ: 192.6, 166.4, 163.6, 162.8, 131.4, 124.1, 107.9, 106.2, 94.5, 25.9, 21.6, 17.8 ppm.
HRMS (ESI) m/z: Calculated for $C_{12}H_{14}O_4$ $[M+H]^+$ 223.0965; found 223.0966.

Larger scale procedure: A stirred solution of 2,4,6-trihydroxybenzaldehyde (6.25 g, 40.6 mmol) and KOH (5.01 g, 89.2 mmol) in water (160 mL) was cooled to 0 °C and treated dropwise with prenyl bromide (10.2 mL, 89.2 mmol). The resulting mixture was stirred at 0 °C for 3 h before being acidified with 1 M HCl (100 mL) and extracted with EtOAc (3 × 100 mL). The organic extracts were washed with brine (100 mL), dried over $MgSO_4$, filtered and concentrated in vacuo. The crude residue was subjected to flash chromatography on SiO_2 (petrol/EtOAc, 19:1 → 4:1 gradient elution) to give aldehyde **12** (3.82 g, 32%) as a pale-yellow solid.

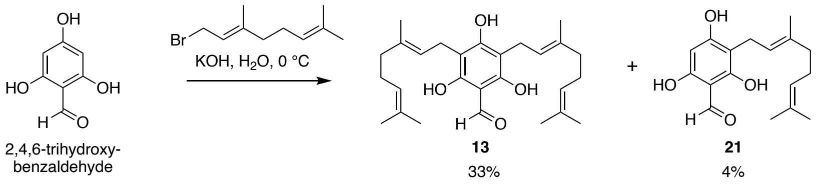

A stirred solution of 2,4,6-trihydroxybenzaldehyde (196 mg, 1.27 mmol) and KOH (178 mg, 3.18 mmol) in water (5 mL) was cooled to 0 °C and treated dropwise with geranyl bromide (0.53 mL, 2.7 mmol). The resulting mixture was stirred for 16 h before being acidified with 1 M HCl (5 mL) and extracted with EtOAc (3 × 10 mL). The organic extracts were washed with brine (20 mL), dried over $MgSO_4$, filtered and concentrated in vacuo. The crude residue was subjected to flash chromatography on SiO_2 (petrol/EtOAc, 9:1 → 4:1 gradient elution) to give aldehyde **13** (177 mg, 33%) as a yellow oil. Further elution afforded the aldehyde **21** (14 mg, 4%) as a red oil and recovered 2,4,6-trihydroxybenzaldehyde (83.7 mg, 43%) as an orange solid. Yield of **13** is 56% based on recovered starting material.

Data for 13:

R_f: 0.45 (4:1 petrol/EtOAc).
IR (neat) ν_{max}: 3376, 2963, 2921, 2854, 1629, 1448, 1375, 1308, 1107, 781cm^{-1}.
^{1}H NMR (500 MHz, CD_3OD) δ: 10.06 (s, 1H), 5.14 (t, J = 6.6 Hz, 2H), 5.05 (t, J = 6.9 Hz, 2H), 3.28 (d, J = 6.8 Hz, 4H), 2.08–2.03 (m, 4H), 1.98–1.94 (m, 4H), 1.76 (s, 6H), 1.61 (s, 6H), 1.55 (s, 6H) ppm.
^{13}C NMR (125 MHz, CD_3OD) δ: 193.3, 163.9, 160.2, 136.4, 132.1, 125.3, 123.7, 108.7, 107.5, 40.8, 27.6, 25.9, 22.2, 17.8, 16.3 ppm.
HRMS (ESI) m/z: Calculated for $C_{27}H_{38}O_4$ $[M+H]^+$ 427.2843; found 427.2854.

Larger scale procedure: A stirred solution of 2,4,6-trihydroxybenzaldehyde (1.00 g, 6.49 mmol), LiOH (342 mg, 14.3 mmol) and NaI (97.3 mg, 0.649 mmol) in water (65 mL) was cooled to 0 °C and treated dropwise with geranyl bromide (2.83 mL, 14.3 mmol). The resulting mixture was stirred at 0 °C for 3 h before being acidified with 1 M HCl (50 mL) and extracted with EtOAc (3 × 50 mL). The organic extracts were washed with brine (150 mL), dried over $MgSO_4$, filtered and concentrated in vacuo. The crude residue was subjected to flash chromatography on SiO_2 (petrol/EtOAc, 9:1) to give aldehyde **13** (891 mg, 30%) as a yellow oil.

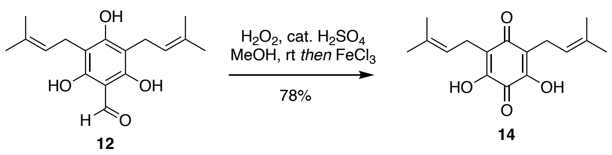

A stirred solution of aldehyde **12** (2.00 g, 6.89 mmol) in MeOH (400 mL) was acidified to pH 2 with conc. H_2SO_4. A solution of 30% aq. H_2O_2 (1.08 mL, 13.8 mmol) in MeOH (90 mL) was added dropwise and the reaction mixture was stirred at rt for 30 min. The solution was then treated with $FeCl_3 \cdot 6H_2O$ (3.72 g, 13.8 mmol) and stirred at rt for a further 5 min. The resulting dark red solution was diluted with water (500 mL) and extracted with EtOAc (3 × 100 mL). The organic extracts were washed with water (100 mL) and brine (200 mL), dried over $MgSO_4$, filtered and concentrated in vacuo. The crude residue was subjected to flash chromatography over SiO_2 (petrol/EtOAc, 9:1 → 4:1 gradient elution) to give quinone **14** (1.48 g, 78%) as a red oil.

Data for 14:

R_f: 0.25 (4:1 petrol/EtOAc)
IR (neat) ν_{max}: 3397, 2968, 2915, 2856, 1653, 1629, 1448, 1329, 1275, 1226, 1173, 1086, 841, 747 cm^{-1}.
^{1}H NMR (500 MHz, $CDCl_3$) δ: 6.46 (s, 2H), 5.12 (t, J = 7.3 Hz, 2H), 3.15 (d, J = 7.3 Hz, 4H), 1.73 (s, 6H), 1.67 (s, 6H) ppm.
^{13}C NMR (125 MHz, $CDCl_3$) δ: 187.0, 180.4, 148.2, 134.0, 122.0, 119.8, 25.9, 22.6, 18.0 ppm.
HRMS (ESI) m/z: Calculated for $C_{16}H_{20}O_4$ $[M+H]^+$ 277.1434; found 277.1441.

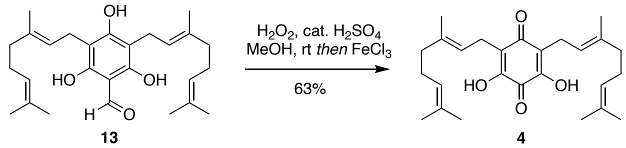

13 → 4

A stirred solution of aldehyde **13** (500 mg, 1.17 mmol) in MeOH (60 mL) was acidified to pH 2 with conc. H_2SO_4. A solution of 30% aq. H_2O_2 (0.18 mL, 1.8 mmol) in MeOH (25 mL) was added dropwise and the reaction solution was stirred at rt for 20 min. The solution was then treated with $FeCl_3 \cdot 6H_2O$ (634 mg, 2.34 mmol) and stirred at rt for a further 5 min. The resulting dark red solution was diluted with water (250 mL) and extracted with EtOAc (3 × 50 mL). The organic extracts were washed with water (100 mL) and brine (100 mL), dried over $MgSO_4$, filtered and concentrated in vacuo. The crude residue was subjected to flash chromatography over SiO_2 (petrol/EtOAc, 9:1 → 4:1 gradient elution) to give quinone **4** (306 mg, 63%) as a red oil.

Data for 4:

R_f: 0.35 (4:1 petrol/EtOAc).
IR (neat) ν_{max}: 3388, 2922, 2584, 1653, 1634, 1456, 1376, 1339, 1094, 985, 744, 699 cm^{-1}.
^{1}H NMR (500 MHz, $CDCl_3$) δ: 6.44 (s, 2H), 5.12 (t, J = 7.2 Hz, 2H), 5.05 (t, J = 6.9 Hz, 2H), 3.16 (d, J = 7.3 Hz, 4H), 2.06–2.02 (m, 4H), 1.97–1.94 (m, 4H), 1.73 (s, 6H), 1.65 (s, 6H), 1.57 (s, 6H) ppm.
^{13}C NMR (125 MHz, $CDCl_3$) δ: 186.9, 180.4, 148.2, 137.6, 131.6, 124.3, 122.0, 119.6, 39.9, 26.7, 25.8, 22.6, 17.8, 16.3 ppm.
HRMS (ESI) m/z: Calculated for $C_{26}H_{36}O_4$ $[M+H]^+$ 413.2686; found 413.2686.

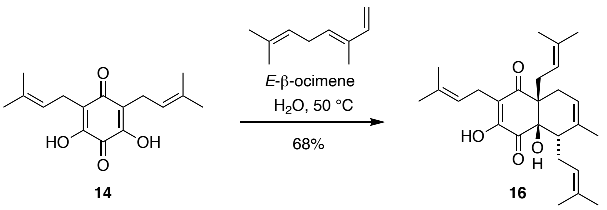

14 16

A mixture of quinone **14** (90.9 mg, 0.329 mmol) and *E*-β-ocimene (224 mg, 1.65 mmol) in water (1.3 mL) was stirred at 50 °C for 16 h, then cooled to rt. The solution was diluted with water (10 mL) and extracted with EtOAc (10 mL). The extract was washed with brine (10 mL), dried over $MgSO_4$, filtered and concentrated in vacuo. The crude residue was subjected to flash chromatography over SiO_2 (petrol/EtOAc, 9:1) to give the *endo* adduct **16** as a pale-yellow oil (92.9 mg, 68%).

Data for 16:

R_f: 0.55 (1:1 petrol/EtOAc).
IR (neat) ν_{max}: 3406, 2971 2359, 2332, 1717, 1616, 1440, 1375, 1332, 1261, 1224, 1171, 1121, 1101, 1065, 1018, 958, 848, 806 cm^{-1}.
^1H NMR (500 MHz, $CDCl_3$) δ: 6.56 (br d, *J* = 3.6 Hz, 1H), 5.49 (br s, 1H), 5.04 (t, *J* = 7.3 Hz, 1H), 4.69 (t, *J* = 7.2 Hz, 1H), 4.54 (br s, 1H), 3.48 (s, 1H), 3.23 (dd, *J* = 13.3, 7.9 Hz, 1H), 3.14–3.07 (m, 2H), 2.60 (dd, *J* = 14.5, 8.4 Hz, 1H), 2.17 (d, *J* = 10.6 Hz, 1H), 2.10 (d, *J* = 17.1 Hz, 1H), 2.01 (dd, *J* = 14.5, 6.3 Hz, 1H), 1.80 (d, *J* = 18.8 Hz, 1H), 1.71 (s, 6H), 1.67 (m, 1H), 1.64 (s, 3H), 1.62 (s, 3H), 1.60 (s, 3H), 1.48 (s, 3H), 1.40 (s, 3H) ppm.
^{13}C NMR (125 MHz, $CDCl_3$) δ: 199.9, 197.9, 153.3, 135.5, 135.0, 134.3, 131.9, 126.8, 123.3, 121.0, 118.8, 118.3, 80.8, 56.8, 55.5, 39.3, 30.2, 28.1, 26.1, 25.9, 25.8, 23.6, 22.8, 18.3, 18.0 ppm.
HRMS (ESI) *m/z*: Calculated for $C_{26}H_{36}O_4Na$ $[M+Na]^+$ 435.2506; found 435.2509.

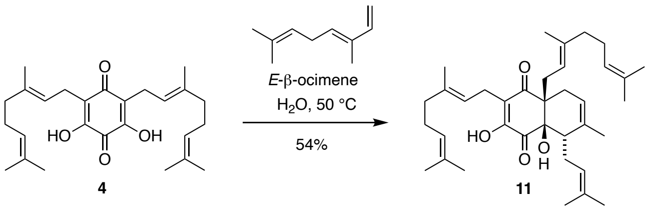

A mixture of quinone **4** (170 mg, 0.412 mmol) and *E*-β-ocimene (281 mg, 2.06 mmol) in water (1.7 mL) was stirred at 50 °C for 16 h, then cooled to rt. The solution was diluted with water (20 mL) and extracted with EtOAc (20 mL). The extract was washed with brine (20 mL), dried over $MgSO_4$, filtered and concentrated in vacuo. The crude residue was subjected to flash chromatography over SiO_2 (petrol/EtOAc, 9:1) to give the *endo* adduct **11** (122 mg, 54%) as a yellow oil.

Data for 11:

R_f: 0.30 (4:1 petrol/EtOAc).
IR (neat) ν_{max}: 3377, 2966, 2914, 2856, 1663, 1440, 1363, 1346, 1321, 1169, 1065, 1028, 837, 803, 742 cm^{-1}.
^{1}H NMR (500 MHz, $CDCl_3$) δ: 6.56 (s, 1H), 5.49 (br s, 1H), 5.09–5.02 (m, 3H), 4.70 (t, *J* = 7.1 Hz, 1H), 4.55 (br s, 1H), 3.48 (s, 1H), 3.26 (dd, *J* = 13.3, 7.7 Hz, 1H), 3.13–3.08 (m, 2H), 2.63 (dd, *J* = 14.3, 9.0 Hz, 1H), 2.17 (d, *J* = 10.3 Hz, 1H), 2.10 (d, *J* = 16.2 Hz, 1H), 2.04–1.99 (m, 5H), 1.94–1.90 (m, 4H), 1.78 (d, *J* = 18.7 Hz), 1.72 (s, 6H), 1.69 (s, 3H), 1.67 (m, 1H), 1.65 (s, 3H), 1.60 (s, 3H), 1.58 (s, 3H), 1.56 (s, 3H), 1.48 (s, 3H), 1.41 (s, 3H) ppm.
^{13}C NMR (125 MHz, $CDCl_3$) δ: 199.8, 197.8, 153.4, 138.8, 138.1, 135.5, 131.9, 131.6, 131.5, 126.8, 124.3, 124.2, 123.3, 121.1, 118.5, 118.4, 80.8, 56.9, 55.5, 40.1, 40.0, 38.9, 30.2, 28.0, 26.8, 26.6, 25.84, 25.78, 23.5, 22.9, 18.3, 17.81, 17.77, 16.4, 16.3 ppm.
HRMS (ESI) *m/z*: Calculated for $C_{36}H_{52}O_4$ $[M+H]^+$ 549.3938; found 549.3950.

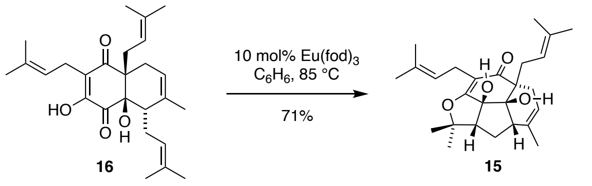

A solution of *endo* adduct **16** (70.0 mg, 0.170 mmol) and Eu(fod)$_3$ (17.6 mg, 0.0170 mmol) in benzene (1.7 mL) was stirred at 85 °C for 16 h, then cooled to rt. The resulting solution was diluted with water (10 mL) and extracted with EtOAc (2 × 15 mL). The organic extracts were washed with brine (25 mL), dried over $MgSO_4$, filtered and concentrated in vacuo. The crude residue was subjected to flash chromatography over SiO_2 (petrol/EtOAc, 9:1 → 4:1 gradient elution) to give tetracycle **15** (49.8 mg, 71%) as a pale-yellow solid.

Data for 15:

R$_f$: 0.25 (4:1 petrol/EtOAc).
Mp: 135–139 °C.
IR (neat) ν_{max}: 3397, 2924, 2955, 1718, 1617, 1448, 1375, 1262, 1224, 1171, 1121, 1101, 1065, 1018, 958, 849, 809, 700 cm^{-1}.
^{1}H NMR (500 MHz, $CDCl_3$) δ: 5.40 (d, *J* = 5.9 Hz, 1H), 5.14–5.10 (m, 1H), 4.99 (t, *J* = 7.1 Hz, 1H), 3.44 (s, 1H), 3.23 (s, 1H), 2.96–2.87 (m, 2H), 2.65–2.58 (m, 3H), 2.48 (dd, *J* = 11.2, 7.5 Hz, 1H), 2.30 (dd, *J* = 14.7, 4.6 Hz, 1H), 2.06–1.99 (m, 2H), 1.70 (s, 3H), 1.68 (s, 3H), 1.67 (s, 6H), 1.63 (s, 3H), 1.52 (s, 3H), 1.37 (s, 3H), 1.15–1.07 (m, 1H) ppm.
^{13}C NMR (125 MHz, $CDCl_3$) δ: 199.0, 170.6, 136.7, 134.4, 131.8, 122.4, 121.7, 121.5, 117.9, 90.3, 84.8, 79.2, 52.4, 52.1, 51.7, 35.3, 31.2, 29.4, 27.3, 26.1, 25.8, 25.1, 22.0, 20.0, 18.3, 18.0 ppm.
HRMS (ESI) *m/z*: Calculated for $C_{26}H_{36}O_4$ $[M+Na]^+$ 435.2506; found 435.2518.

Alternative method heating in aqueous HCl: A mixture of *endo* adduct **16** (94.1 mg, 0.228 mmol) in 10% aq. HCl (5 mL) was stirred at 50 °C for 16 h, cooled to rt and extracted with EtOAc (25 mL). The extract was washed with brine (25 mL), dried over $MgSO_4$, filtered and concentrated in vacuo. The crude residue was subjected to flash chromatography over SiO_2 (petrol/EtOAc, 9:1 → 4:1 gradient elution) to give tetracycle **15** (42.1 mg, 45%) as a pale-yellow solid. Data for **15** matched that obtained previously.

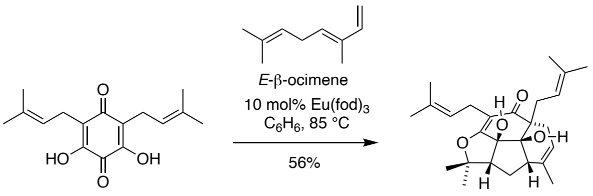

14 15

A solution of quinone **14** (85.9 mg, 0.311 mmol), *E*-β-ocimene (212 mg, 1.55 mmol) and Eu(fod)$_3$ (32.3 mg, 0.0311 mmol) in benzene (3.0 mL) was stirred at 85 °C for 16 h, then cooled to rt. The resulting solution was diluted with water (10 mL) and extracted with EtOAc (2 × 15 mL). The organic extracts were washed with brine (25 mL), dried over $MgSO_4$, filtered and concentrated in vacuo. The crude residue was subjected to flash chromatography over SiO_2 (petrol/EtOAc, 9:1 → 4:1 gradient elution) to give tetracycle **15** (71.7 mg, 56%) as a pale-yellow solid. Data for **15** matched that obtained previously.

Alternative method heating in aqueous HCl: A mixture of quinone **14** (38.1 mg, 0.138 mmol) and *E*-β-ocimene (93.7 mg, 0.688 mmol) in 10% aq. HCl (5 mL) was stirred at 50 °C for 16 h, cooled to rt and extracted with EtOAc (25 mL). The organic extract was washed with brine (25 mL), dried over $MgSO_4$, filtered and concentrated in vacuo. The crude residue was subjected to flash chromatography over SiO_2 (petrol/EtOAc, 9:1 → 4:1 gradient elution) to give tetracycle **15** (26.6 mg, 47%) as a pale-yellow solid. Data for **15** matched that obtained previously.

Alternative method heating in PhMe: A solution of quinone **14** (50.0 mg, 0.181 mmol) and *E*-β-ocimene (123 mg, 0.905 mmol) in PhMe (1.0 mL) was stirred at 150 °C for 16 h, then cooled to rt and concentrated in vacuo. The crude residue was subjected to flash chromatography over SiO_2 (petrol/EtOAc, 9:1 → 4:1 gradient elution) to give tetracycle **15** (32.1 mg, 43%) as a pale-yellow solid. Data for **15** matched that obtained previously.

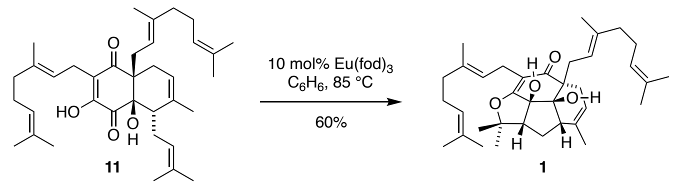

A solution of **11** (54.0 mg, 0.0984 mmol) and Eu(fod)$_3$ (10.2 mg, 0.00983 mmol) in benzene (1.0 mL) was stirred at 85 °C for 16 h, then cooled to rt. The resulting solution was diluted with water (10 mL) and extracted with EtOAc (2 × 15 mL). The orgnic extracts were washed with brine (25 mL), dried over $MgSO_4$, filtered and concentrated in vacuo. The crude residue was subjected to flash chromatography over SiO_2 (petrol/EtOAc, 9:1 → 7:3 gradient elution) to give tetracycle **1** (32.2 mg, 60%) as a colourless oil.

Data for 1:

R$_f$: 0.40 (4:1 petrol/EtOAc).
IR (neat) *ν$_{max}$*: 3420, 2965, 2924, 2855, 1773, 1624, 1445, 1369, 1262, 1201, 1058, 960, 851, 776 cm^{-1}.
^{1}H NMR (600 MHz, CDCl$_3$) *δ*: 5.42–5.40 (m, 1H), 5.15 (m, 1H), 5.09–5.05 (m, 1H), 5.04–4.99 (m, 2H), 3.44 (s, 1H), 3.23 (s, 1H), 2.98–2.89 (m, 2H), 2.66–2.61 (m, 3H), 2.48 (dd, *J* = 11.2, 7.6 Hz, 1H), 2.31 (dd, *J* = 14.5, 5.0 Hz, 1H), 2.06–1.98 (m, 8H), 1.92–1.89 (m, 2H), 1.68 (s, 9H), 1.66 (s, 6H), 1.58 (s, 6H), 1.52 (s, 3H), 1.37 (s, 3H), 1.15–1.08 (m, 1H) ppm.
^{13}C NMR (150 MHz, CDCl$_3$) *δ*: 199.1, 170.7, 140.5, 135.6, 134.5, 132.2, 131.4, 124.6, 123.8, 122.3, 121.4, 121.3, 117.9, 90.3, 84.7, 79.2, 52.4, 52.1, 51.7, 40.0, 39.9, 35.4, 31.4, 29.5, 27.3, 27.1, 26.5, 25.85, 25.83, 25.1, 22.0, 20.1, 17.81, 17.78, 16.7, 16.3 ppm.
HRMS (ESI) *m/z*: Calculated for $C_{36}H_{52}O_4Na$ [M+Na]$^+$ 571.3758; found 571.3771.

Alternative method heating in aqueous HCl: A mixture of **11** (56.0 mg, 0.102 mmol) in 10% aq. HCl (5 mL) was stirred at 50 °C for 33 h, cooled to rt and extracted with EtOAc (25 mL). The organic extract was washed with brine (25 mL), dried over $MgSO_4$, filtered and concentrated in vacuo. The crude residue was subjected to flash chromatography over SiO_2 (petrol/EtOAc, 9:1 → 7:3 gradient elution) to give tetracycle **1** (21.2 mg, 38%) as a colourless oil. Data for **1** matched that obtained previously.

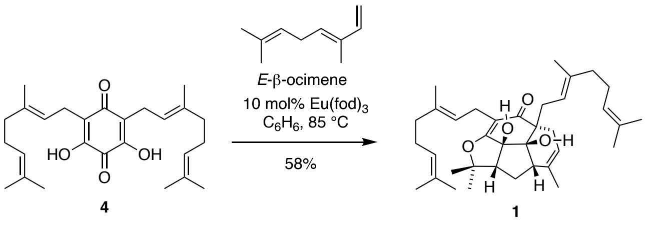

A solution of quinone **4** (94.9 mg, 0.230 mmol), *E*-β-ocimene (156 mg, 1.15 mmol) and $Eu(fod)_3$ (23.9 mg, 0.0230 mmol) in benzene (2.3 mL) was stirred at 85 °C for 16 h, then cooled to rt. The resulting solution was diluted with water (10 mL) and extracted with EtOAc (2 × 15 mL). The organic extracts were washed with brine (25 mL), dried over $MgSO_4$, filtered and concentrated in vacuo. The crude residue was subjected to flash chromatography over SiO_2 (petrol/EtOAc, 9:1 → 4:1 gradient elution) to give tetracycle **1** (72.8 mg, 58%) as a colourless oil. Data for **1** matched that obtained previously.

Alternative method heating in aqueous HCl: A mixture of quinone **4** (84.2 mg, 0.204 mmol) and *E*-β-ocimene (139 mg, 1.02 mmol) in 10% aq. HCl (5 mL) was stirred at 50 °C for 33 h, cooled to rt and extracted with EtOAc (25 mL). The extract was washed with brine (25 mL), dried over $MgSO_4$, filtered and concentrated in vacuo. The crude residue was subjected to flash chromatography over SiO_2 (petrol/EtOAc, 9:1 → 3:2 gradient elution) to give tetracycle **1** (34.1 mg, 31%) as a colourless oil. Data for **1** matched that obtained previously.

Alternative method heating in PhMe: A solution of quinone **4** (23.2 mg, 0.0560 mmol) and *E*-β-ocimene (38.1 mg, 0.280 mmol) in PhMe (1.0 mL) was stirred at 150 °C for 16 h, then cooled to rt and concentrated in vacuo. The crude residue was subjected to flash chromatography over SiO_2 (petrol/EtOAc, 9:1 → 4:1 gradient elution) to give tetracycle **1** (8.1 mg, 26%) as a colourless oil. Data for **1** matched that obtained previously.

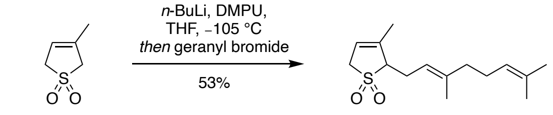

A solution of 3-methylsulfolene[1] (917 mg, 6.94 mmol) and DMPU (1.90 mL, 16 mmol) in dry THF (30 mL) under N_2 was cooled to –105 °C (ethanol/liquid N_2). To this solution was added 2.0 M *n*-BuLi in hexane (3.50 mL, 7.00 mmol) dropwise over 20 min, maintaining an internal temperature below –90 °C. The solution was stirred for a further 10 min before addition of geranyl bromide (1.40 mL, 7.03 mmol) in a single portion. The solution was then warmed slowly to rt and stirred for 2 h. The resulting solution was concentrated in vacuo, redissolved in Et_2O (50 mL), stirred at rt for 50 min, filtered through celite and concentrated in vacuo. The crude residue was subjected to flash chromatography over SiO_2 (petrol/EtOAc, 20:1 → 4:1 gradient elution) to give sulfolene **SI-2** (980 mg, 53%) as a colourless oil.

Partial data for sulfolene SI-2:
R_f: 0.40 (4:1 petrol/EtOAc).
^{1}H NMR (500 MHz, $CDCl_3$) *δ*: 5.67 (s, 1H), 5.22 (t, *J* = 7.2 Hz, 1H), 5.09–5.04 (m, 1H), 3.72–3.60 (m, 2H), 3.51 (t, *J* = 6.6 Hz, 1H), 2.64–2.50 (m, 2H), 2.10–2.01 (m, 4H), 1.85 (s, 3H), 1.67 (s, 3H), 1.66 (s, 3H), 1.60 (s, 3H) ppm.
^{13}C NMR (125 MHz, $CDCl_3$) *δ*: 139.2, 139.0, 131.8, 124.1, 118.4, 117.3, 67.5, 55.8, 39.9, 26.7, 26.6, 25.8, 18.5, 17.8, 16.4 ppm.

Data for sulfolene **SI-2** matched those in literature.[2]

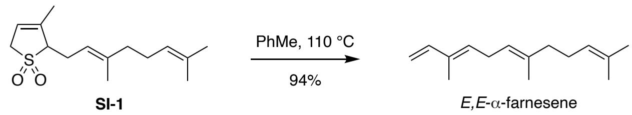

A solution of sulfolene **SI-2** (721 mg, 2.68 mmol) in dry, degassed PhMe (25 mL) was stirred under reflux under N_2 for 2.5 h, cooled to rt and concentrated in vacuo. The crude residue was subjected to flash chromatography over SiO_2 (neat petrol) to give *E*,*E*-α-farnesene (517 mg, 94%) as a colourless oil.

Partial data for (*E,E*)-α-farnesene:
R_f: 0.50 (petrol).
^{1}H NMR (500 MHz, $CDCl_3$) *δ*: 6.37 (dd, *J* = 17.4, 10.7 Hz, 1H), 5.46 (t, *J* = 7.4 Hz, 1H), 5.14–5.07 (m, 3H), 4.93 (d, *J* = 10.7 Hz, 1H), 2.84 (t, *J* = 7.2 Hz, 2H), 2.10–2.05 (m, 2H), 2.01–1.97 (m, 2H), 1.73 (s, 3H), 1.68 (s, 3H), 1.64 (s, 3H), 1.60 (s, 3H) ppm.
^{13}C NMR (125 MHz, $CDCl_3$) *δ*: 141.7, 135.9, 133.9, 132.0, 131.6, 124.4, 122.2, 110.6, 39.8, 27.4, 26.8, 25.8, 17.8, 16.3, 11.8 ppm.

Data for (*E,E*)-α-farnesene matched those in literature.[2]

---

[1] H. T. Dang, V. T. Nguyen, V. D. Nguyen, H. D. Arman, O. V. Larionov, *Org. Biomol. Chem.* **2018**, *16*, 3605.
[2] S. Fielder, D. D. Rowan, P. F. Reay, *J. Label. Compd. Radiopharm.* **1993**, *33*, 965.

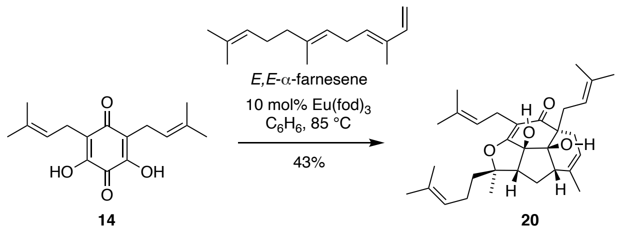

A solution of quinone **14** (79.8 mg, 0.289 mmol), *E,E*-α-farnesene (148 mg, 0.722 mmol) and Eu(fod)$_3$ (30.0 mg, 0.0289 mmol) in benzene (2.9 mL) was stirred at 85 °C for 16 h, then cooled to rt. The resulting solution was diluted with water (10 mL) and extracted with EtOAc (2 × 15 mL). The organic extracts were washed with brine (25 mL), dried over $MgSO_4$, filtered and concentrated in vacuo. The crude residue was subjected to flash chromatography over SiO_2 (petrol/EtOAc, 19:1 → 9:1 gradient elution) to give tetracycle **20** (59.9 mg, 43%) as an orange oil.

Data for 20:

R$_f$: 0.40 (4:1 petrol/EtOAc).
IR (neat) ν_{max}: 3401, 2965, 2913, 2856, 2363, 2343, 1667, 1614, 1448, 1440, 1376, 1339, 1295, 1255, 1224, 1189, 1157, 1102, 1082, 1060, 1020, 960, 913, 854, 809, 732 cm^{-1}.
^{1}H NMR (500 MHz, CDCl$_3$) δ: 5.41–5.39 (m, 1H), 5.16 (t, *J* = 6.7 Hz, 1H), 5.13–5.09 (m, 1H), 5.02 (t, *J* = 7.4 Hz, 1H), 3.39 (s, 1H), 3.26 (s, 1H), 2.97–2.87 (m, 2H), 2.65–2.58 (m, 3H), 2.56 (dd, *J* = 11.2, 7.4 Hz, 1H), 2.29 (dd, *J* = 14.5, 5.4 Hz, 1H), 2.19–1.97 (m, 6H), 1.70 (s, 6H), 1.67 (s, 3H), 1.66 (s, 3H), 1.63 (s, 6H), 1.52 (s, 3H), 1.34 (s, 3H), 1.14–1.07 (m, 1H) ppm.
^{13}C NMR (125 MHz, CDCl$_3$) δ: 198.9, 170.3, 136.6, 134.3, 132.1, 131.7, 124.1, 122.4, 121.8, 121.5, 117.9, 92.7, 84.6, 79.2, 52.3, 51.7, 50.4, 39.3, 35.2, 31.0, 29.4, 26.0, 25.80, 25.78, 23.7, 22.0, 21.9, 20.0, 18.3, 17.9, 17.8 ppm.
HRMS (ESI) *m/z*: Calculated for $C_{31}H_{45}O_4$ [M+H]$^+$ 481.3312; found 481.3311.

Alternative method heating in aqueous HCl: A mixture of quinone **14** (69.9 mg, 0.253 mmol) and *E,E*-α-farnesene (131 mg, 0.633 mmol) in 10% aq. HCl (5 mL) was stirred at 50 °C for 3.5 d, cooled to rt and extracted with EtOAc (25 mL). The extract was washed with brine (25 mL), dried over $MgSO_4$, filtered and concentrated in vacuo. The crude residue was subjected to flash chromatography over SiO_2 (petrol/EtOAc, 9:1) to give tetracycle **20** (26.0 mg, 22%) as an orange oil. Data for **20** matched that obtained previously.

Alternative method heating in PhMe: A solution of quinone **14** (100 mg, 0.362 mmol) and *E,E*-α-farnesene (185 mg, 0.905 mmol) in PhMe (3.0 mL) was stirred at 150 °C for 16 h, then cooled to rt and concentrated in vacuo. The crude residue was subjected to flash chromatography over SiO_2 (petrol/EtOAc, 9:1 → 4:1 gradient elution) to give tetracycle **20** (43.4 mg, 25%) as a colourless oil. Data for **20** matched that obtained previously.

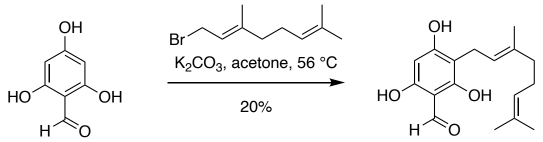

Geranyl bromide (10.6 g, 46.2 mmol) was added to a stirred mixture of 2,4,6-trihydroxybenzaldehyde (7.12 g, 46.2 mmol) and K_2CO_3 (6.38 g, 46.2 mmol) in dry acetone (230 mL). The resulting mixture was stirred at reflux under N_2 for 16 h, cooled to rt and concentrated in vacuo. The remaining residue was treated with 10% aq. HCl (100 mL), and the resulting mixture extracted with EtOAc (3 × 50 mL). The combined extracts were washed with brine (150 mL), dried over $MgSO_4$, filtered and concentrated in vacuo. The red, crude residue was partially dissolved in CH_2Cl_2 (100 mL) and cooled to 0 °C, and the resulting pale-orange precipitate was removed by vacuum filtration. The filtrate was concentrated, and the resulting red oil was subjected to flash chromatography on SiO_2 (petrol/EtOAc, 9:1 → 3:2 gradient elution) to give aldehyde **21** (2.70 g, 20%) as an orange gum.

Data for 21:

R_f: 0.45 (1:1 petrol/EtOAc).
IR (neat) ν_{max}: 3347, 3190, 2981, 2917, 1637, 1609, 1523, 1448, 1375, 1309, 1624, 1168, 1124, 1072, 968, 894, 817, 742, 669, 580 cm^{-1}.
^{1}H NMR (500 MHz, CD_3OD) δ: 10.00 (s, 1H), 5.88 (s, 1H)*, 5.19–5.15 (m, 1H), 5.05 (t, J = 7.1 Hz, 1H), 3.17 (d, J = 7.1 Hz, 2H), 2.07–2.02 (m, 2H), 1.96–1.92 (m, 2H), 1.73 (s, 3H), 1.61 (s, 3H), 1.55 (s, 3H) ppm.
*Integral attenuated due to hydrogen-deuterium exchange with deuterons from CD_3OD.
^{13}C NMR (125 MHz, CD_3OD) δ: 192.5, 166.4, 163.6, 162.8, 135.0, 132.0, 125.5, 124.2, 107.9, 106.3, 94.5 (br), 40.9, 27.7, 25.8, 21.6, 17.7, 16.2. ppm.
HRMS (ESI) m/z: Calculated for $C_{17}H_{23}O_4$ $[M+H]^+$ 291.1591; found 291.1603.

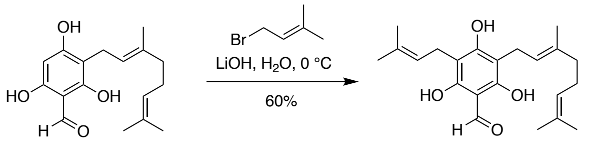

A stirred solution of aldehyde **21** (471 mg, 1.62 mmol) and LiOH (66.1 mg, 2.76 mmol) in water (55 mL) was cooled to 0 °C and treated dropwise with prenyl bromide (0.26 mL, 2.3 mmol). The resulting mixture was stirred at 0 °C for 40 min before being acidified with 10% aq. HCl (20 mL) and extracted with EtOAc (3 × 20 mL). The organic extracts were washed with brine (50 mL), dried over $MgSO_4$, filtered and concentrated in vacuo. The red, crude residue was subjected to flash chromatography on SiO_2 (petrol/EtOAc, 9:1) to give aldehyde **22** (347 mg, 60%) as a red oil.

Data for 22:

R$_f$: 0.45 (4:1 petrol/EtOAc).
IR (neat) *v$_{max}$*: 3367, 2968, 2913, 2955, 1629, 1613, 1440, 1366, 1308, 1098, 770 cm^{-1}.
^{1}H NMR (600 MHz, CD_3OD) *δ*: 10.06 (s, 1H), 5.15–5.11 (m, 2H), 5.04 (t, *J* = 7.1 Hz, 1H), 3.27 (m, 4H), 2.08–2.03 (m, 2H), 1.99–1.95 (m, 2H), 1.76 (s, 6H), 1.66 (s, 3H), 1.61 (s, 3H), 1.55 (s, 3H) ppm.
^{13}C NMR (150 MHz, CD_3OD) *δ*: 193.3, 163.9, 160.2 (br), 136.4, 132.6, 132.1, 125.3, 123.7, 108.74, 108.67, 107.5, 40.8, 27.6, 26.0, 25.9, 22.3, 22.2, 18.0, 17.7, 16.3 ppm.
HRMS (ESI) *m/z*: Calculated for $C_{22}H_{30}O_4$ $[M+H]^+$ 359.2217; found 359.2221.

Alternative method:

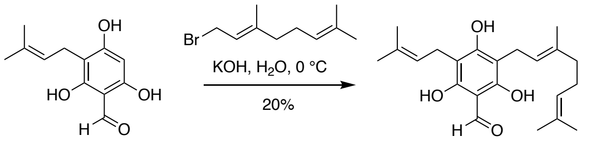

A stirred solution of aldehyde **SI-1** (25 mg, 0.11 mmol) and KOH (7.9 mg, 0.14 mmol) in water (2 mL) was cooled to 0 °C and treated dropwise with geranyl bromide (0.025 mL, 0.12 mmol). The resulting mixture was stirred for 16 h before being acidified with 1 M HCl (5 mL) and extracted with EtOAc (3 × 10 mL). The organic extracts were washed with brine (20 mL), dried over $MgSO_4$, filtered and concentrated in vacuo. The crude residue was subjected to flash chromatography on SiO_2 (petrol/EtOAc, 19:1 → 9:1 gradient elution) to give aldehyde **22** (8.2 mg, 20%) as a red oil.

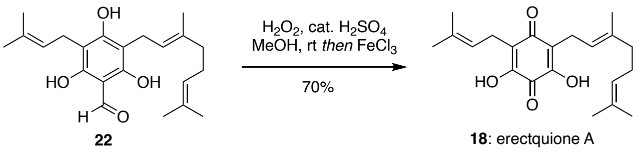

A stirred solution of aldehyde **22** (430 mg, 1.20 mmol) in MeOH (70 mL) was acidified to pH 2 with conc. H_2SO_4. A solution of 30% aq. H_2O_2 (0.19 mL, 2.40 mmol) in MeOH (16 mL) was added dropwise and the reaction solution was stirred at rt for 30 min. The solution was then treated with $FeCl_3{\cdot}6H_2O$ (648 mg, 2.40 mmol) and stirred at rt for a further 5 min. The resulting brown solution was diluted with water (300 mL) and extracted with EtOAc (3 × 100 mL). The extract was washed with water (100 mL) and brine (100 mL), dried over $MgSO_4$, filtered and concentrated in vacuo. The brown, crude residue was subjected to flash chromatography over SiO_2 (petrol/EtOAc, 9:1 → 4:1 gradient elution) to give erectquione A (**18**) (289 mg, 70%) as a red oil.

Data for 18:

R_f: 0.35 (4:1 petrol/EtOAc).
IR (neat) ν_{max}: 3401, 2966, 2919, 2855, 1653, 1633, 1448, 1376, 1332, 1274, 1225, 1172, 1090, 840, 750 cm^{-1}.
^{1}H NMR (500 MHz, $CDCl_3$) δ: 6.42 (s, 2H), 5.12 (t, J = 7.1 Hz, 2H), 5.05 (t, J = 6.6 Hz, 1H), 3.16 (m, 4H), 2.07–2.02 (m, 2H), 1.98–1.94 (m, 2H), 1.73 (s, 6H), 1.67 (s, 3H), 1.65 (s, 3H), 1.57 (s, 3H) ppm.
^{13}C NMR (125 MHz, $CDCl_3$) δ: 186.9, 180.4, 148.19, 148.16, 137.6, 134.0, 131.6, 124.3, 122.04, 121.96, 119.8, 119.6, 39.9, 26.7, 25.9, 25.8, 22.64, 22.57, 18.0, 17.8, 16.3 ppm.
HRMS (ESI) *m/z*: Calculated for $C_{21}H_{28}O_4$ $[M+H]^+$ 345.2060; found 345.2057.

Data for **18** match those reported in the literature for natural erectquione A.

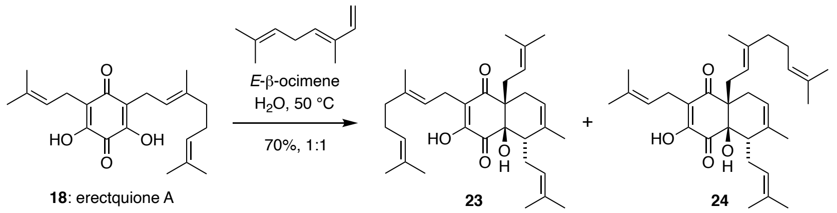

A mixture of erectquione A (**18**) (77.7 mg, 0.226 mmol) and *E*-β-ocimene (154 mg, 1.13 mmol) in water (1 mL) was stirred at 50 °C for 16 h, then cooled to rt. The solution was diluted with water (10 mL) and extracted with EtOAc (10 mL). The organic extract was washed with brine (10 mL), dried over $MgSO_4$, filtered and concentrated in vacuo. The crude residue was subjected to flash chromatography over SiO_2 (petrol/EtOAc, 9:1) to give a 1:1 mixture of *endo* adducts **23** and **24** (76.2 mg, 70%) as a yellow oil.

Data for 23 and 24:

R_f: 0.40 (4:1 petrol/EtOAc).
IR (neat) ν_{max}: 3417, 2968, 2929, 2856, 1668, 1448, 1379, 1361, 1346, 1172, 1072, 1041, 670 cm^{-1}.
^{1}H NMR (500 MHz, $CDCl_3$) δ: 6.56–6.54 (m, 2H), 5.49 (br s, 2H), 5.09–5.02 (m, 4H), 4.69 (m, 2H), 4.55 (br s, 2H), 3.48 (s, 2H), 3.29–3.21 (m, 2H), 3.13–3.08 (m, 4H), 2.65–2.58 (m, 2H), 2.17 (d, *J* = 10.5 Hz, 2H), 2.10 (d, *J* = 18.1 Hz, 2H), 2.04–1.99 (m, 6H), 1.94–1.89 (m, 4H), 1.79 (d, *J* = 19.2 Hz, 2H), 1.71 (s, 12H), 1.69 (s, 3H), 1.67 (m, 2H), 1.65 (s, 3H), 1.63 (s, 3H), 1.60 (s, 9H), 1.59 (s, 3H), 1.56 (s, 3H), 1.48 (s, 6H), 1.40 (s, 6H) ppm.
^{13}C NMR (125 MHz, $CDCl_3$) δ: 199.9, 199.8, 197.9, 197.8, 153.4, 153.3, 138.7, 138.1, 135.6, 135.5, 135.1, 134.4, 131.9, 131.6, 131.6, 126.8, 126.8, 124.3, 124.3, 124.2, 124.2, 123.4, 123.3, 121.1, 121.1, 121.0, 118.8, 118.7, 118.4, 118.4, 118.3, 118.3, 80.8, 80.8, 56.9, 56.8, 55.6, 55.5, 40.1, 40.0, 39.1, 39.0, 30.2, 28.0, 26.8, 26.7, 26.1, 25.9, 25.9, 25.8, 23.6, 23.5, 22.8, 18.3, 18.0, 17.8, 17.8, 16.4, 16.3 ppm.
HRMS (ESI) *m/z*: Calculated for $C_{31}H_{45}O_4$ $[M+H]^+$ 481.3312; found 481.3313.

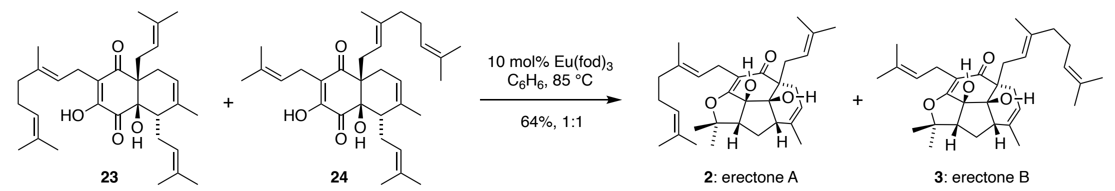

A solution of *endo* adducts **23** and **24** (125 mg, 0.260 mmol) and Eu(fod)$_3$ (26.9 mg, 0.0260 mmol) in benzene (2.6 mL) was stirred at 85 °C for 16 h, then cooled to rt. The resulting solution was diluted with water (10 mL) and extracted with EtOAc (2 × 15 mL). The organic extracts were washed with brine (25 mL), dried over $MgSO_4$, filtered and concentrated in vacuo. The crude residue was subjected to flash chromatography over SiO_2 (petrol/EtOAc, 9:1 → 4:1 gradient elution) to give a 1:1 mixture of erectones A and B (**2** and **3**) (79.8 mg, 64%) as a clear oil. Repeated chromatography afforded analytically pure samples of erectones A and B.

Data for 2:
R$_f$: 0.35 (4:1 petrol/EtOAc)
IR (neat) *ν_{max}*: 3406, 2969, 2926, 1727, 1617, 1440, 1374, 1263, 1224, 1187, 1172, 1121, 960, 908, 850, 809, 730, 646 cm^{-1}.
^{1}H NMR (600 MHz, $CDCl_3$) *δ*: 5.42–5.38 (m, 1H), 5.14–5.10 (m, 1H), 5.07 (t, *J* = 6.9 Hz, 1H), 5.00 (t, *J* = 6.9 Hz, 1H), 3.43 (s, 1H), 3.21 (s, 1H), 2.97–2.89 (m, 2H), 2.66–2.58 (m, 3H), 2.48 (dd, *J* = 11.3, 7.4 Hz, 1H), 2.29 (dd, *J* = 14.9, 5.5 Hz, 1H), 2.06–1.98 (m, 4H), 1.93–1.89 (m, 2H), 1.70 (s, 3H), 1.68 (s, 9H), 1.66 (s, 3H), 1.57 (s, 3H), 1.51 (s, 3H), 1.36 (s, 3H), 1.14–1.07 (m, 1H) ppm.
^{13}C NMR (150 MHz, $CDCl_3$) *δ*: 199.0, 170.7, 136.9, 135.6, 134.4, 131.4, 124.6, 122.4, 121.4, 121.3, 117.9, 90.3, 84.7, 79.2, 52.4, 52.1, 51.7, 39.9, 35.4, 31.2, 29.5, 27.3, 27.1, 26.1, 25.8, 25.1, 22.0, 20.1, 18.3, 17.8, 16.3 ppm.
HRMS (ESI) *m/z*: Calculated for $C_{31}H_{45}O_4$ [M+H]$^+$ 481.3312; found 481.3305.

Data for 3:
R$_f$: 0.35 (4:1 petrol/EtOAc).
IR (neat) *ν_{max}*: 3401, 2971, 2910, 2856, 1617, 1540, 1445, 1439, 1378, 1345, 1256, 1225, 1189, 1082, 1021, 958, 857, 809, 669 cm^{-1}.
^{1}H NMR (500 MHz, $CDCl_3$) *δ*: 5.41–5.39 (m, 1H), 5.14 (dd, *J* = 9.0, 6.0 Hz, 1H), 5.05–5.02 (m, 1H), 4.99 (dd, *J* = 7.8, 6.8 Hz, 1H), 3.47 (s, 1H), 3.26 (s, 1H), 2.97–2.87 (m, 2H), 2.66–2.60 (m, 3H), 2.47 (dd, *J* = 11.2, 7.5 Hz, 1H), 2.30 (dd, *J* = 14.6, 5.0 Hz, 1H), 2.06–1.98 (m, 6H), 1.67 (s, 12H), 1.61 (s, 3H), 1.57 (s, 3H), 1.51 (s, 3H), 1.36 (s, 3H), 1.14–1.06 (m, 1H) ppm.
^{13}C NMR (125 MHz, $CDCl_3$) *δ*: 199.1, 170.7, 140.3, 134.5, 132.2, 131.8, 123.8, 122.2, 121.7, 121.4, 117.9, 90.3, 84.7, 79.1, 52.4, 52.1, 51.7, 40.0, 35.2, 31.3, 29.4, 27.3, 26.5, 25.8, 25.7, 25.1, 22.0, 20.0, 18.0, 17.8, 16.7 ppm.
HRMS (ESI) *m/z*: Calculated for $C_{31}H_{44}O_4Na$ [M+Na]$^+$ 503.3132; found 503.3138.

Alternative method heating in aqueous HCl: A mixture of *endo* adducts **23** and **24** (97.3 mg, 0.202 mmol) in 10% aq. HCl (5 mL) was stirred at 50 °C for 1.5 d, cooled to rt and extracted with EtOAc (10 mL). The extract was washed with brine (10 mL), dried over $MgSO_4$, filtered and concentrated in vacuo. The crude residue was subjected to flash chromatography over SiO_2 (petrol/EtOAc, 9:1 → 4:1 gradient elution) to give *endo* adducts **23** and **24** (59.5 mg, 61%). Further elution afforded erectones A and B (1:1 mixture of **2** and **3**) (21.4 mg, 22%) as a clear oil. Data for **2** and **3** matched that obtained previously.

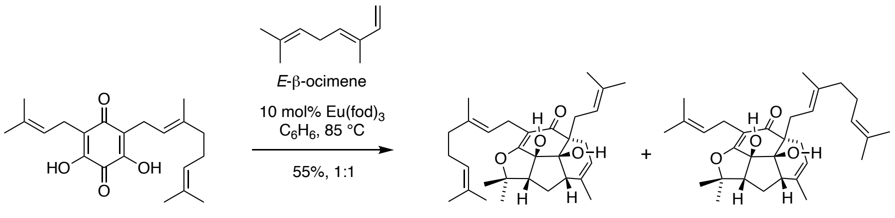

A solution of erectquione A (**18**) (56.3 mg, 0.163 mmol), *E*-β-ocimene (111 mg, 0.817 mmol) and $Eu(fod)_3$ (17.0 mg, 0.0163 mmol) in benzene (1.6 mL) was stirred at 85 °C for 16 h, then cooled to rt. The resulting solution was diluted with water (10 mL) and extracted with EtOAc (2 × 15 mL). The organic extracts were washed with brine (25 mL), dried over $MgSO_4$, filtered and concentrated in vacuo. The crude residue was subjected to flash chromatography over SiO_2 (petrol/EtOAc, 1:0 → 4:1 gradient elution) to give a 1:1 mixture of erectones A and B (**2** and **3**) (43.5 mg, 55%) as a clear oil. Data for **2** and **3** matched that obtained previously.

Alternative method heating in aqueous HCl: A mixture of erectquione A (**18**) (47.2 mg, 0.137 mmol) and *E*-β-ocimene (93.3 mg, 0.685 mmol) in 10% aq. HCl (5 mL) was stirred at 50 °C for 36 h, cooled to rt and extracted with EtOAc (10 mL). The organic extract was washed with brine (10 mL), dried over $MgSO_4$, filtered and concentrated in vacuo. The crude residue was subjected to flash chromatography over SiO_2 (petrol/EtOAc, 1:0 → 4:1 gradient elution) to give a 1:1 mixture of *endo* adducts **23** and **24** (20.3 mg, 31%) as a yellow oil. Further elution afforded a 1:1 mixture of erectones A and B (**2** and **3**) (26.2 mg, 40%) as a clear oil. Data for **2** and **3** matched that obtained previously.

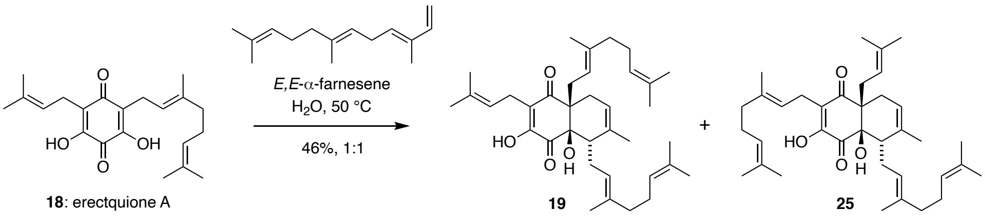

18: erectquione A **19** **25**

A mixture of erectquione A (**18**) (122 mg, 0.354 mmol) and *E,E*-α-farnesene (180 mg, 0.884 mmol) in water (1.5 mL) was stirred at 50 °C for 16 h, cooled to rt and extracted with EtOAc (10 mL). The organic extract was washed with brine (10 mL), dried over $MgSO_4$, filtered and concentrated in vacuo. The crude residue was subjected to flash chromatography over SiO_2 (petrol/EtOAc, 4:1) to give a 1:1 mixture of *endo* adducts **19** and **25** (86.6 mg, 46%) as a yellow oil.

Data for 19 and 25:

R_f: 0.35 (4:1 petrol/EtOAc).
IR (neat) ν_{max}: 3417, 2966, 2914, 2856, 2360, 2342, 1664, 1440, 1363, 1346, 1224, 1170, 1067, 1024, 831, 803, 734 cm^{-1}.
^{1}H NMR (500 MHz, $CDCl_3$) δ: 6.58 (s, 1H), 6.57 (s, 1H), 5.48 (s, 2H), 5.08–5.02 (m, 6H), 4.71–4.66 (m, 2H), 4.59 (br s, 2H), 3.50 (s, 2H), 3.28–3.20 (m, 2H), 3.12–3.05 (m, 4H), 2.64–2.57 (m, 2H), 2.18 (d, *J* = 10.5 Hz, 2H), 2.09 (d, *J* = 16.8 Hz, 2H), 2.05–1.96 (m, 10H), 1.94–1.88 (m, 8H), 1.78 (d, *J* = 18.9 Hz, 2H), 1.72 (s, 9H), 1.71 (s, 9H), 1.68 (s, 3H), 1.66 (m, 2H), 1.64 (s, 3H), 1.63 (s, 3H), 1.61 (s, 6H), 1.60 (s, 3H), 1.58 (s, 3H), 1.56 (s, 3H), 1.47 (s, 6H), 1.40 (s, 6H) ppm.
^{13}C NMR (125 MHz, $CDCl_3$) δ: 199.8, 199.7, 197.9, 197.8, 153.5, 153.3, 138.9, 138.9, 138.7, 138.0, 135.1, 134.3, 132.1, 131.9, 131.6, 131.5, 126.9, 126.9, 124.4, 124.4, 124.3, 124.3, 124.2, 124.2, 123.0, 121.1, 121.0, 121.0, 118.8, 118.7, 118.4, 118.3, 118.3, 80.9, 80.8, 56.9, 56.8, 55.6, 55.5, 40.1, 40.0, 39.7, 39.1, 39.0, 30.2, 28.1, 26.7, 26.6, 26.3, 26.1, 25.9, 25.8, 25.8, 23.6, 23.5, 22.9, 18.0, 17.9, 17.8, 17.8, 16.7, 16.7, 16.4, 16.3 ppm.
HRMS (ESI) *m/z*: Calculated for $C_{36}H_{53}O_4$ $[M+H]^+$ 549.3938; found 549.3940.

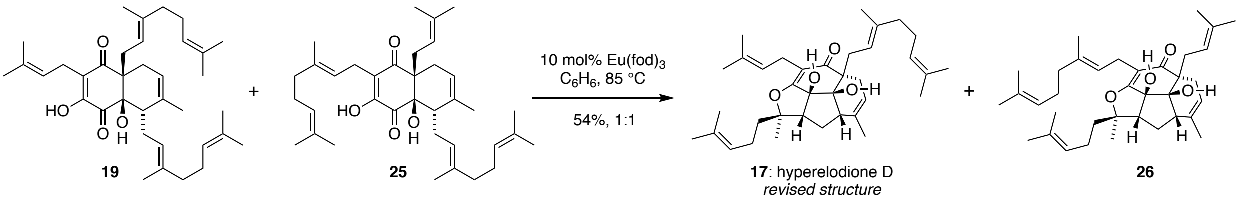

A solution of *endo* adducts **19** and **25** (43.4 mg, 0.0791 mmol) and Eu(fod)$_3$ (8.2 mg, 0.0079 mmol) in benzene (0.8 mL) was stirred at 85 °C for 16 h, then cooled to rt. The resulting solution was diluted with water (10 mL) and extracted with EtOAc (2 × 15 mL). The organic extracts were washed with brine (25 mL), dried over $MgSO_4$, filtered and concentrated in vacuo. The crude residue was subjected to flash chromatography over SiO_2 (petrol/EtOAc, 1:0 → 4:1 gradient elution) to give a 1:1 mixture of hyperelodione D (**17**) and tetracycle **26** (23.7 mg, 54%) as a clear oil. Separation of the isomers was achieved through further chromatography, affording hyperelodione D (**17**) (8.6 mg, 19%) and tetracycle **26** (9.4 mg, 21%) as clear oils.

Data for 17:

R$_f$: 0.35 (4:1 petrol/EtOAc).
IR (neat) ν_{max}: 3411, 2971, 2924, 2857, 1617, 1540, 1438, 1379, 1225, 1191, 1157, 1082, 958, 857, 669 cm^{-1}.
^{1}H NMR (500 MHz, CDCl$_3$) δ: 5.43–5.38 (m, 1H), 5.16–5.11 (m, 2H), 5.06–4.99 (m, 2H), 3.39 (s, 1H), 3.26 (s, 1H), 2.98–2.86 (m, 2H), 2.67–2.59 (m, 3H), 2.56 (dd, *J* = 11.3, 7.4 Hz, 1H), 2.29 (dd, *J* = 15.1, 5.6 Hz, 1H), 2.18–1.94 (m, 10H), 1.69 (s, 3H), 1.67 (s, 9H), 1.62 (s, 3H), 1.61 (s, 3H), 1.57 (s, 3H), 1.52 (s, 3H), 1.34 (s, 3H), 1.15–1.06 (m, 1H) ppm.
^{13}C NMR (125 MHz, CDCl$_3$) δ: 199.1, 170.4, 140.3, 134.4, 132.1, 132.0, 131.8, 124.1, 123.8, 122.2, 121.7, 121.5, 118.0, 92.8, 84.6, 79.2, 52.4, 51.7, 50.3, 40.0, 39.3, 35.3, 31.2, 29.4, 26.5, 25.8, 25.8, 25.8, 23.8, 22.1, 21.9, 20.1, 17.9, 17.8, 16.8 ppm.
HRMS (ESI) *m/z*: Calculated for $C_{36}H_{52}O_4Na$ [M+Na]$^+$ 571.3758; found 571.3763.

Data for 26:

R$_f$: 0.30 (4:1 petrol/EtOAc).
IR (neat) ν_{max}: 3401, 2971, 2910, 2856, 1617, 1540, 1445, 1439, 1378, 1345, 1256, 1225, 1189, 1082, 1021, 958, 857, 809, 669 cm^{-1}.
^{1}H NMR (500 MHz, CDCl$_3$) δ: 5.41–5.37(m, 1H), 5.17–5.05 (m, 3H), 5.03 (t, *J* = 7.0 Hz, 1H), 3.39 (s, 1H), 3.24 (s, 1H), 2.98–2.89 (m, 2H), 2.66–2.54 (m, 4H), 2.29 (dd, *J* = 14.9, 4.3 Hz, 1H), 2.18–1.97 (m, 8H), 1.93–1.89 (m, 2H), 1.70 (s, 6H), 1.68 (s, 3H), 1.66 (s, 6H), 1.62 (s, 3H), 1.57 (s, 3H), 1.52 (s, 3H), 1.34 (s, 3H), 1.14–1.06 (m, 1H) ppm.
^{13}C NMR (125 MHz, CDCl$_3$) δ: 198.9, 170.4, 136.7, 135.5, 134.3, 132.1, 131.4, 124.6, 124.1, 122.4, 121.5, 121.4, 117.9, 92.8, 84.6, 79.2, 52.3, 51.7, 50.4, 39.9, 39.3, 35.3, 31.1, 29.5, 27.1, 26.1, 25.8, 23.8, 22.0, 21.9, 20.1, 18.3, 17.8, 16.3 ppm.
HRMS (ESI) *m/z*: Calculated for $C_{36}H_{52}O_4Na$ [M+Na]$^+$ 571.3758; found 571.3783.

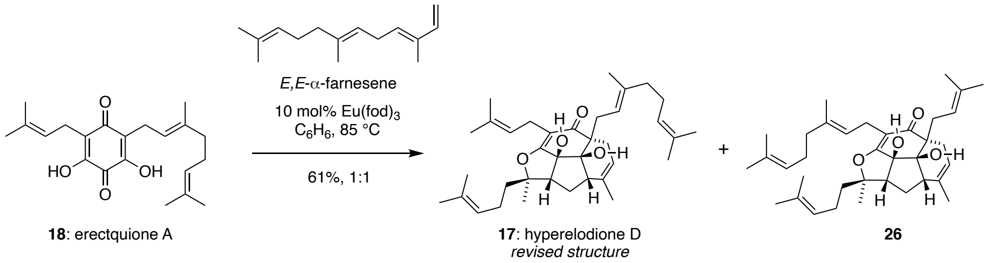

A solution of erectquione A (**18**) (56.3 mg, 0.163 mmol), *E,E*-α-farnesene (83.5 mg, 0.409 mmol) and Eu(fod)$_3$ (17.0 mg, 0.0163 mmol) in benzene (1.6 mL) was stirred at 85 °C for 16 h, then cooled to rt. The resulting solution was diluted with water (10 mL) and extracted with EtOAc (2 × 15 mL). The organic extracts were washed with brine (25 mL), dried over $MgSO_4$, filtered and concentrated in vacuo. The crude residue was subjected to flash chromatography over SiO_2 (petrol/EtOAc, 1:0 → 4:1 gradient elution) to give a 1:1 mixture of hyperelodione D (**17**) and tetracycle **26** (53.4 mg, 61%) as a clear oil. Separation of the isomers was achieved through further chromatography, affording hyperelodione D (**17**) (22.5 mg, 26%) and tetracycle **26** (19.4 mg, 22%) as clear oils.

3. NMR Spectra

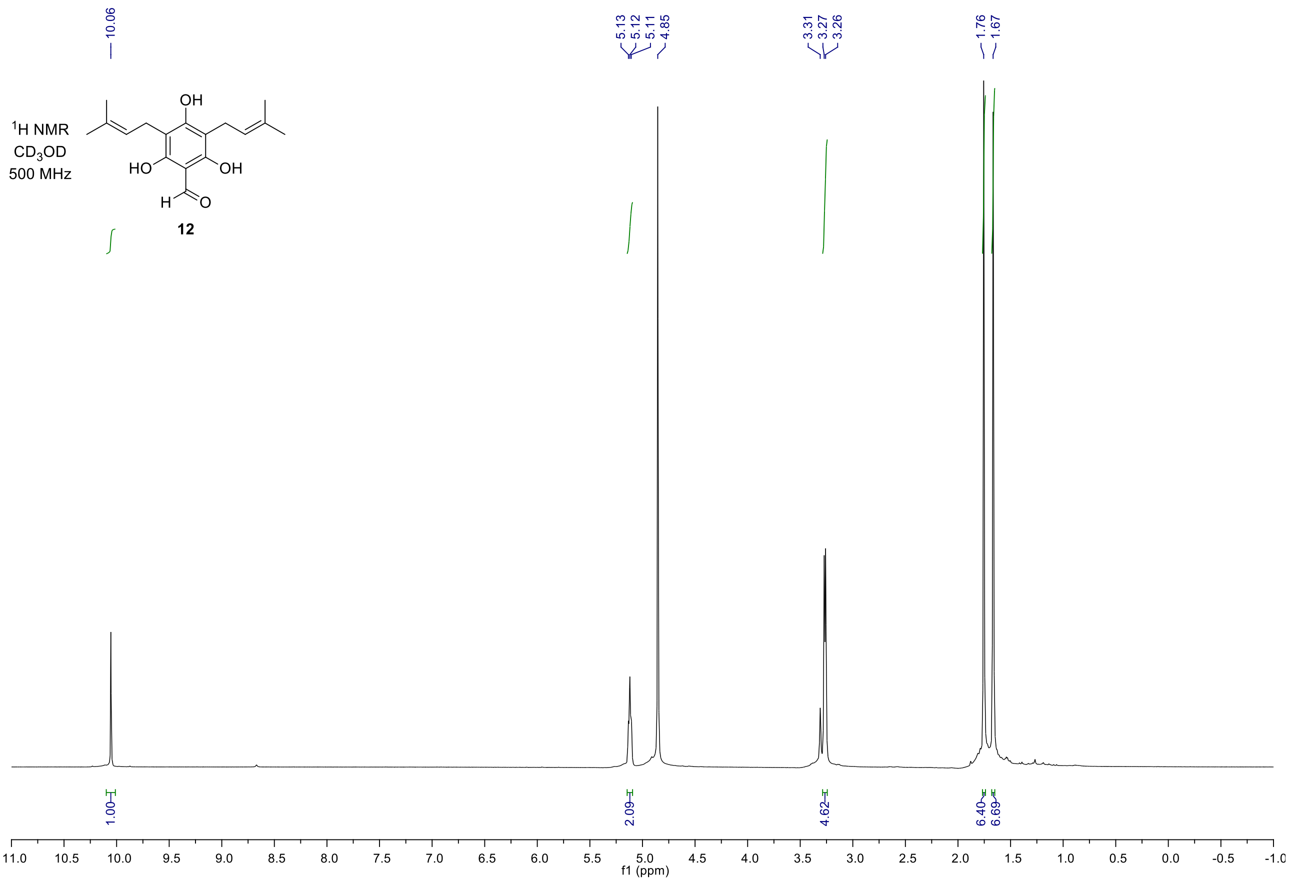

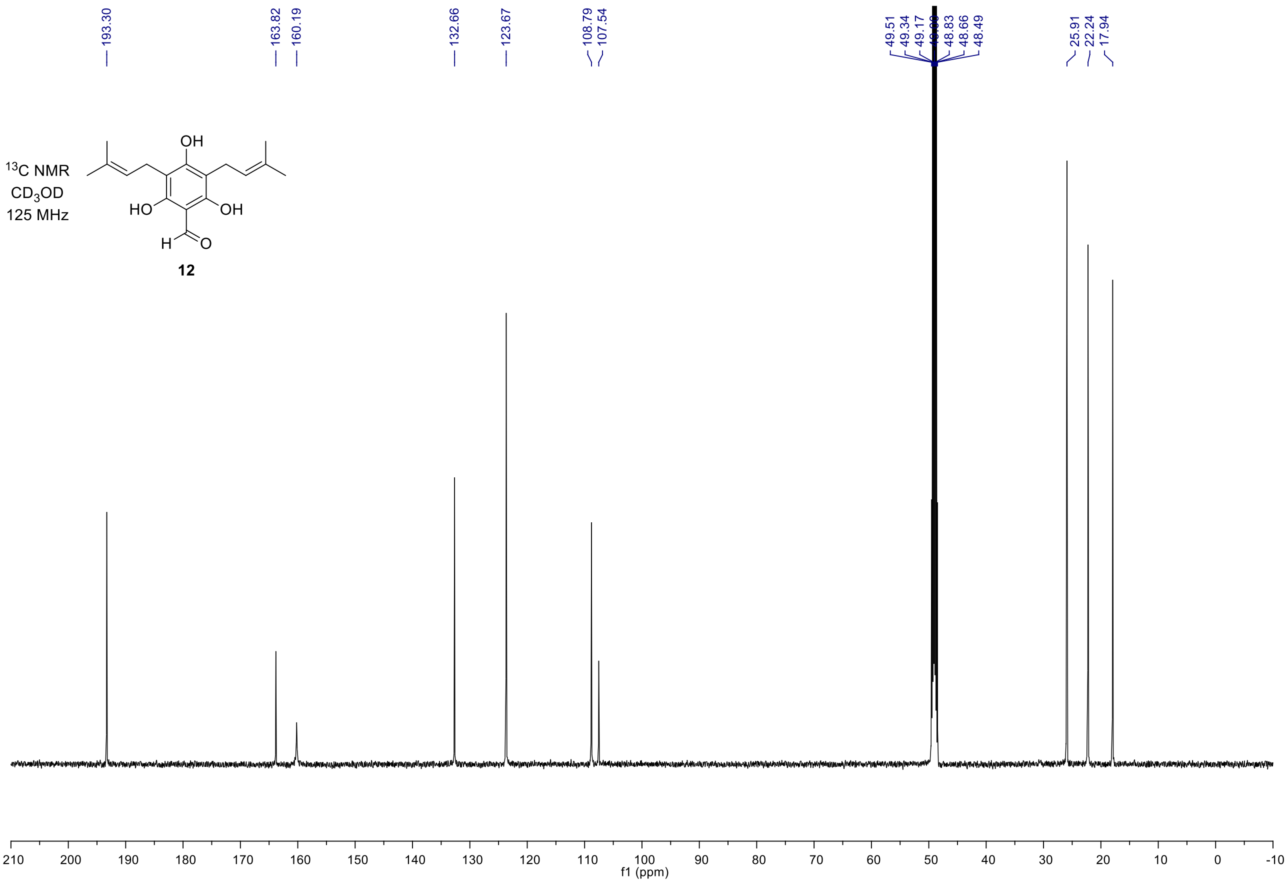

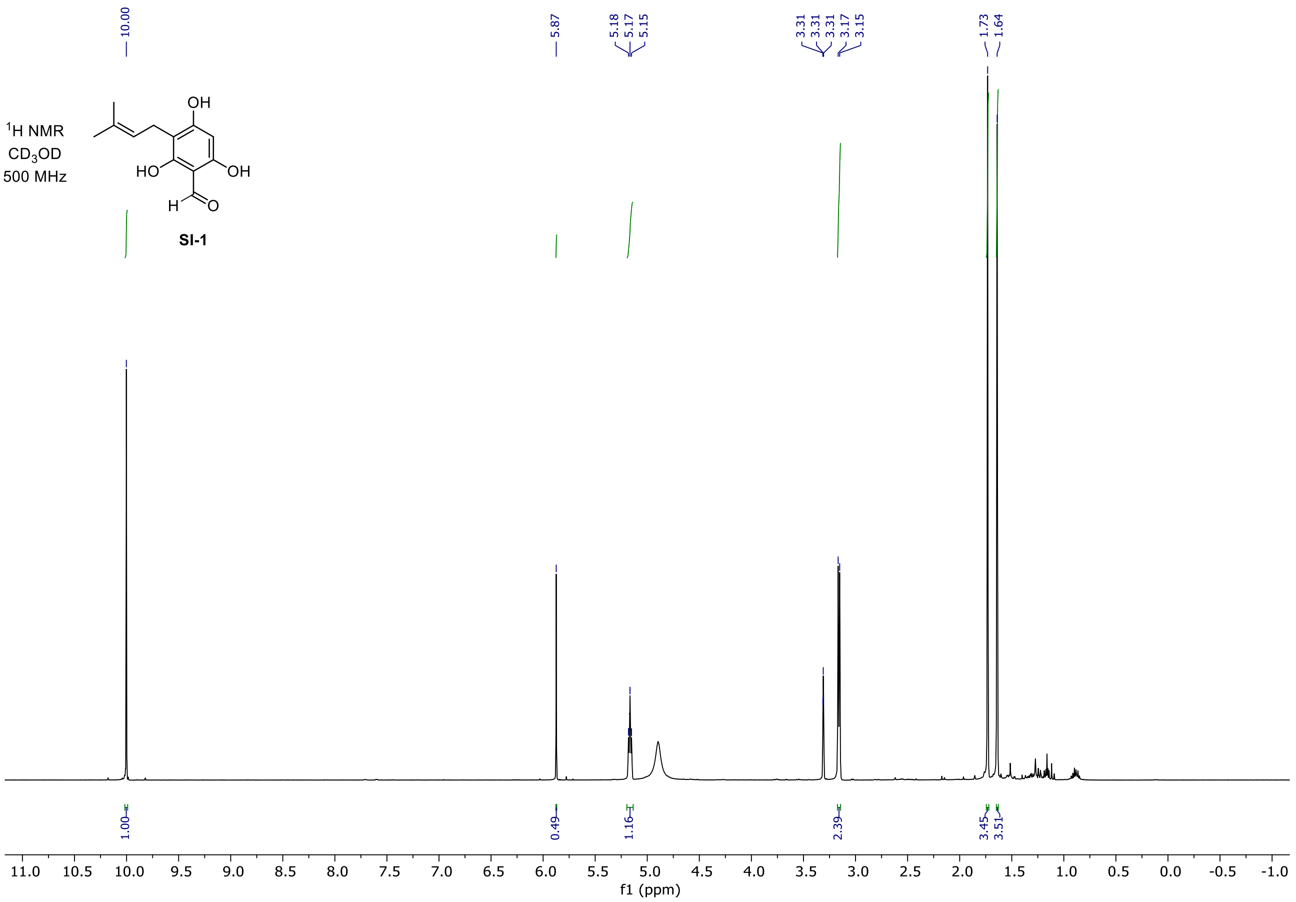
^{1}H NMR
CD_3OD
500 MHz
SI-1
10.00
5.87
5.18
5.17
5.15
3.31
3.31
3.31
3.17
3.15
1.73
1.64
1.00
0.49
1.16
2.39
3.45
3.51
f1 (ppm)

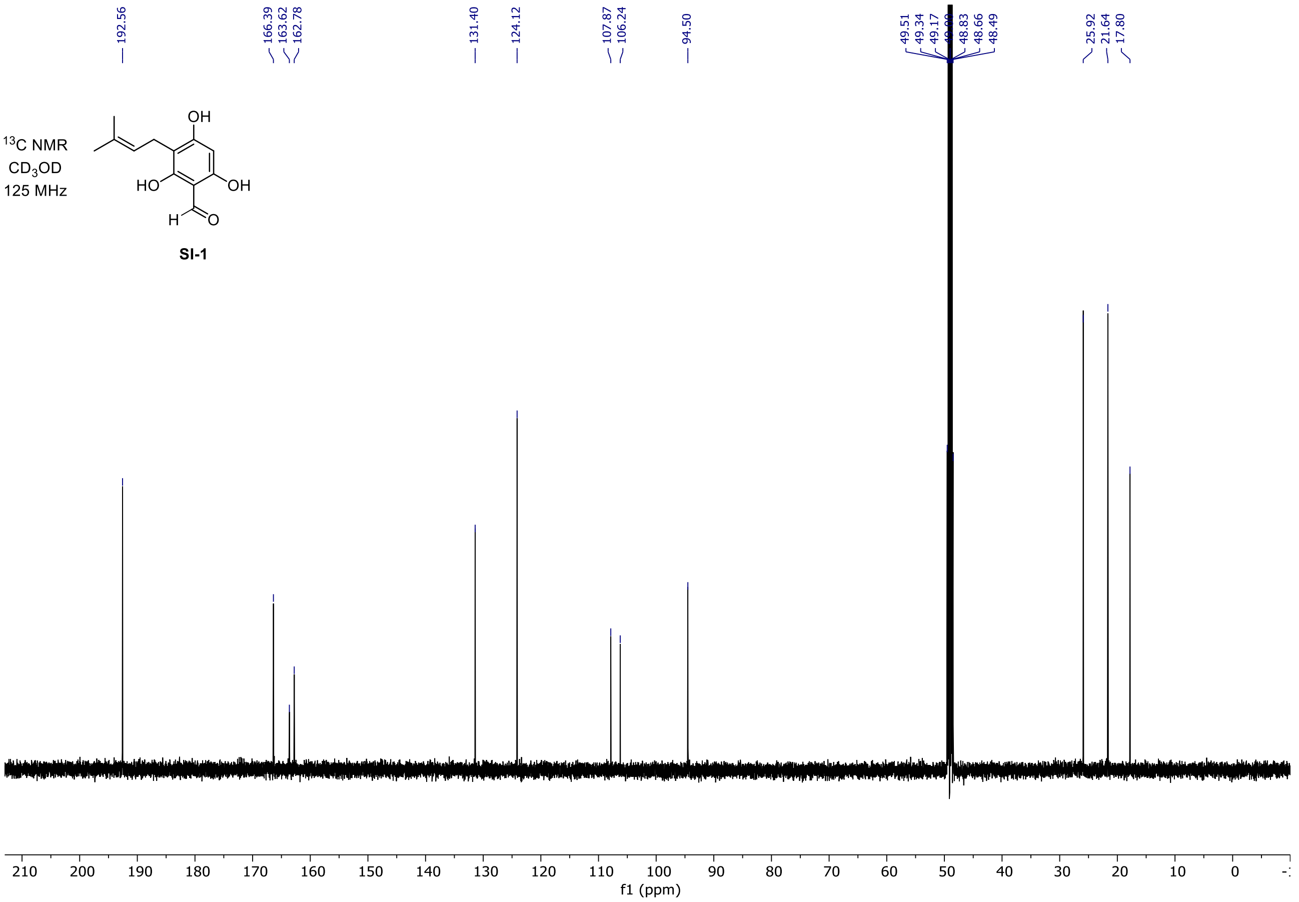
^{13}C NMR
CD_3OD
125 MHz
SI-1
192.56
166.39
163.62
162.78
131.40
124.12
107.87
106.24
94.50
49.51
49.34
49.17
48.83
48.66
48.49
25.92
21.64
17.80
f1 (ppm)

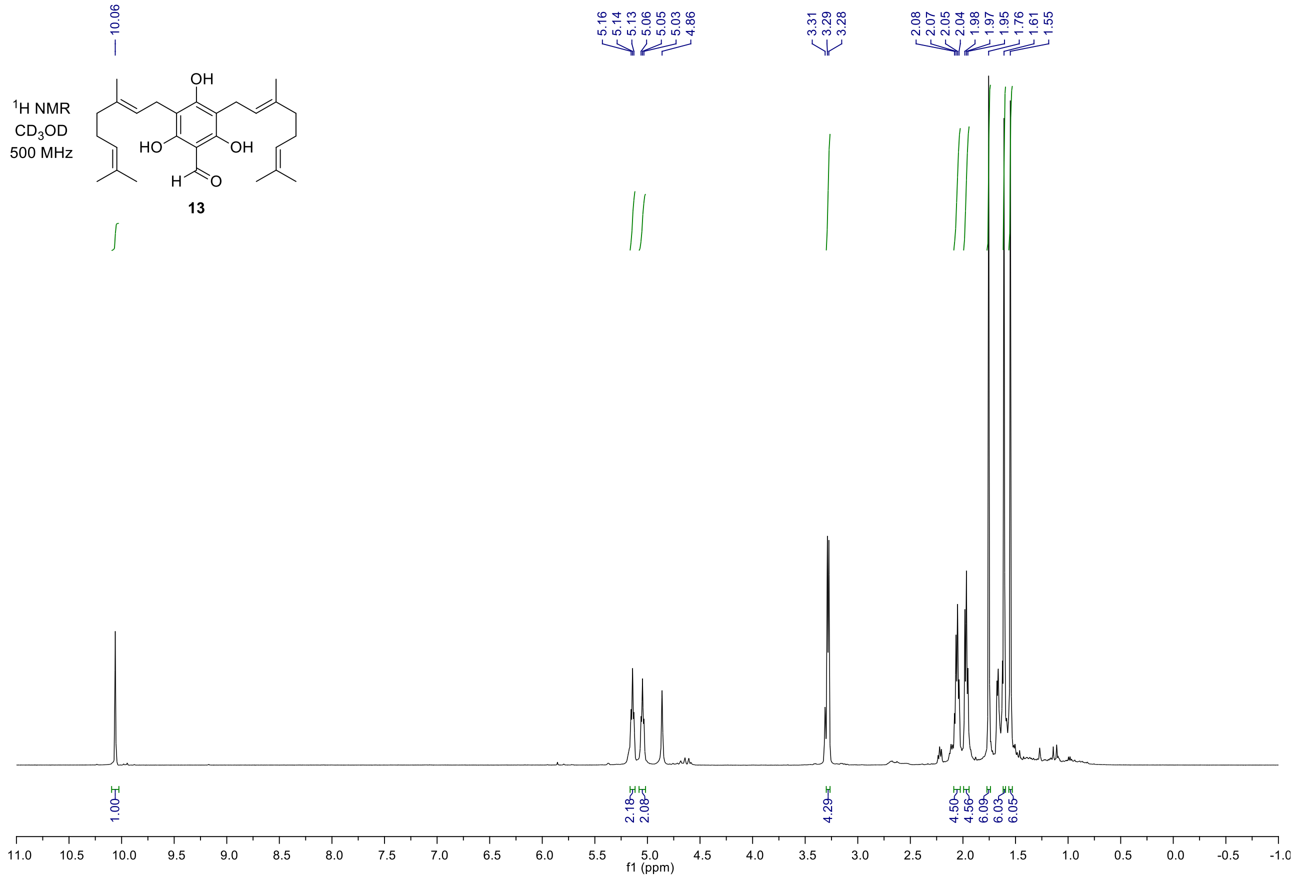

1H NMR
CD3OD
500 MHz
13
10.06
5.16
5.14
5.13
5.06
5.05
5.03
4.86
3.31
3.29
3.28
2.08
2.07
2.05
2.04
1.98
1.97
1.95
1.76
1.61
1.55
1.00
2.18
2.08
4.29
4.50
4.56
6.09
6.03
6.05
f1 (ppm)

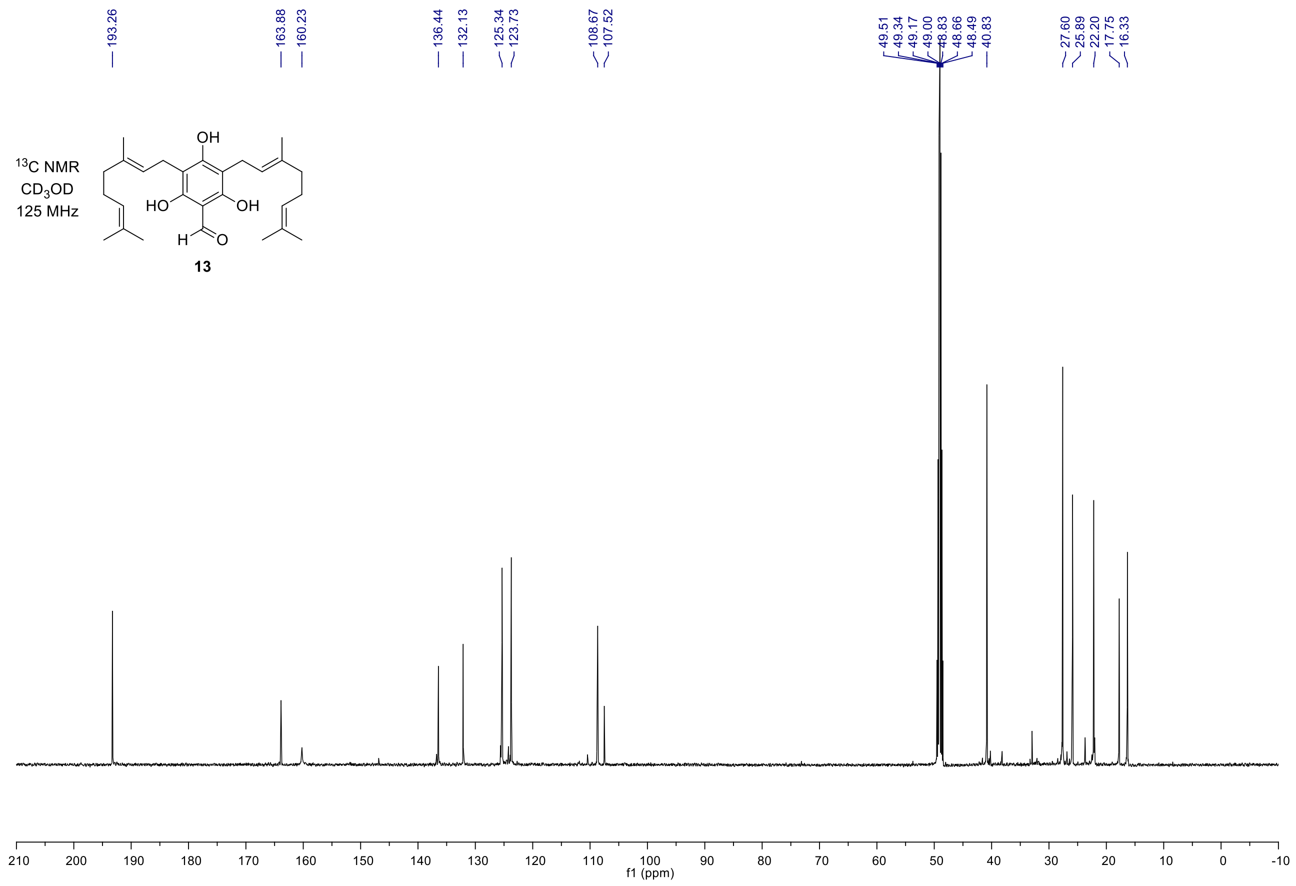

13C NMR
CD3OD
125 MHz
13
193.26
163.88
160.23
136.44
132.13
125.34
123.73
108.67
107.52
49.51
49.34
49.17
49.00
48.83
48.66
48.49
40.83
27.60
25.89
22.20
17.75
16.33
f1 (ppm)

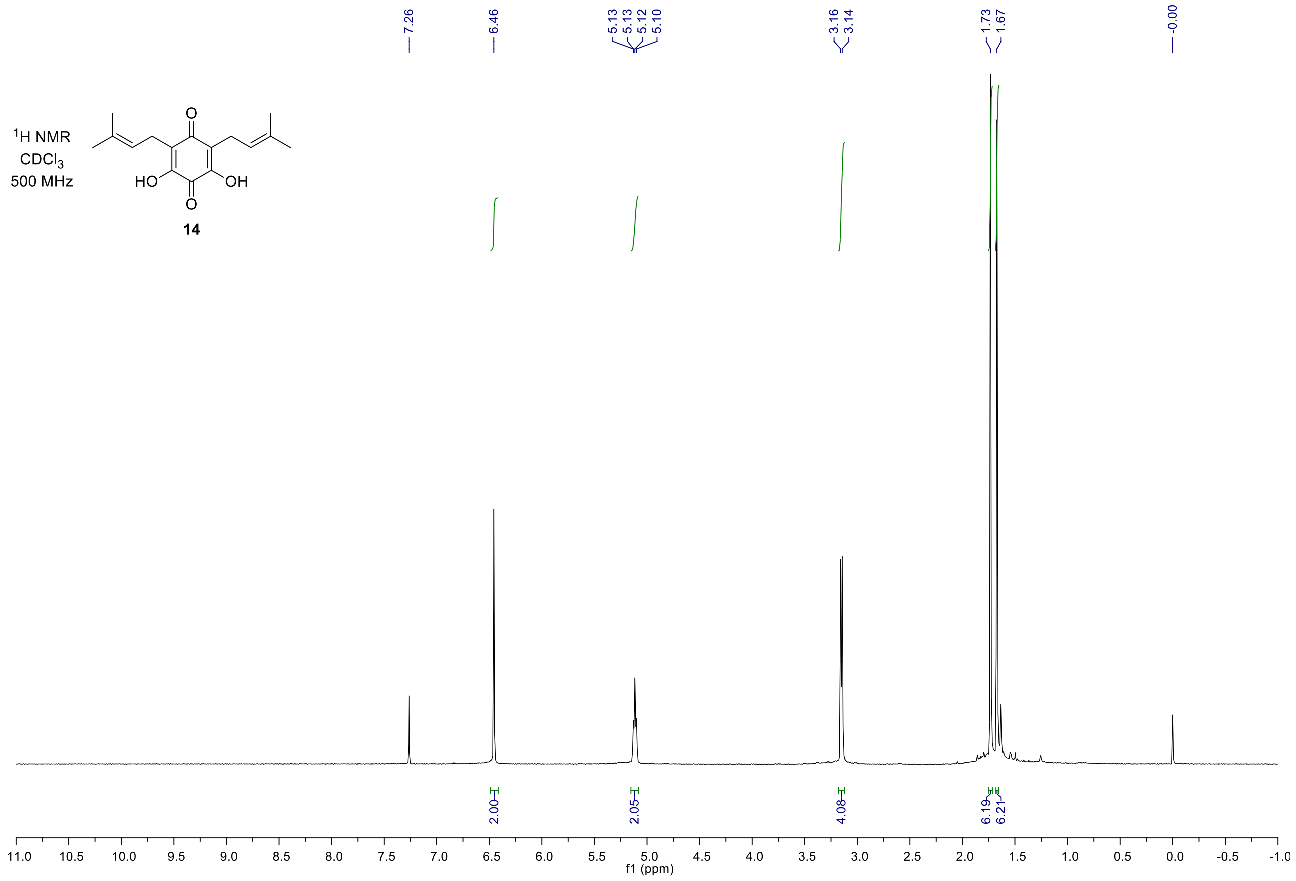

7.26
6.46
5.13
5.13
5.12
5.10
3.16
3.14
1.73
1.67
-0.00
¹H NMR
CDCl3
500 MHz
14
2.00
2.05
4.08
6.19
6.21
f1 (ppm)

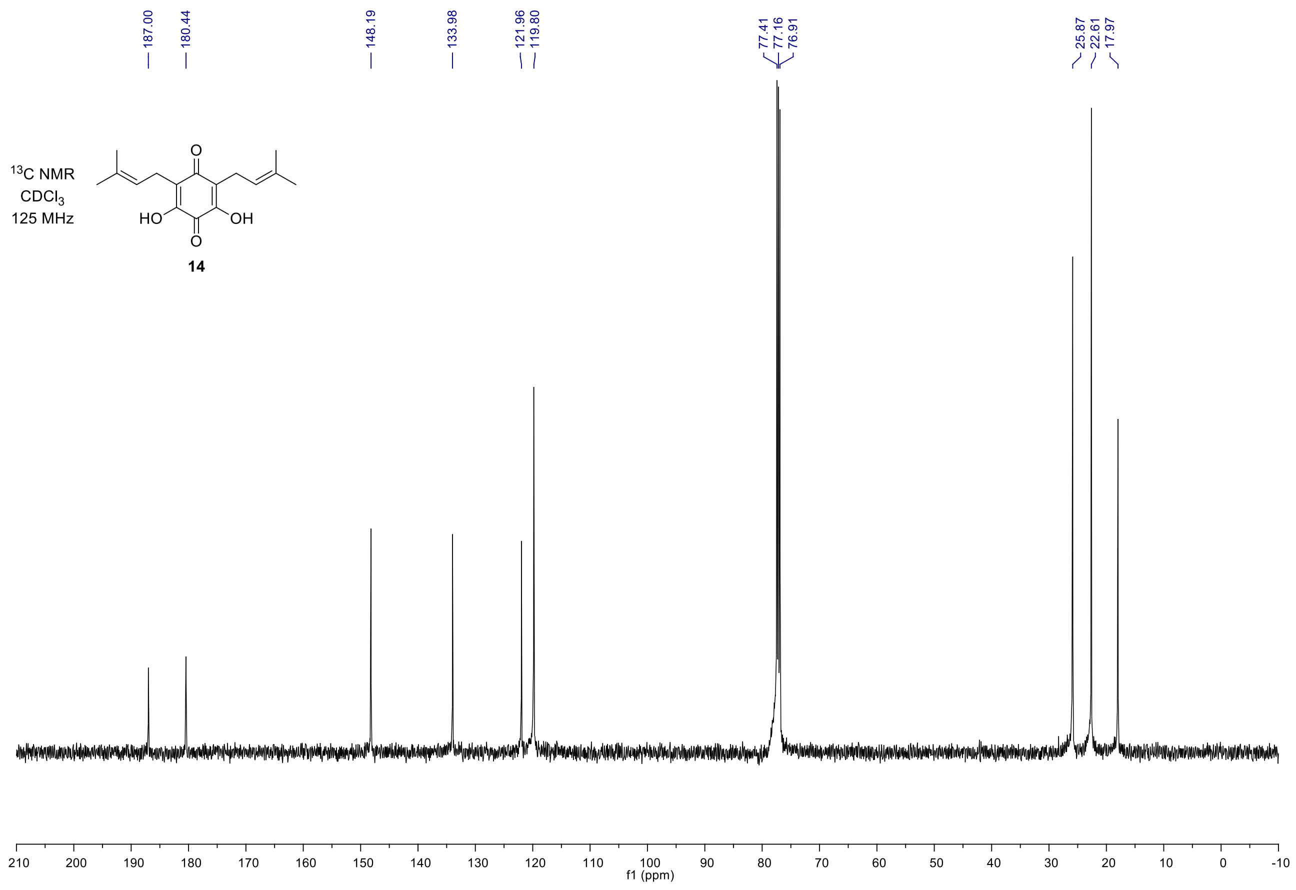

187.00
180.44
148.19
133.98
121.96
119.80
77.41
77.16
76.91
25.87
22.61
17.97
¹³C NMR
CDCl3
125 MHz
14
f1 (ppm)

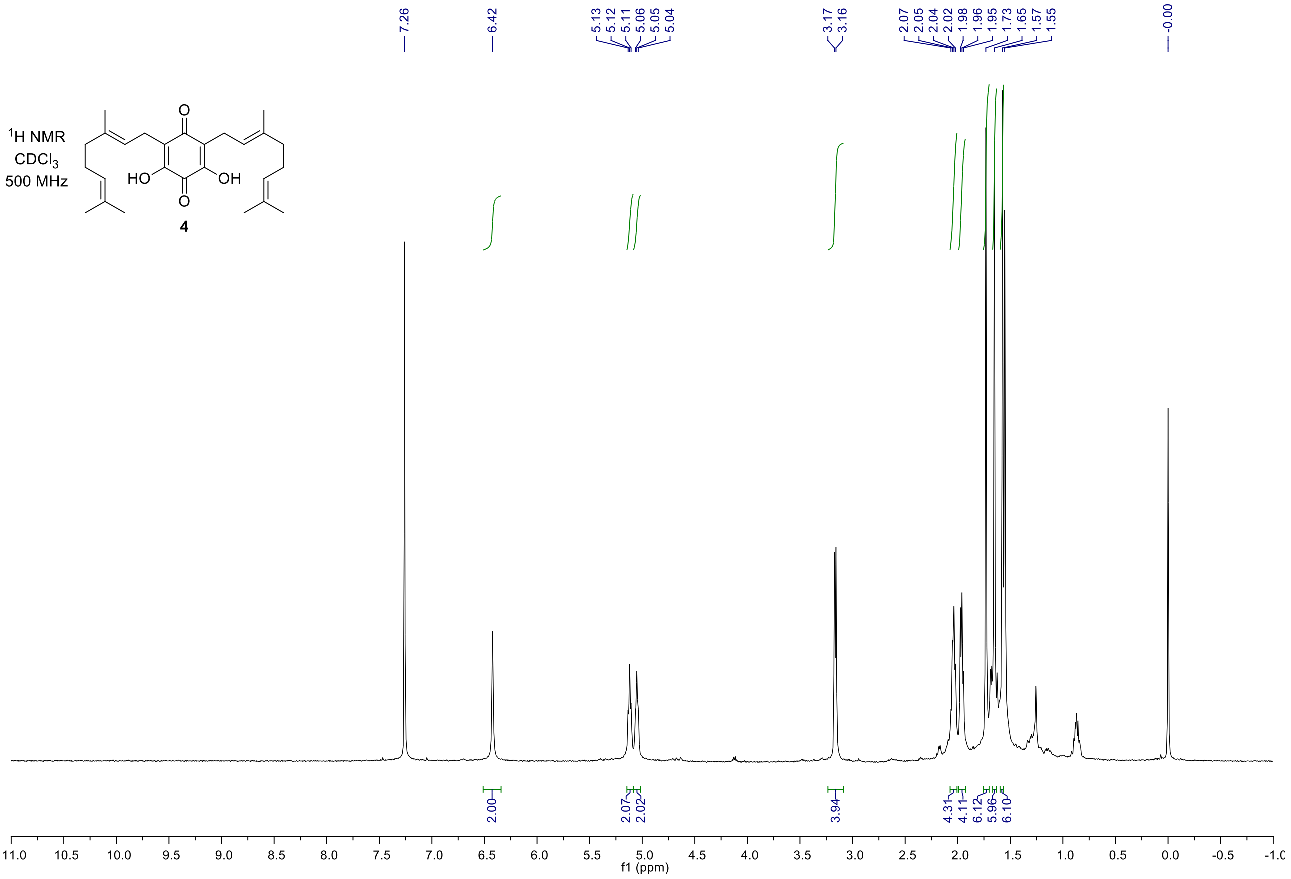

7.26
6.42
5.13 5.12 5.11 5.06 5.05 5.04
3.17 3.16
2.07 2.05 2.04 2.02 1.98 1.96 1.95 1.73 1.65 1.57 1.55
-0.00
^{1}H NMR
$CDCl_3$
500 MHz
HO
OH
O
O
4
2.00
2.07
2.02
3.94
4.31
4.11
6.12
5.96
6.10
f1 (ppm)

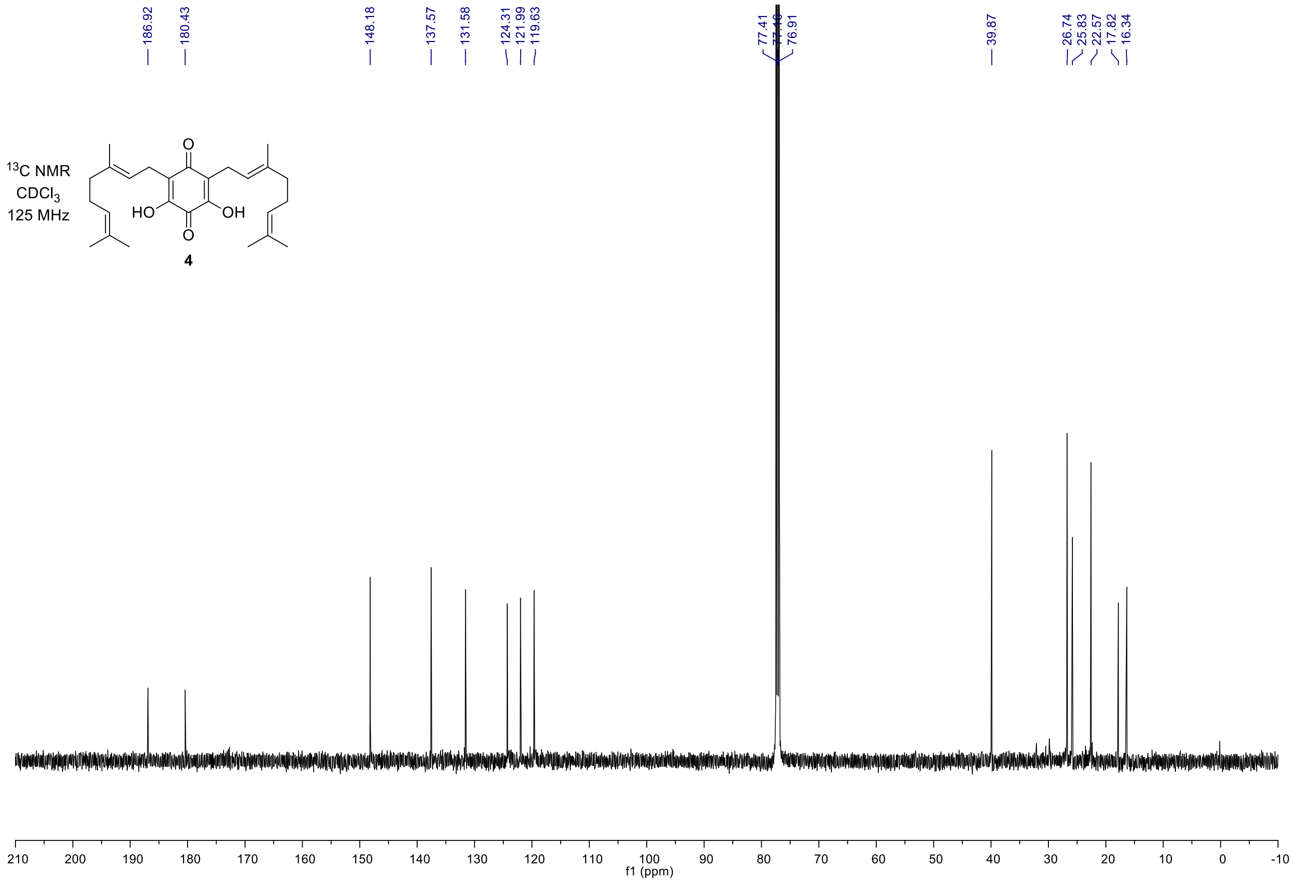

186.92
180.43
148.18
137.57
131.58
124.31 121.99 119.63
77.41 76.91
39.87
26.74 25.83 22.57 17.82 16.34
^{13}C NMR
$CDCl_3$
125 MHz
HO
OH
O
O
4
f1 (ppm)

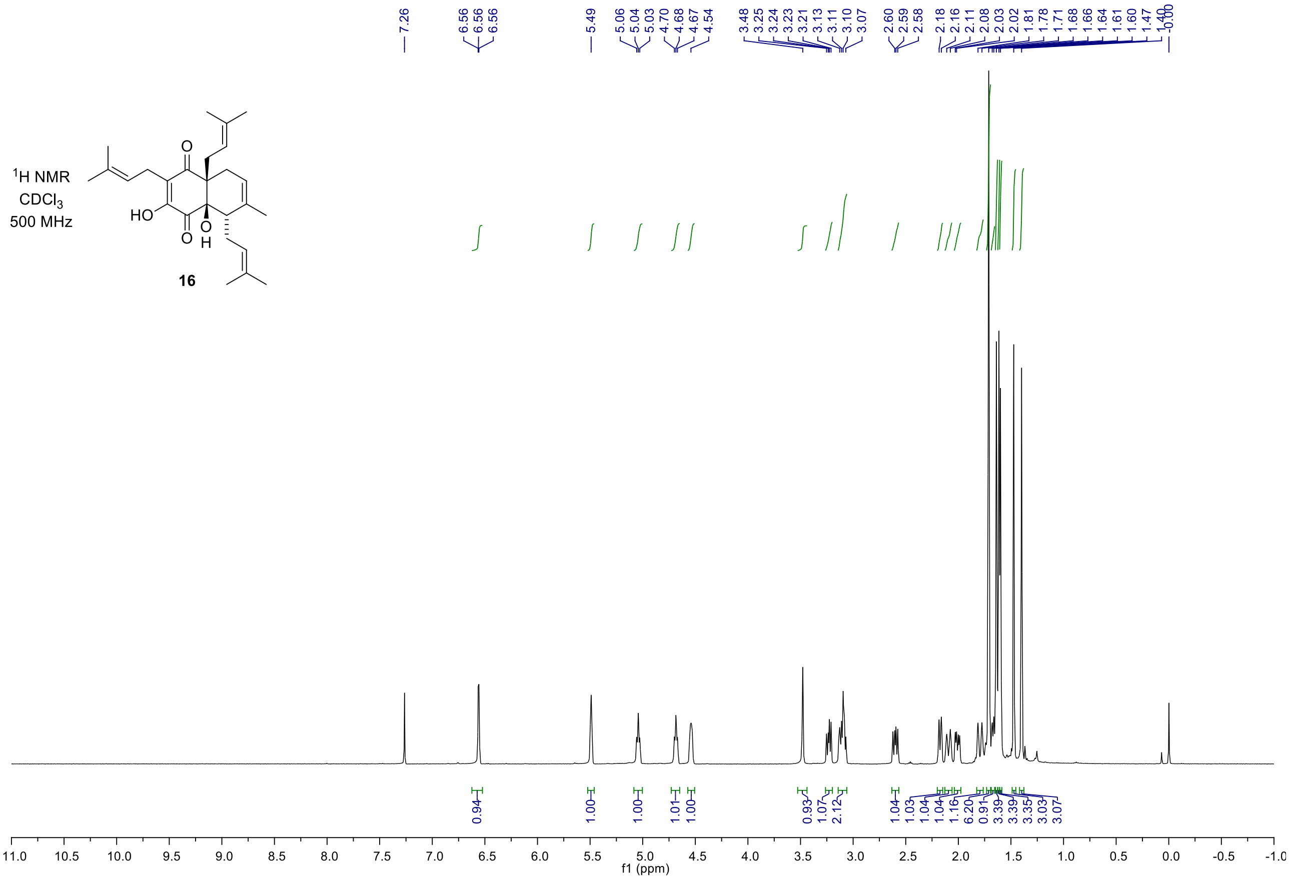

^{1}H NMR
CDCl3
500 MHz
16
7.26
6.56 6.56 6.56
5.49
5.06 5.04 5.03 4.70 4.68 4.67 4.54
3.48 3.25 3.24 3.23 3.21 3.13 3.11 3.10 3.07
2.60 2.59 2.58
2.18 2.16 2.11 2.08 2.03 2.02 1.81 1.78 1.71 1.68 1.66 1.64 1.61 1.60 1.47 1.40 0.00
0.94 1.00 1.00 1.01 1.00 0.93 1.07 2.12 1.04 1.03 1.04 1.04 1.16 6.20 0.91 3.39 3.39 3.35 3.03 3.07
f1 (ppm)

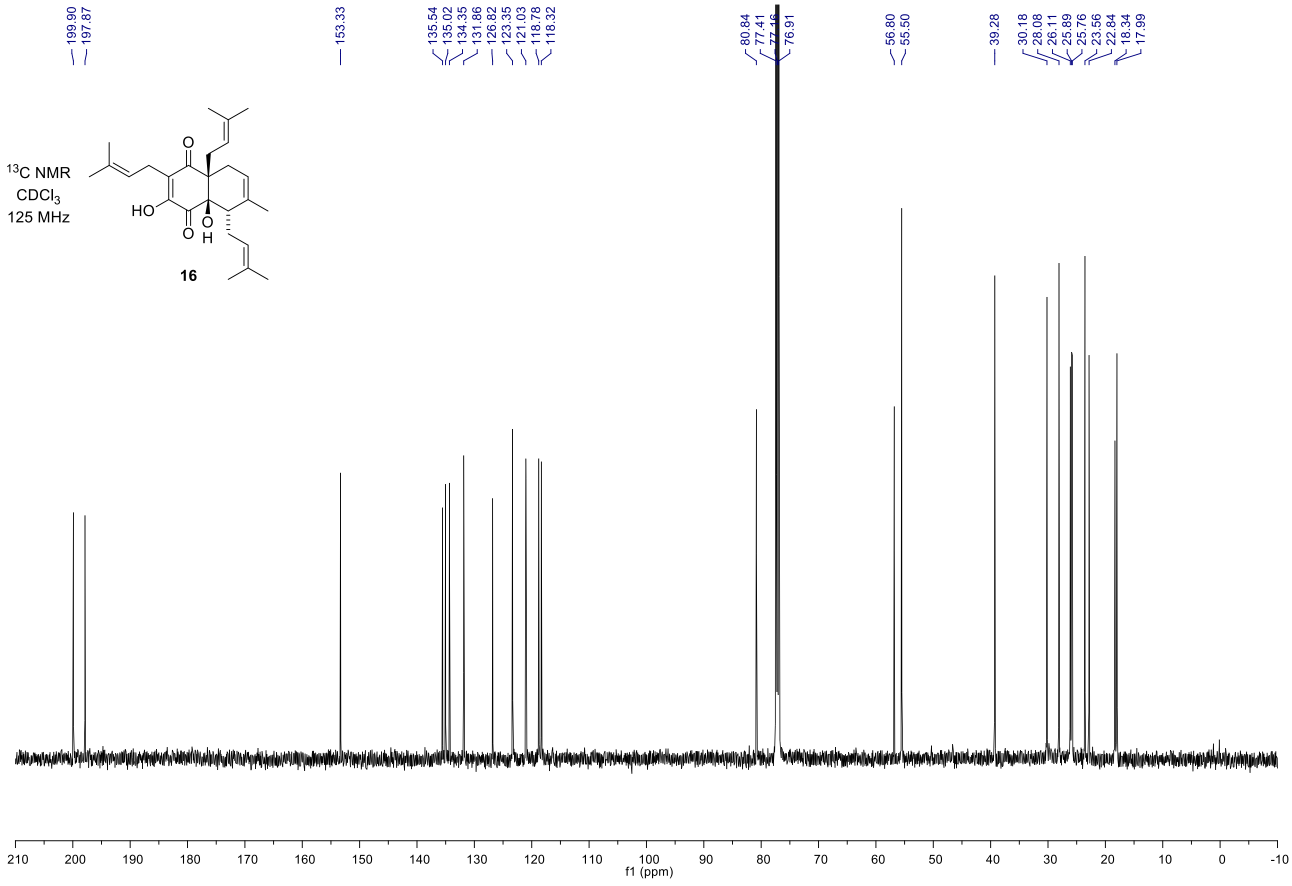

^{13}C NMR
CDCl3
125 MHz
16
199.90 197.87
153.33
135.54 135.02 134.35 131.86 126.82 123.35 121.03 118.78 118.32
80.84 77.41 77.16 76.91
56.80 55.50
39.28
30.18 28.08 26.11 25.89 25.76 23.56 22.84 18.34 17.99
f1 (ppm)

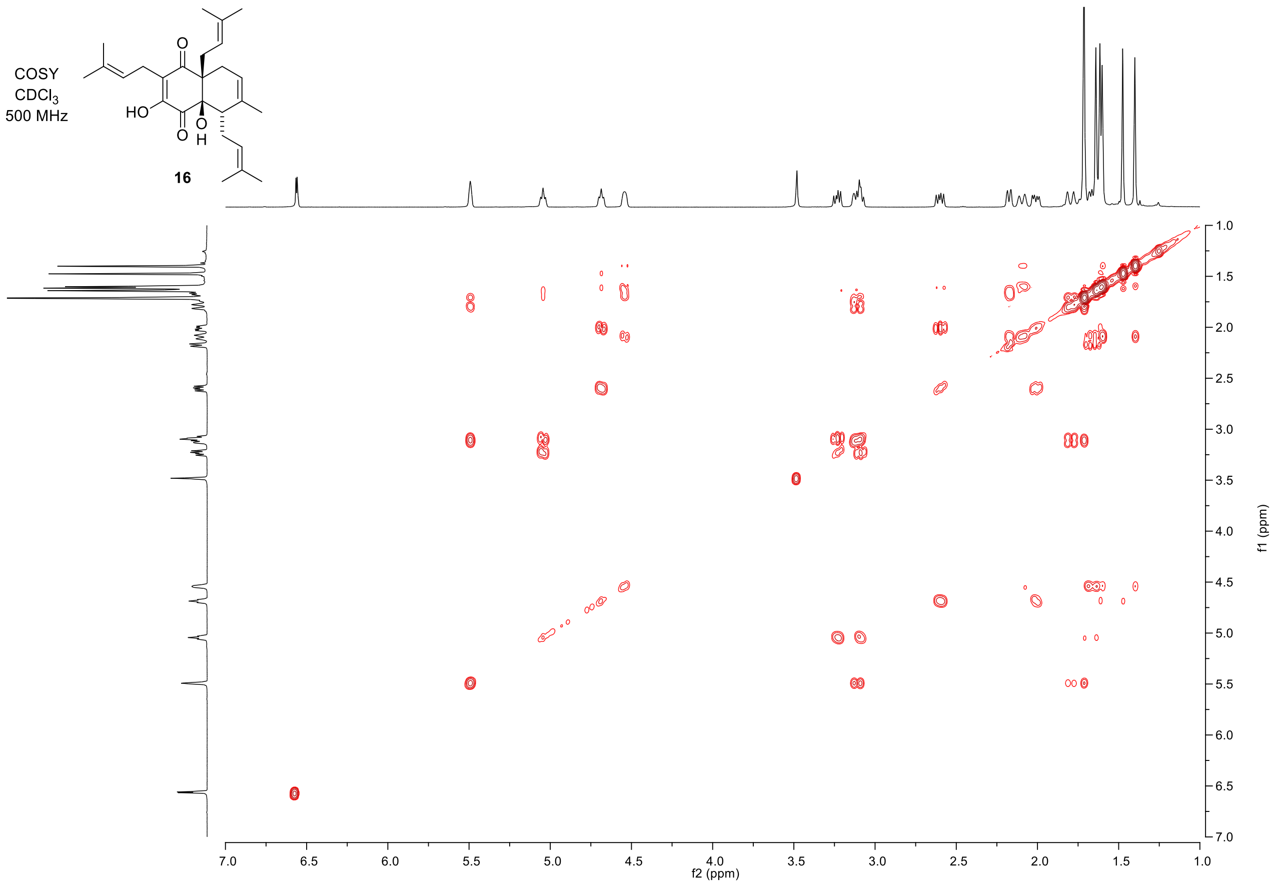
COSY
$CDCl_3$
500 MHz
HO
O
O
O
H
16
f2 (ppm)
f1 (ppm)

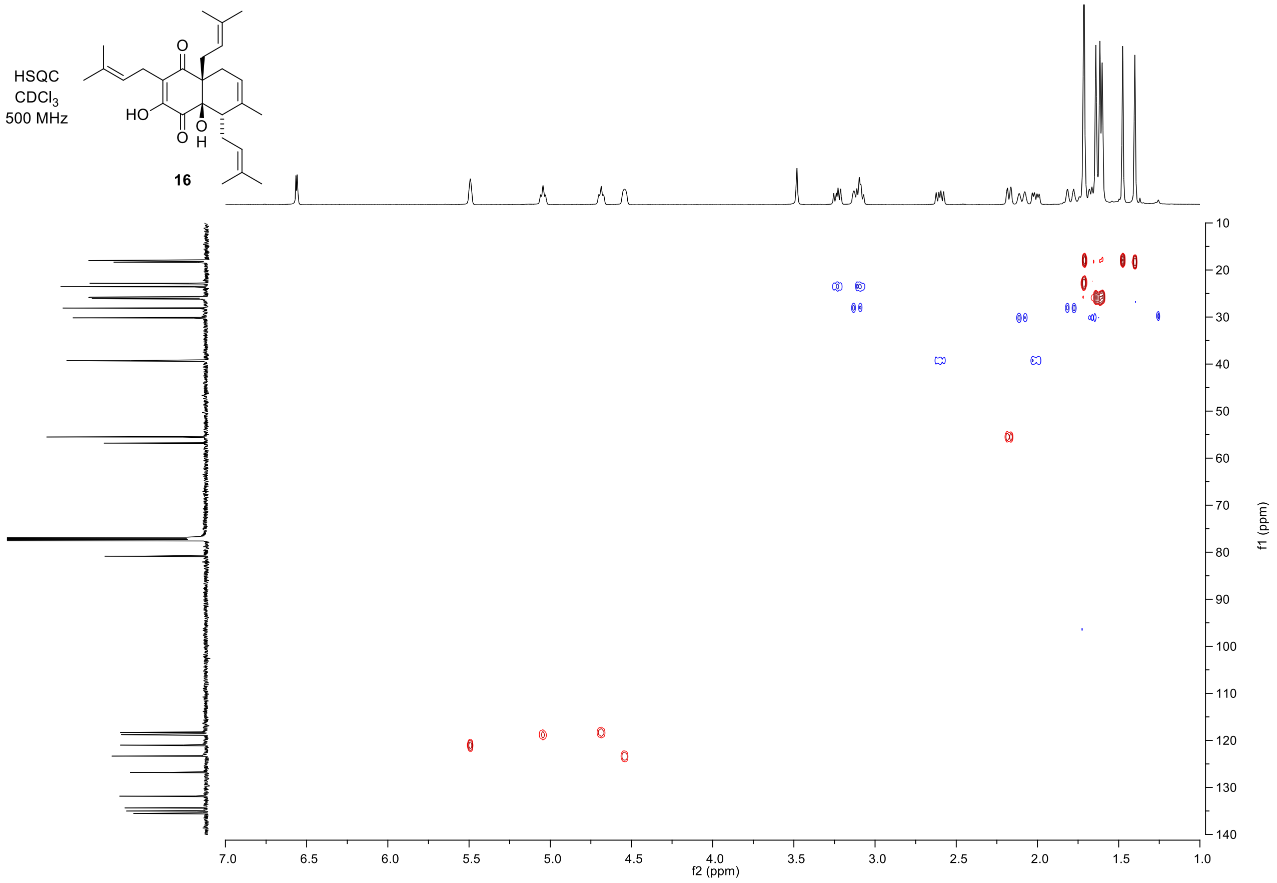
HSQC
$CDCl_3$
500 MHz
HO
O
O
O
H
16
f2 (ppm)
f1 (ppm)

HMBC
$CDCl_3$
500 MHz

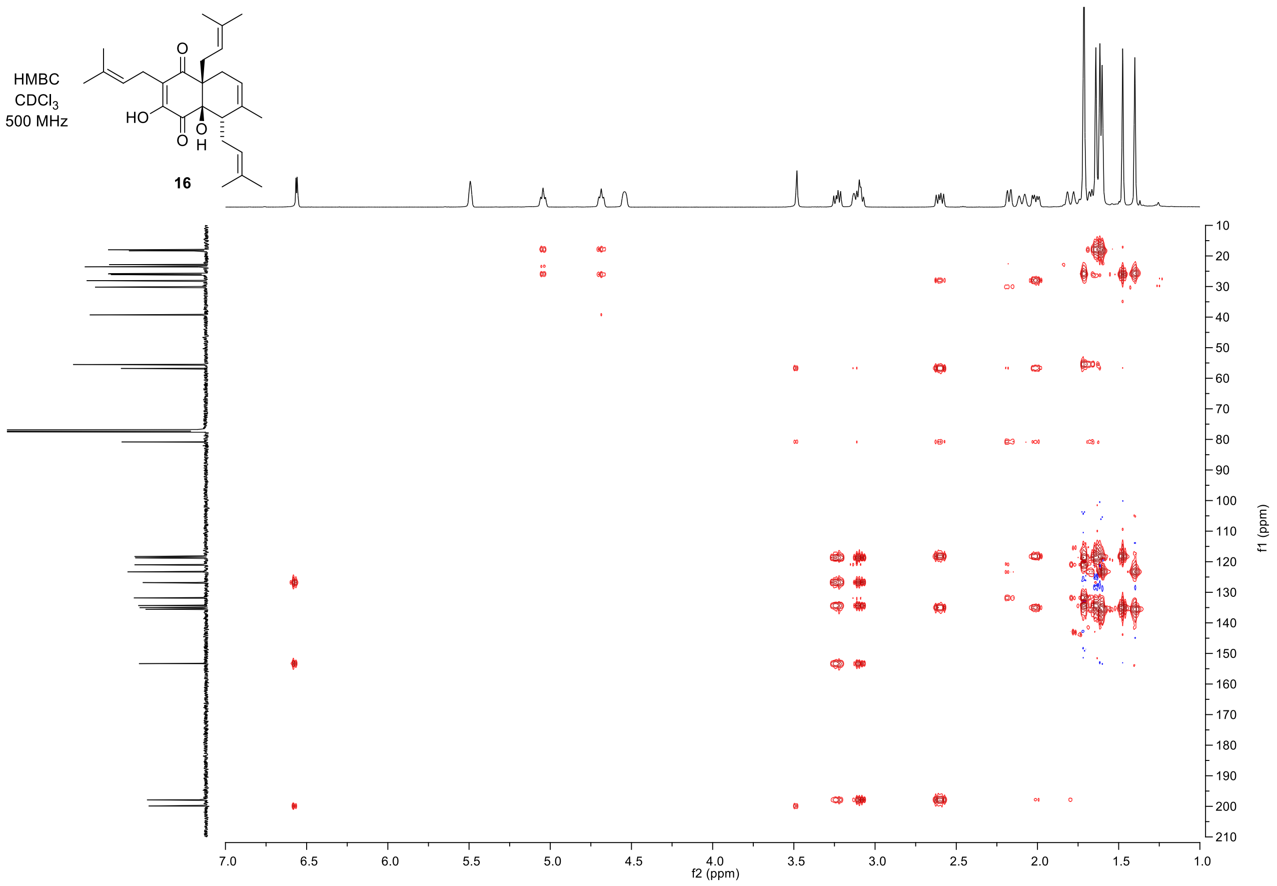

NOESY
$CDCl_3$
500 MHz

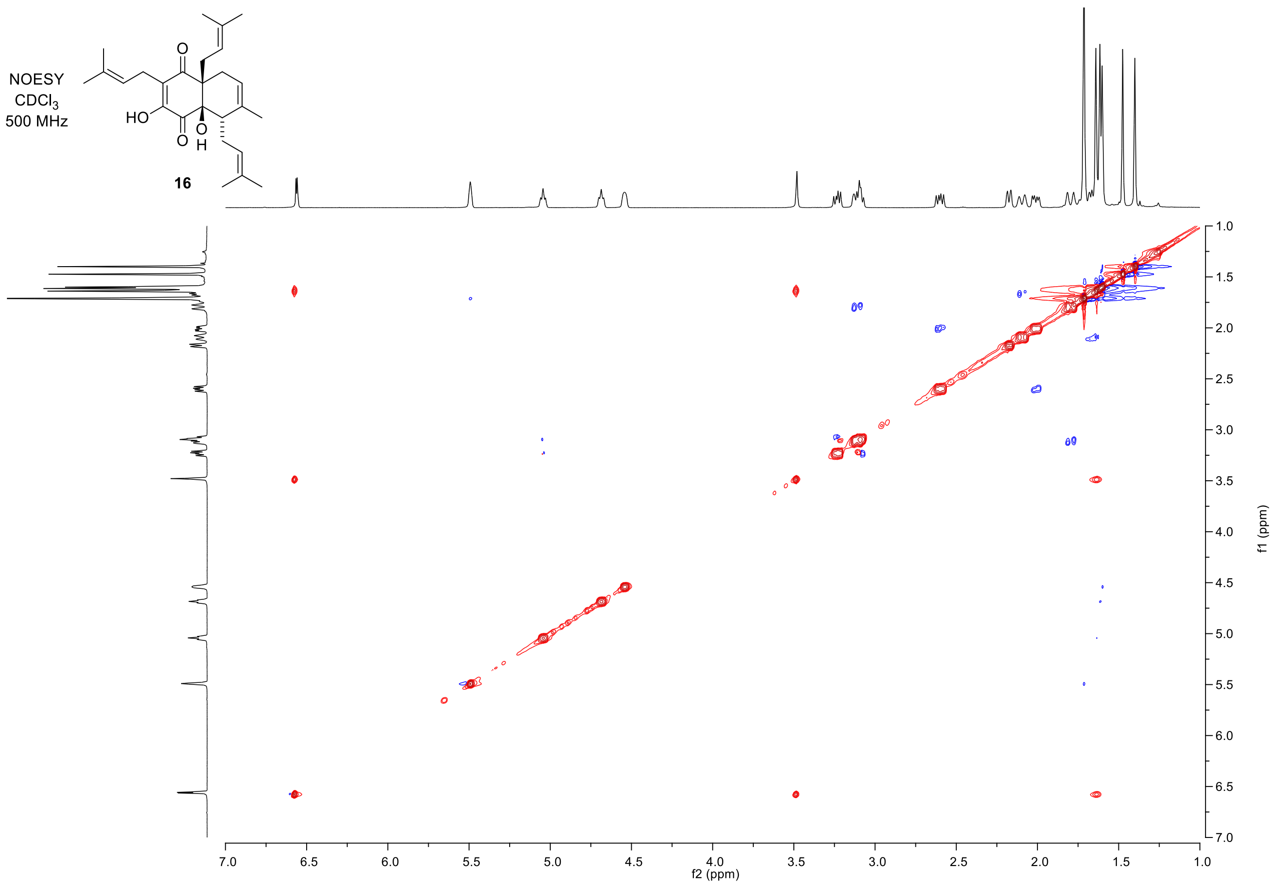

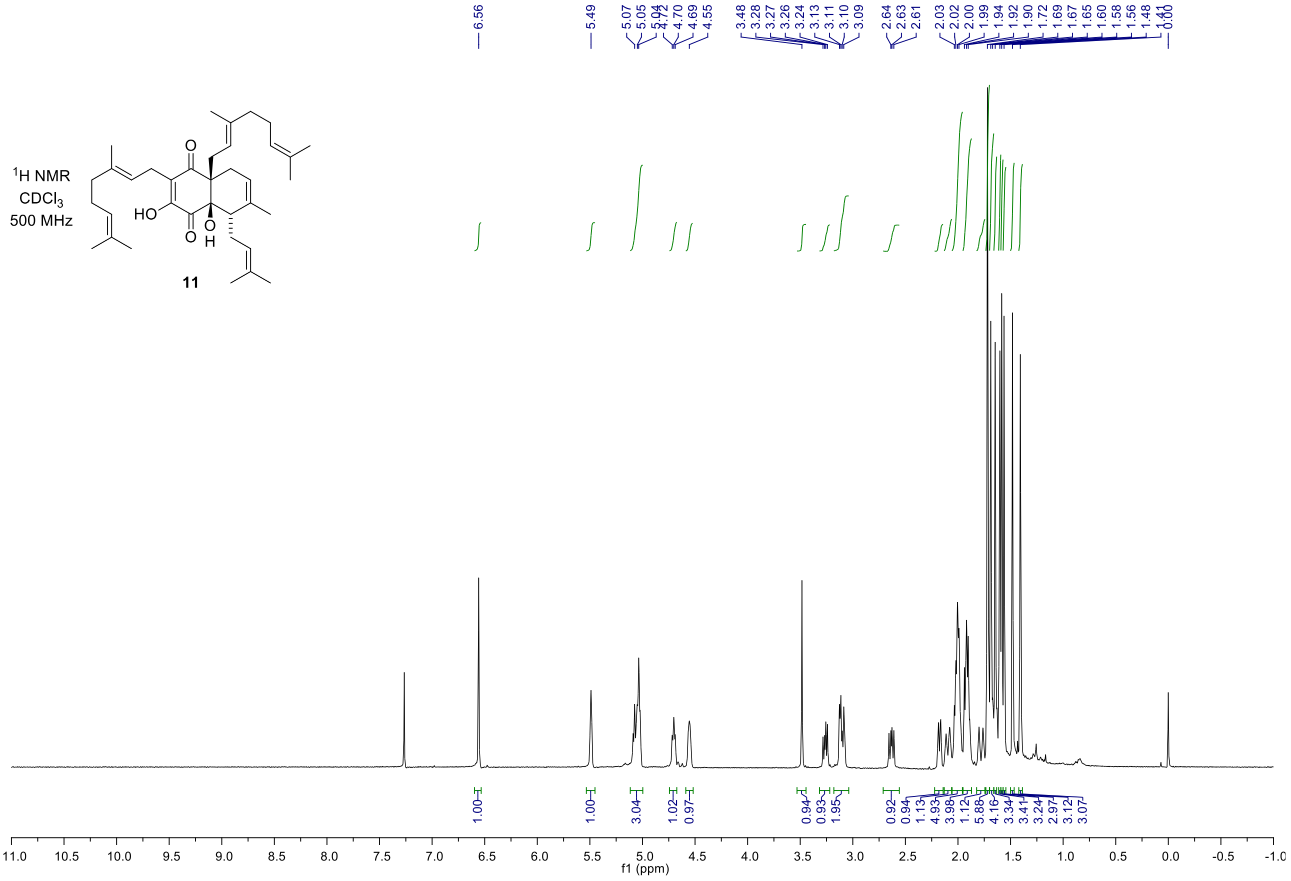

6.56
5.49
5.07
5.05
4.72
4.70
4.69
4.55
3.48
3.28
3.27
3.26
3.24
3.13
3.11
3.10
3.09
2.64
2.63
2.61
2.03
2.02
2.00
1.99
1.94
1.92
1.90
1.72
1.69
1.67
1.65
1.60
1.58
1.56
1.48
1.41
0.00
^{1}H NMR
$CDCl_3$
500 MHz
HO
O
H
11
1.00
1.00
3.04
1.02
0.97
0.94
0.93
1.95
0.92
0.94
1.13
4.93
3.98
1.12
5.88
4.16
3.34
3.41
3.24
2.97
3.12
3.07
f1 (ppm)

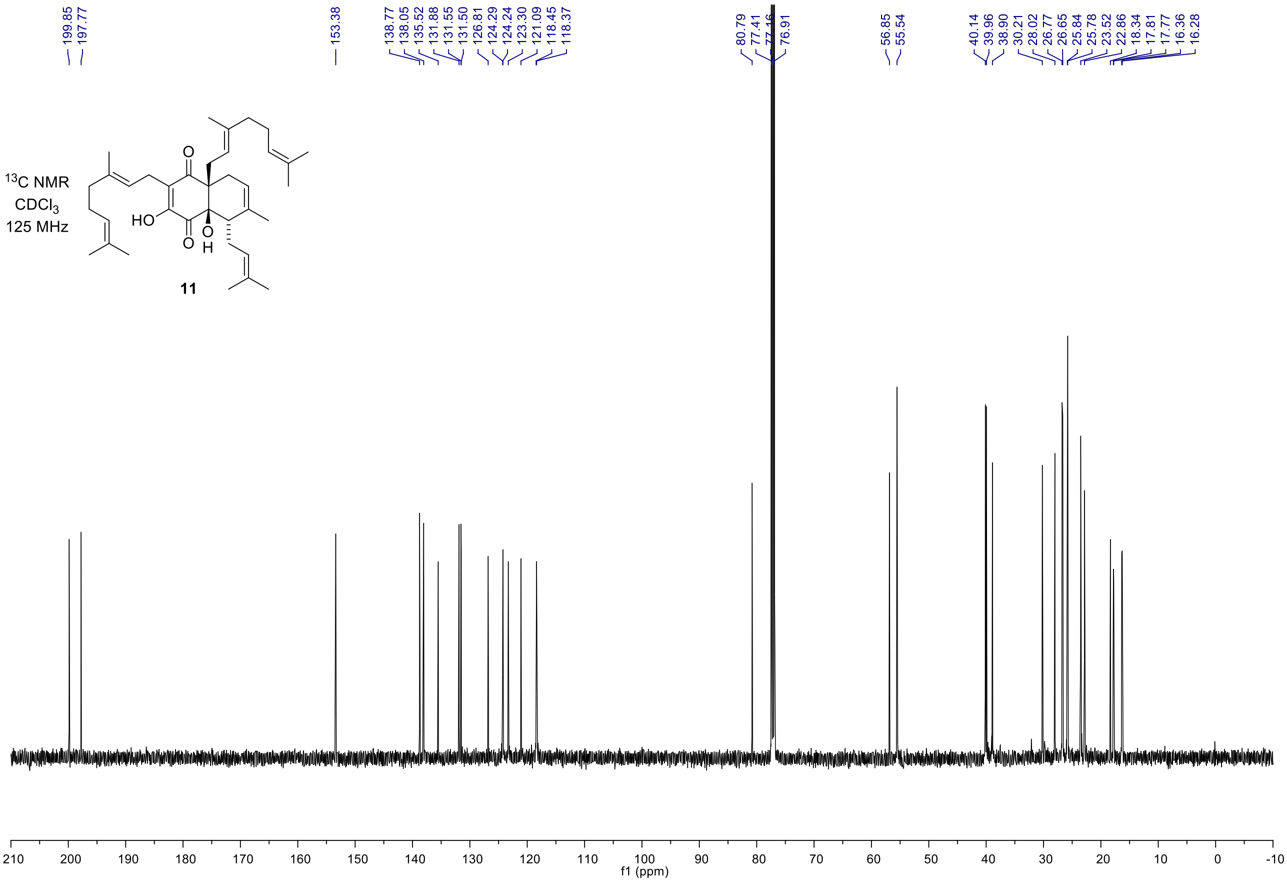

199.85
197.77
153.38
138.77
138.05
135.52
131.88
131.55
131.50
126.81
124.29
124.24
123.30
121.09
118.45
118.37
80.79
77.41
77.16
76.91
56.85
55.54
40.14
39.96
38.90
30.21
28.02
26.77
26.65
25.84
25.78
23.52
22.86
18.34
17.81
17.77
16.36
16.28
^{13}C NMR
$CDCl_3$
125 MHz
HO
O
H
11
f1 (ppm)

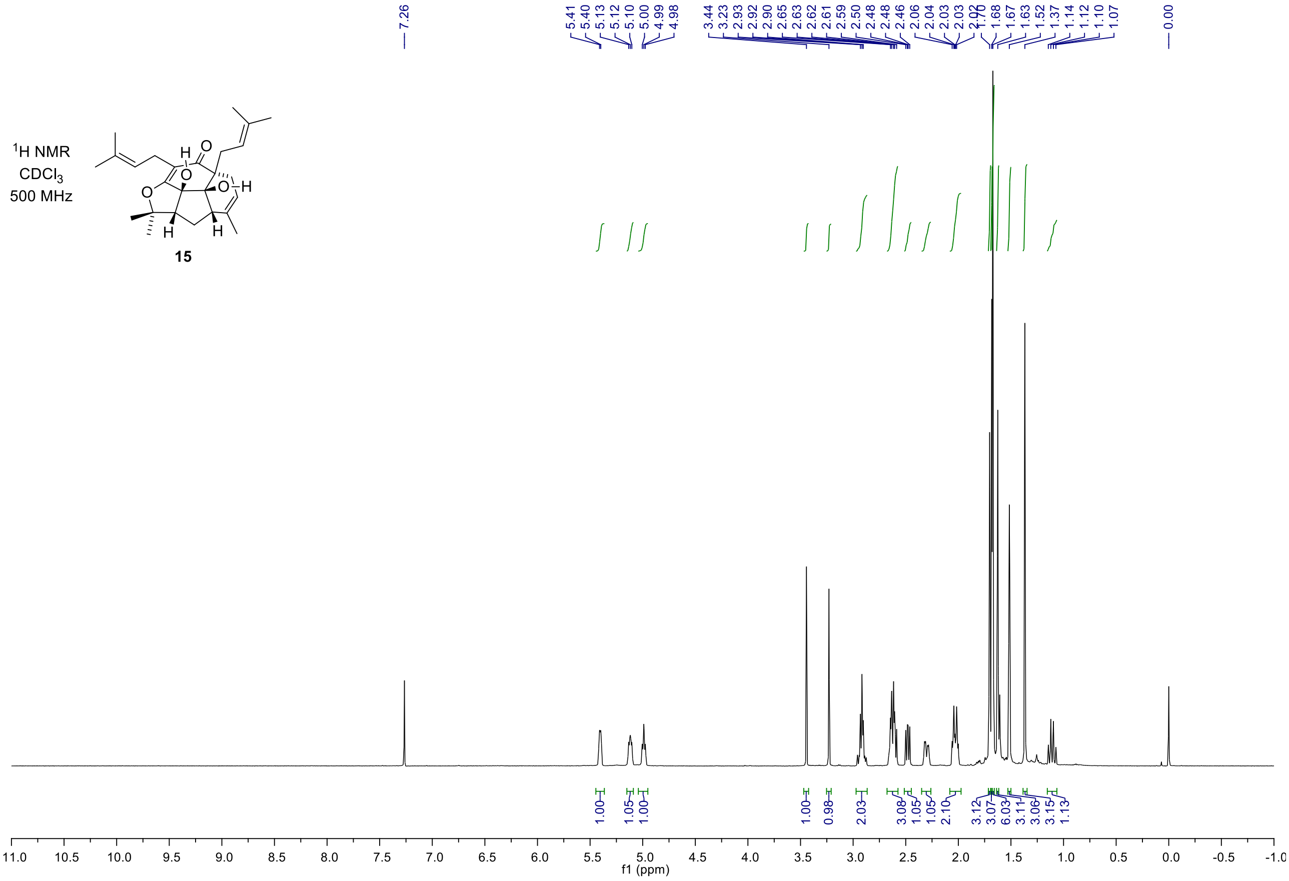

^{1}H NMR
$CDCl_3$
500 MHz
15
f1 (ppm)

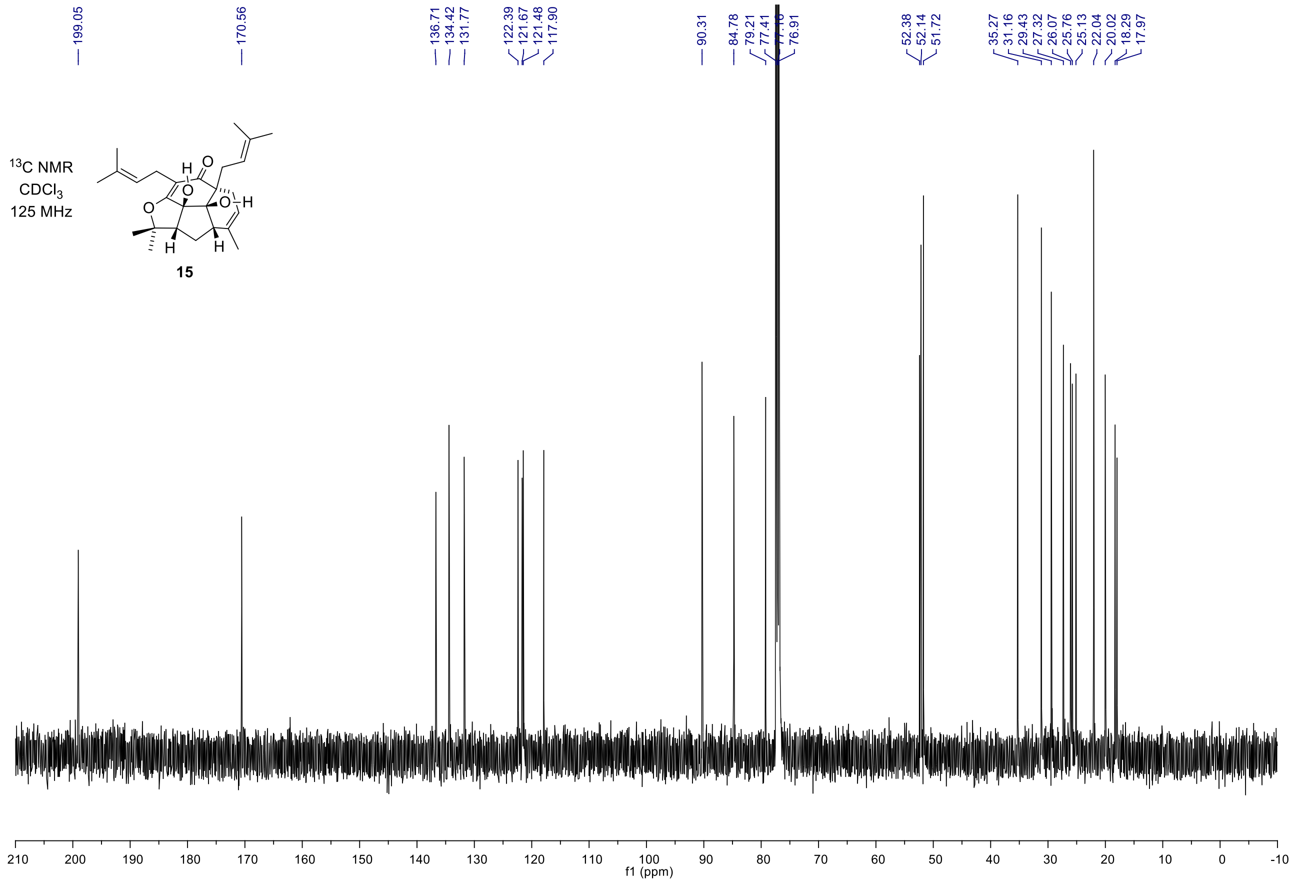

^{13}C NMR
$CDCl_3$
125 MHz
15
f1 (ppm)

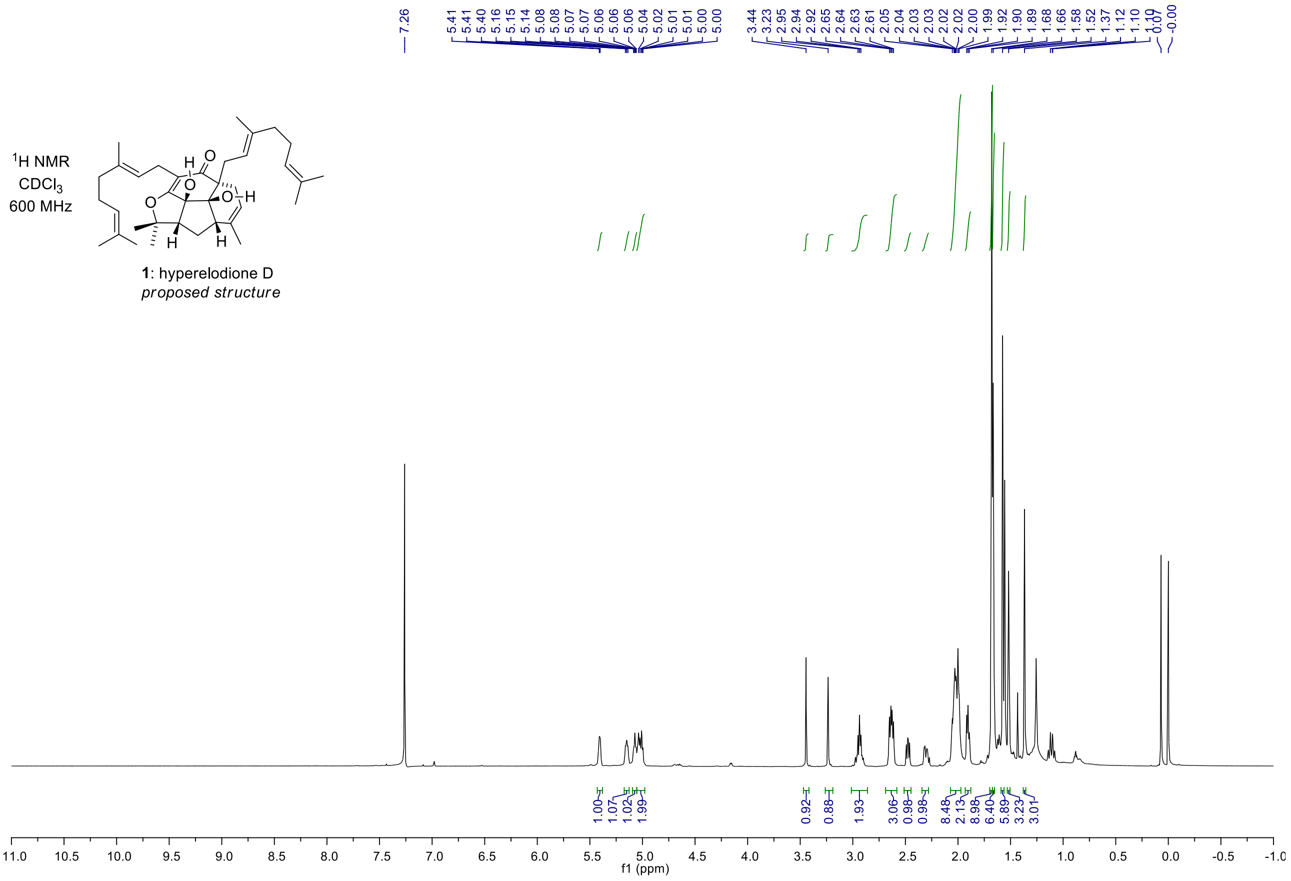

^{1}H NMR
$CDCl_3$
600 MHz
1: hyperelodione D
proposed structure
7.26
5.41 5.41 5.40 5.16 5.15 5.14 5.08 5.07 5.06 5.06 5.06 5.04 5.02 5.01 5.00 5.00
3.44 3.23 2.95 2.94 2.92 2.65 2.64 2.63 2.61 2.05 2.04 2.03 2.03 2.02 2.02 2.00 1.99 1.92 1.90 1.89 1.68 1.66 1.58 1.52 1.37 1.12 1.10 0.07 -0.00
1.00 1.07 1.02 1.99 0.92 0.88 1.93 3.06 0.98 0.98 8.48 2.13 8.98 6.40 5.89 3.23 3.01
f1 (ppm)

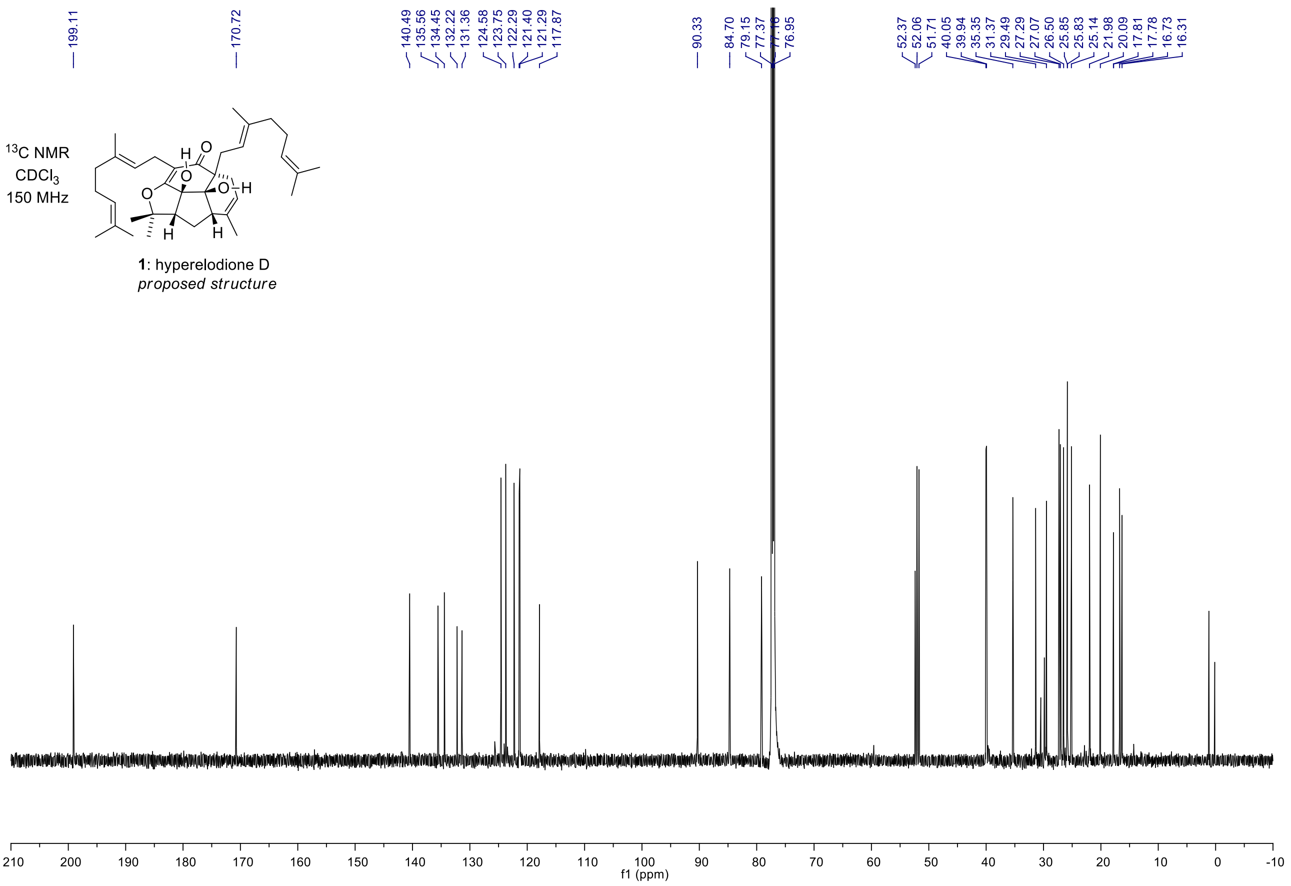

^{13}C NMR
$CDCl_3$
150 MHz
1: hyperelodione D
proposed structure
199.11
170.72
140.49 135.56 134.45 132.22 131.36 124.58 123.75 122.29 121.40 121.29 117.87
90.33
84.70 79.15 77.37 77.16 76.95
52.37 52.06 51.71 40.05 39.94 35.35 31.37 29.49 27.29 27.07 26.50 25.85 25.83 25.14 21.98 20.09 17.81 17.78 16.73 16.31
f1 (ppm)

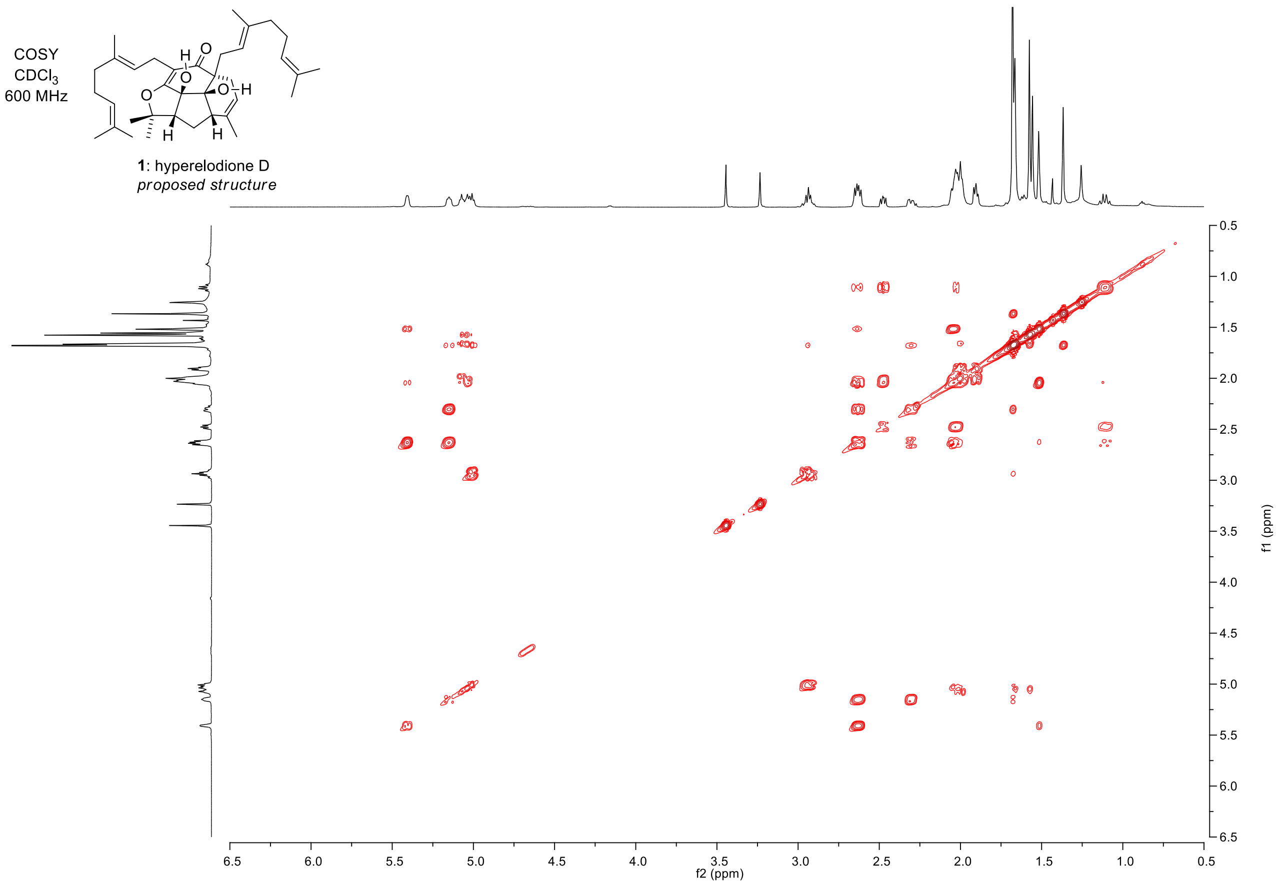
COSY
$CDCl_3$
600 MHz
1: hyperelodione D
proposed structure
f2 (ppm)
f1 (ppm)

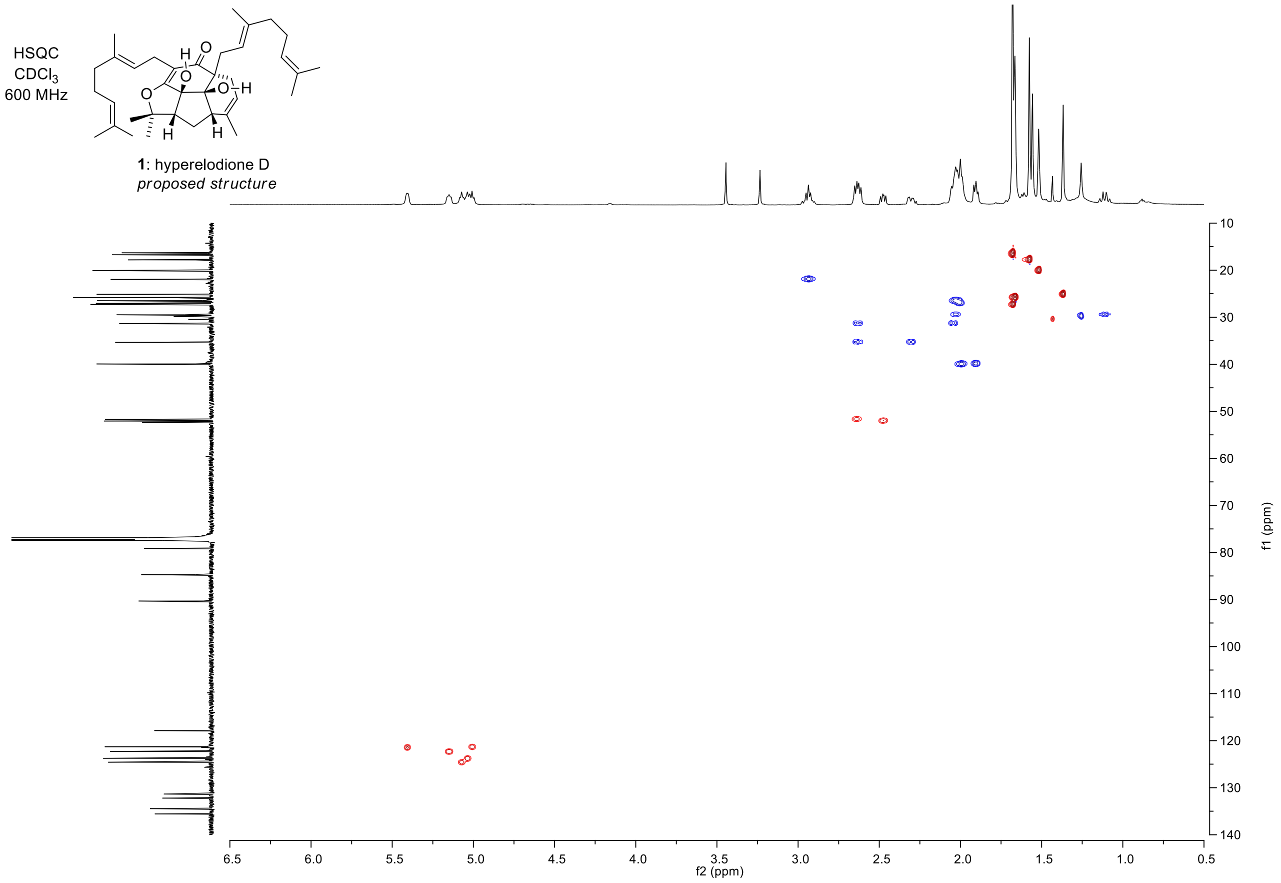
HSQC
$CDCl_3$
600 MHz
1: hyperelodione D
proposed structure
f2 (ppm)
f1 (ppm)

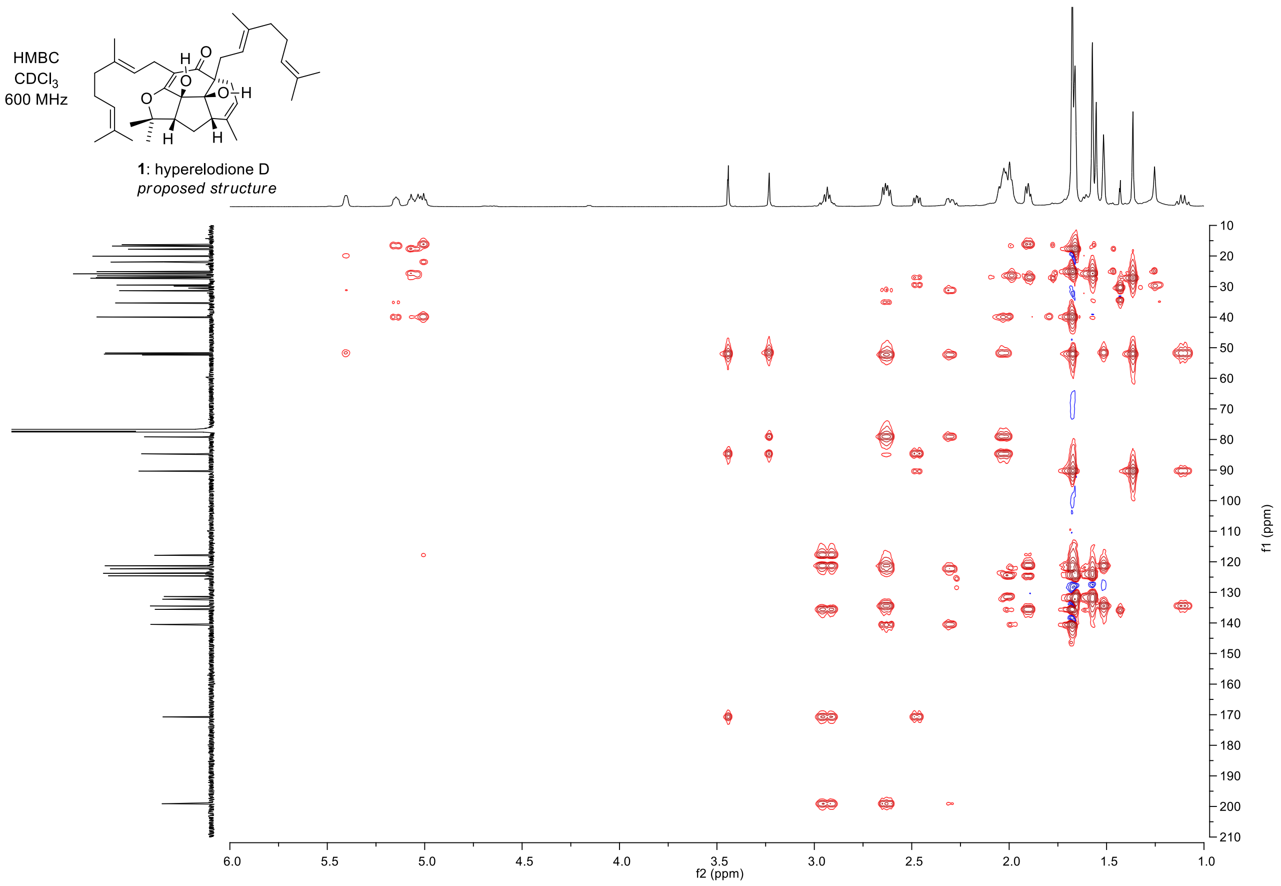
HMBC
CDCl3
600 MHz
1: hyperelodione D
proposed structure
f2 (ppm)
f1 (ppm)

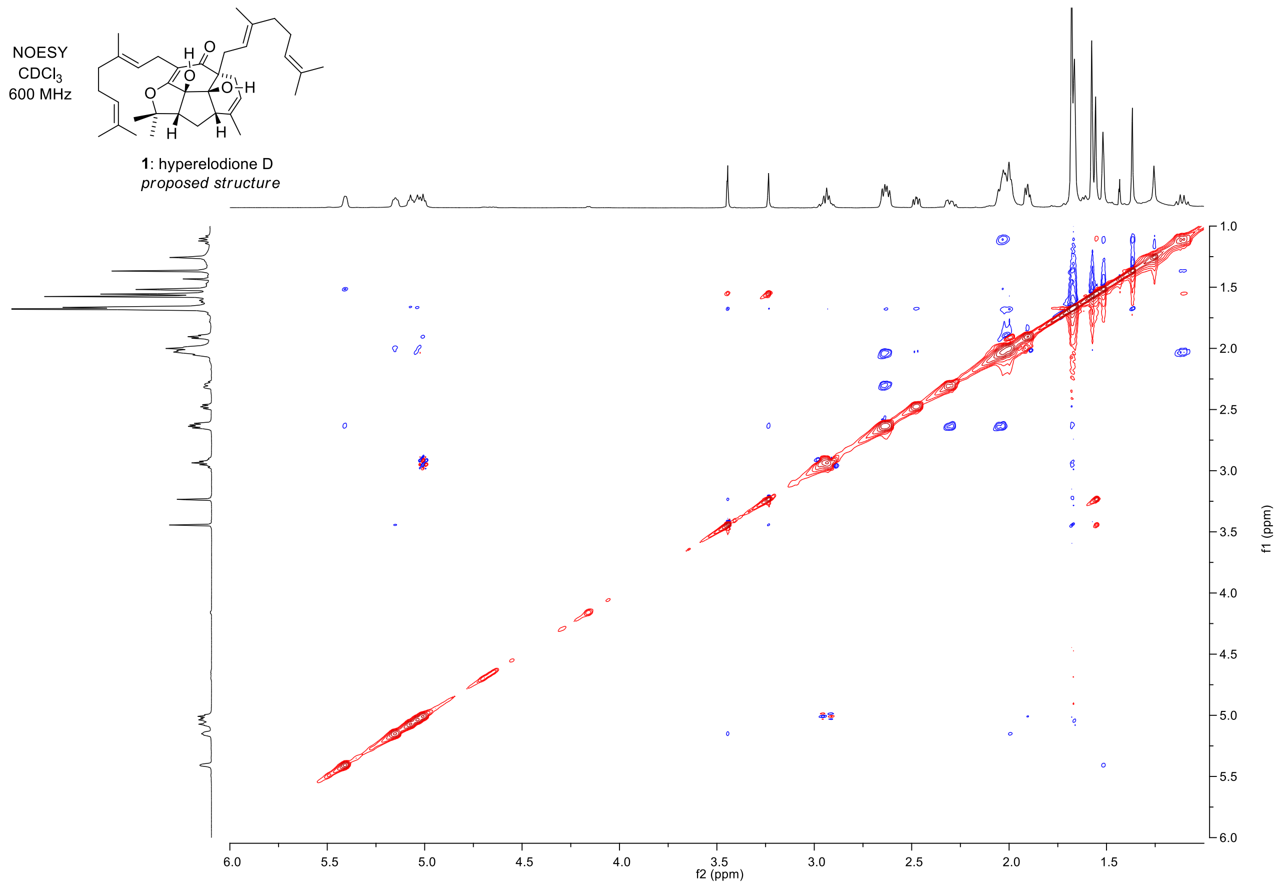
NOESY
CDCl3
600 MHz
1: hyperelodione D
proposed structure
f2 (ppm)
f1 (ppm)

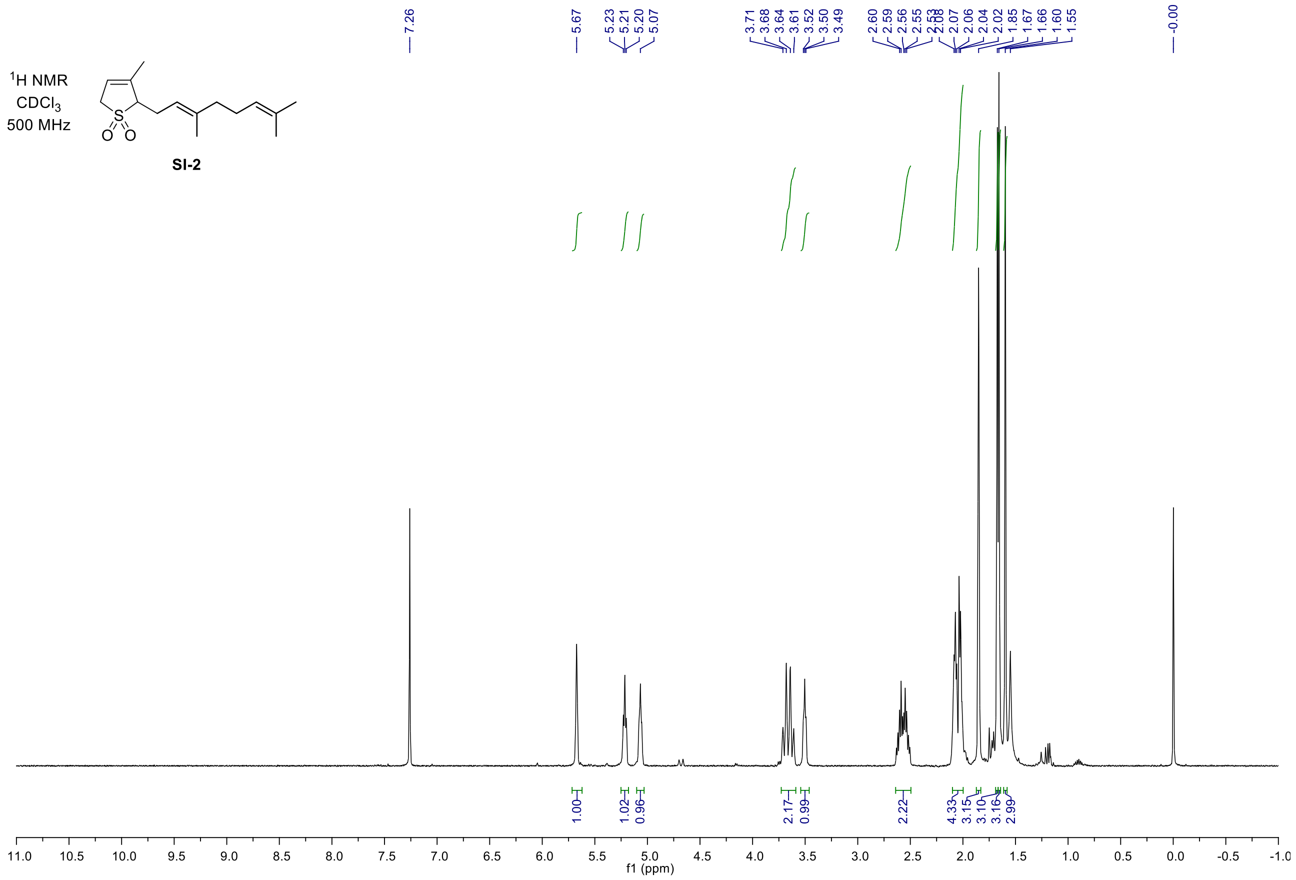

1H NMR
CDCl3
500 MHz
SI-2
f1 (ppm)

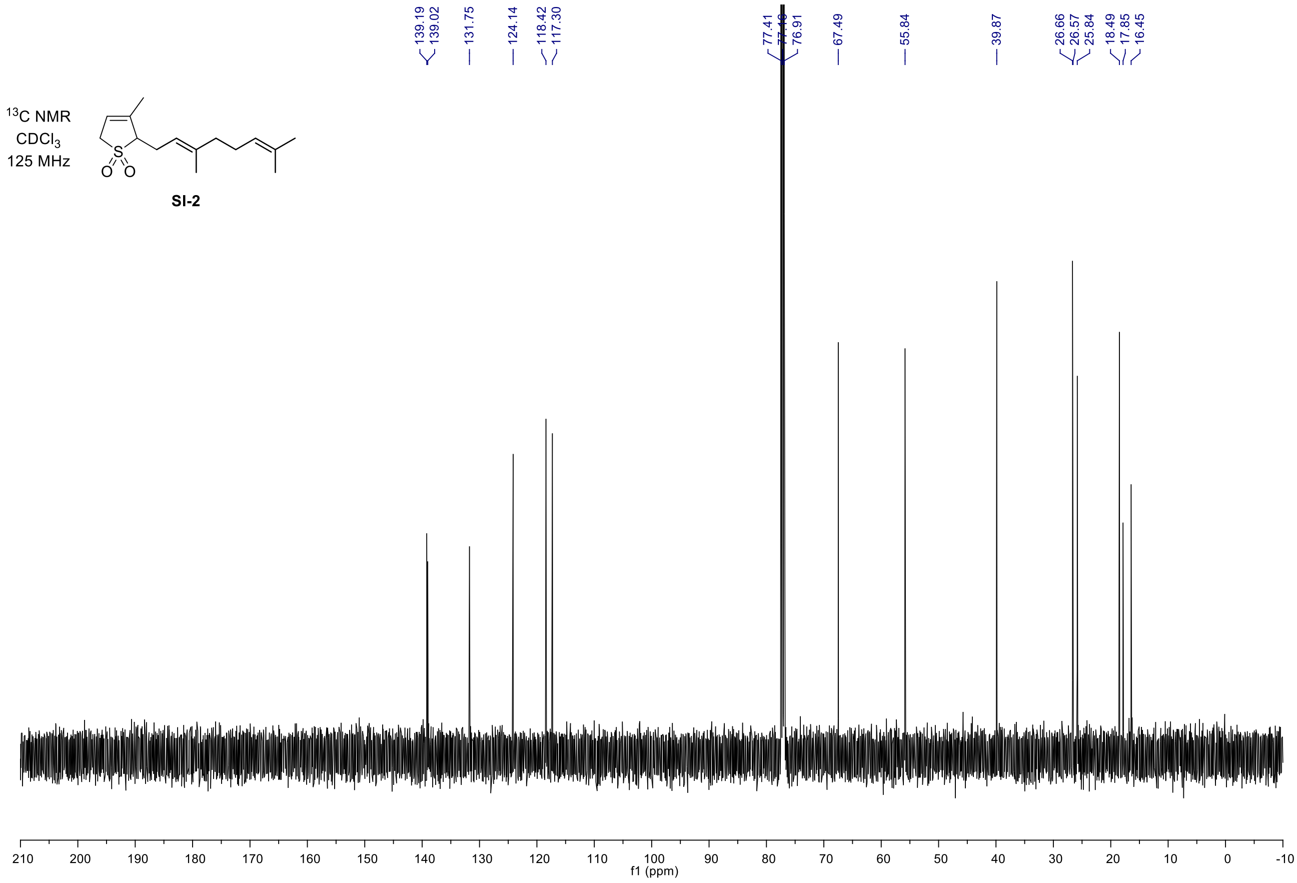

13C NMR
CDCl3
125 MHz
SI-2
f1 (ppm)

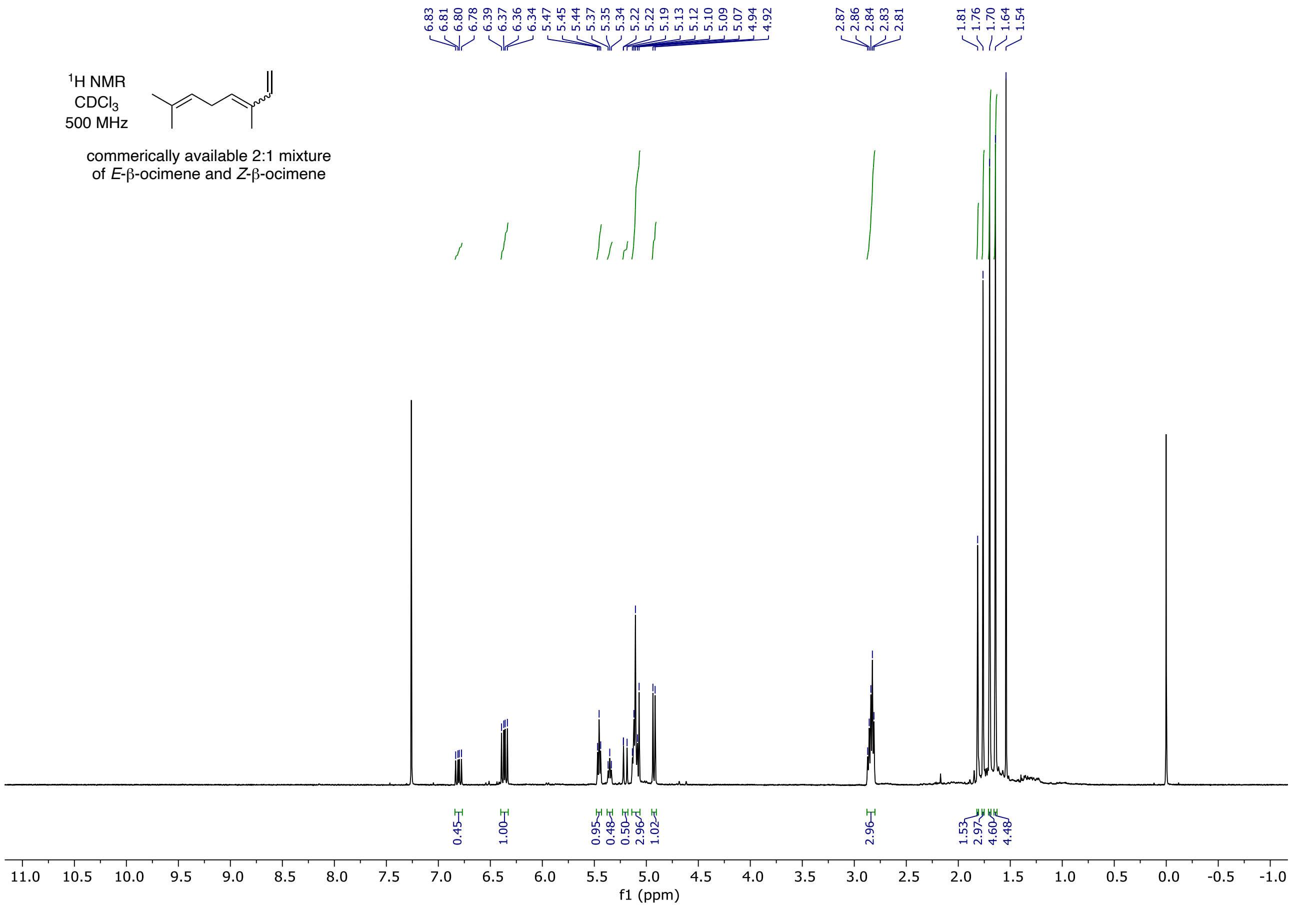

^{1}H NMR
$CDCl_3$
500 MHz
commerically available 2:1 mixture
of *E*-β-ocimene and *Z*-β-ocimene
f1 (ppm)

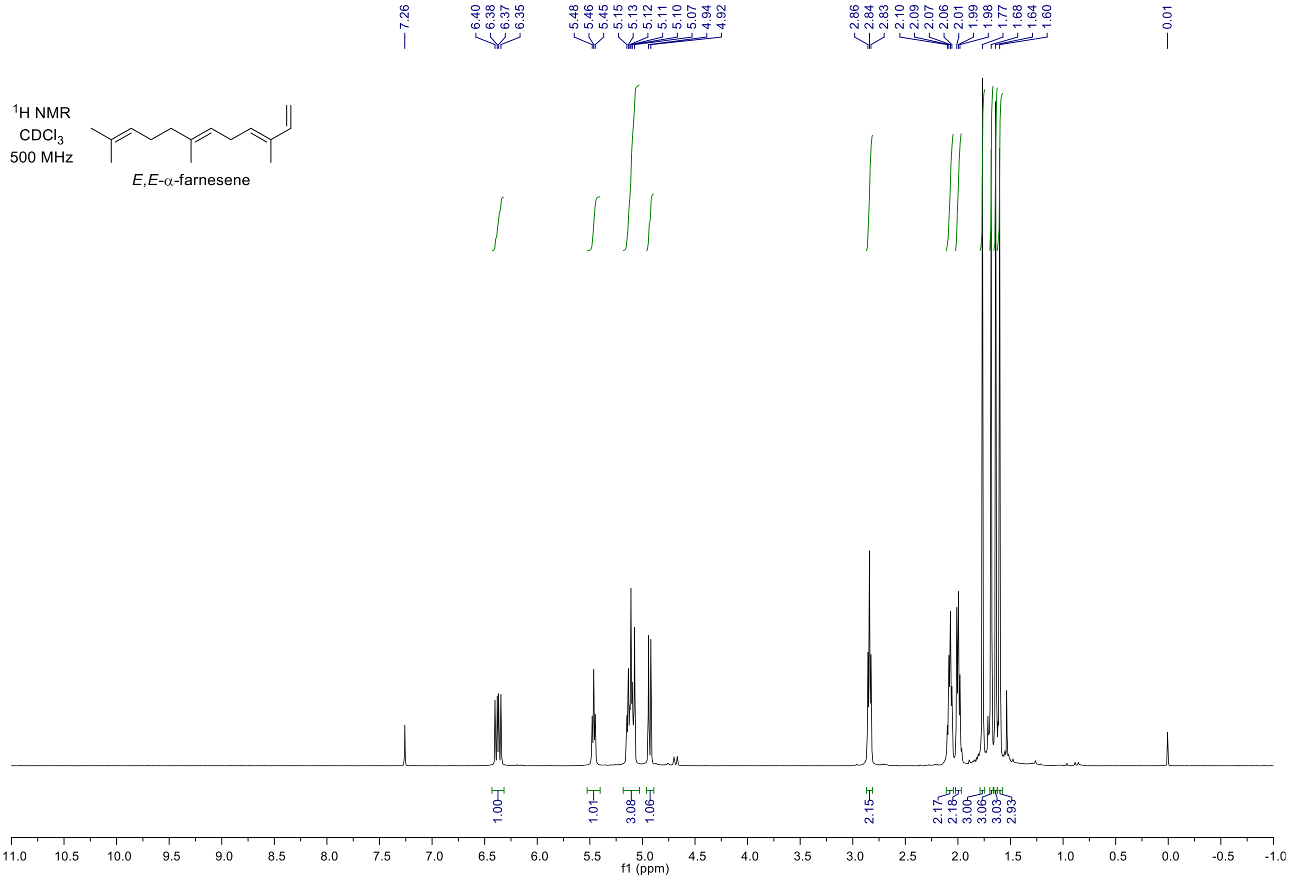
7.26
6.40 6.38 6.37 6.35
5.48 5.46 5.45 5.15 5.13 5.12 5.11 5.10 5.07 4.94 4.92
2.86 2.84 2.83 2.10 2.09 2.07 2.06 2.01 1.99 1.98 1.77 1.68 1.64 1.60
0.01
1H NMR
CDCl3
500 MHz
E,E-α-farnesene
1.00 1.01 3.08 1.06 2.15 2.17 2.18 3.00 3.06 3.03 2.93
f1 (ppm)

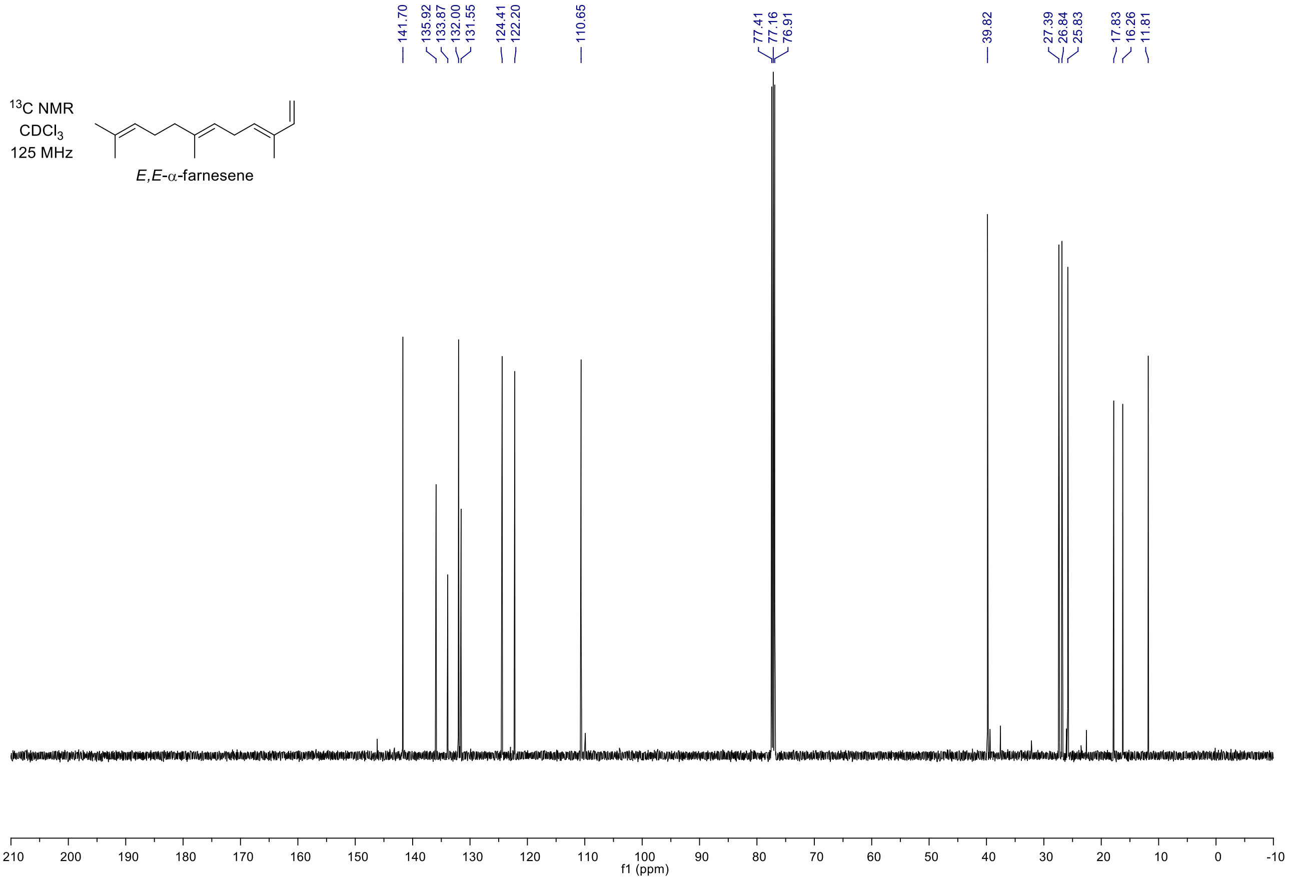
141.70 135.92 133.87 132.00 131.55 124.41 122.20
110.65
77.41 77.16 76.91
39.82
27.39 26.84 25.83 17.83 16.26 11.81
13C NMR
CDCl3
125 MHz
E,E-α-farnesene
f1 (ppm)

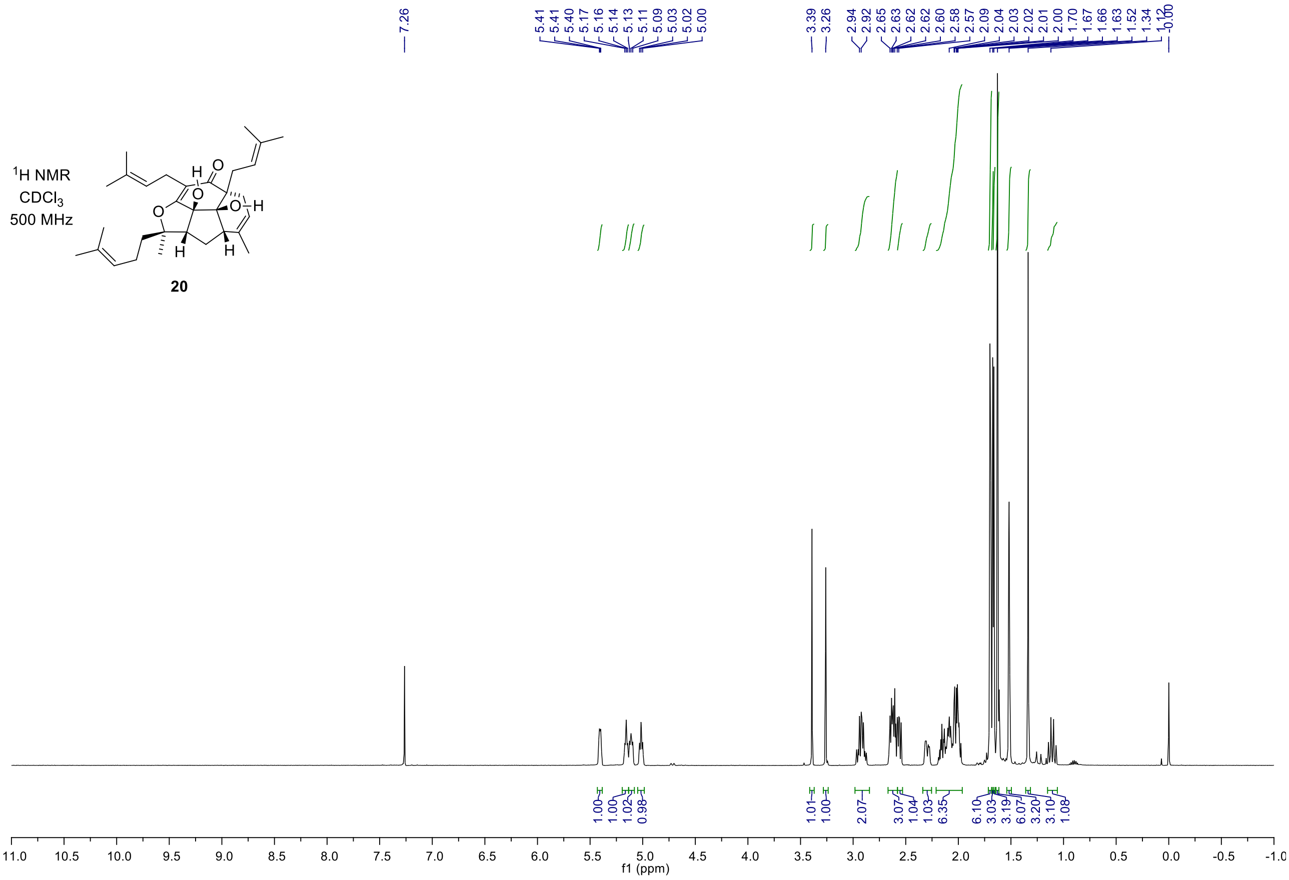

^{1}H NMR
$CDCl_3$
500 MHz
20
7.26
5.41 5.41 5.40 5.17 5.16 5.14 5.13 5.11 5.09 5.03 5.02 5.00
3.39 3.26 2.94 2.92 2.65 2.63 2.62 2.62 2.60 2.58 2.57 2.09 2.04 2.03 2.02 2.01 2.00 1.70 1.67 1.66 1.63 1.52 1.34 1.12 0.00
1.00 1.00 1.02 0.98 1.01 1.00 2.07 3.07 1.04 1.03 6.35 6.10 3.03 3.19 6.07 3.20 3.10 1.08
f1 (ppm)

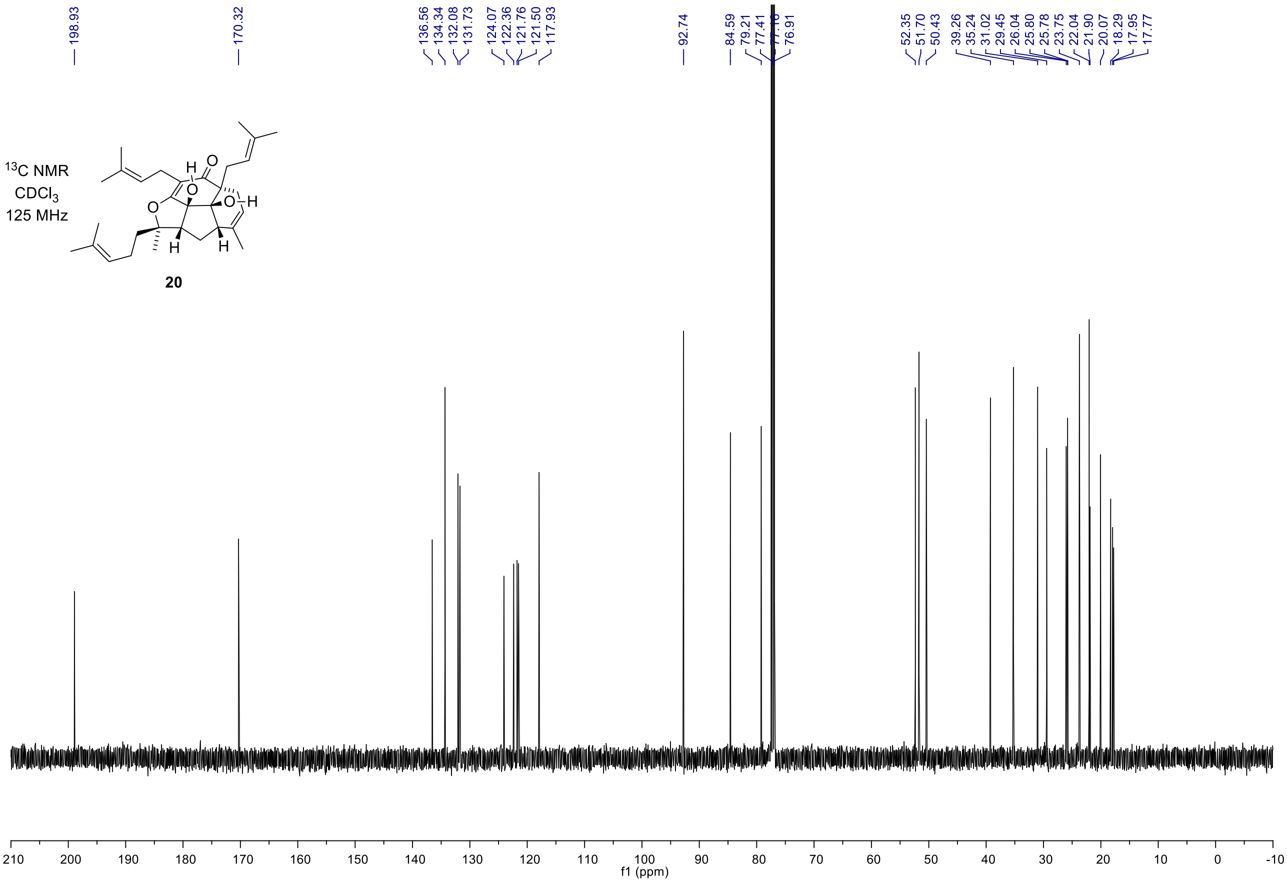

^{13}C NMR
$CDCl_3$
125 MHz
20
198.93
170.32
136.56 134.34 132.08 131.73 124.07 122.36 121.76 121.50 117.93
92.74
84.59 79.21 77.41 76.91
52.35 51.70 50.43 39.26 35.24 31.02 29.45 26.04 25.80 25.78 23.75 22.04 21.90 20.07 18.29 17.95 17.77
f1 (ppm)

COSY
$CDCl_3$
500 MHz

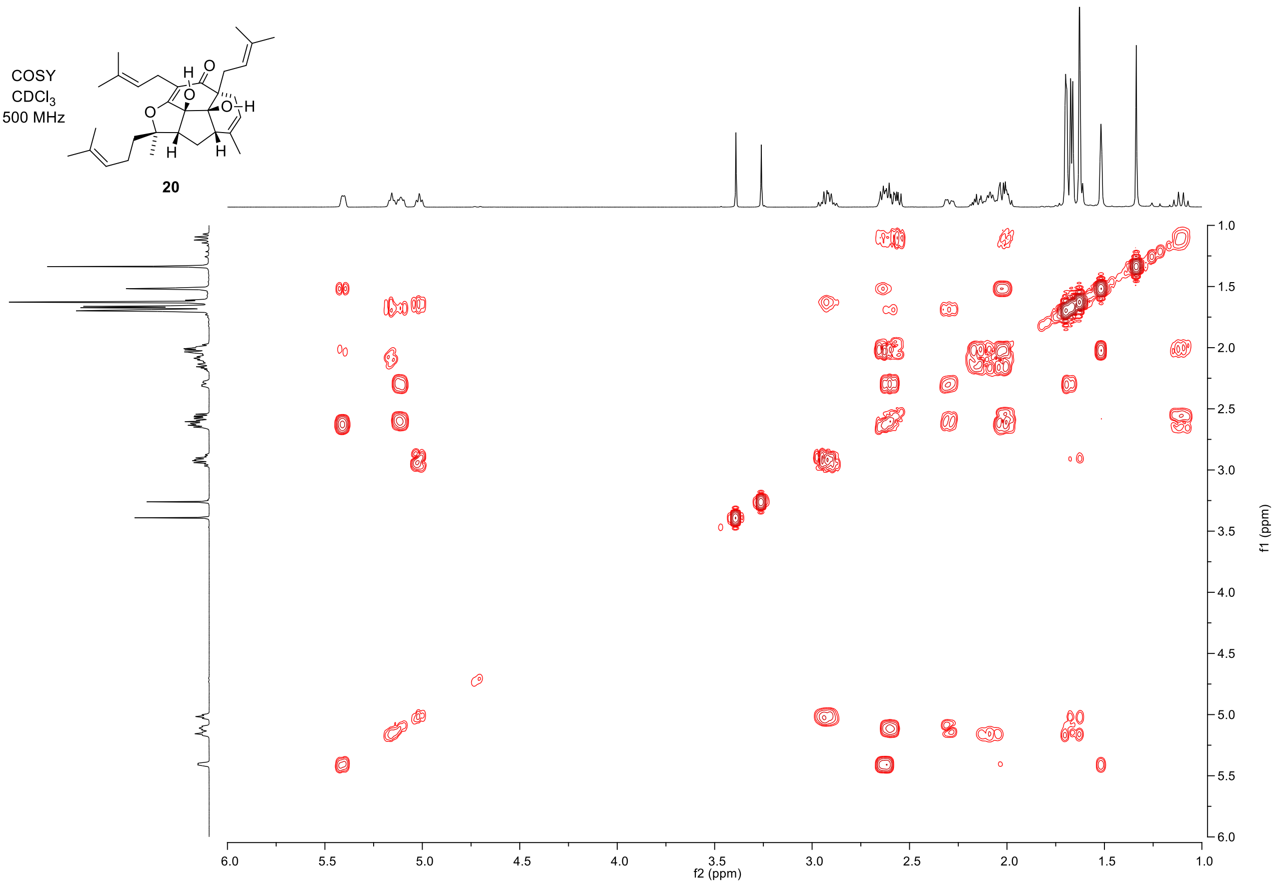

HSQC
$CDCl_3$
600 MHz

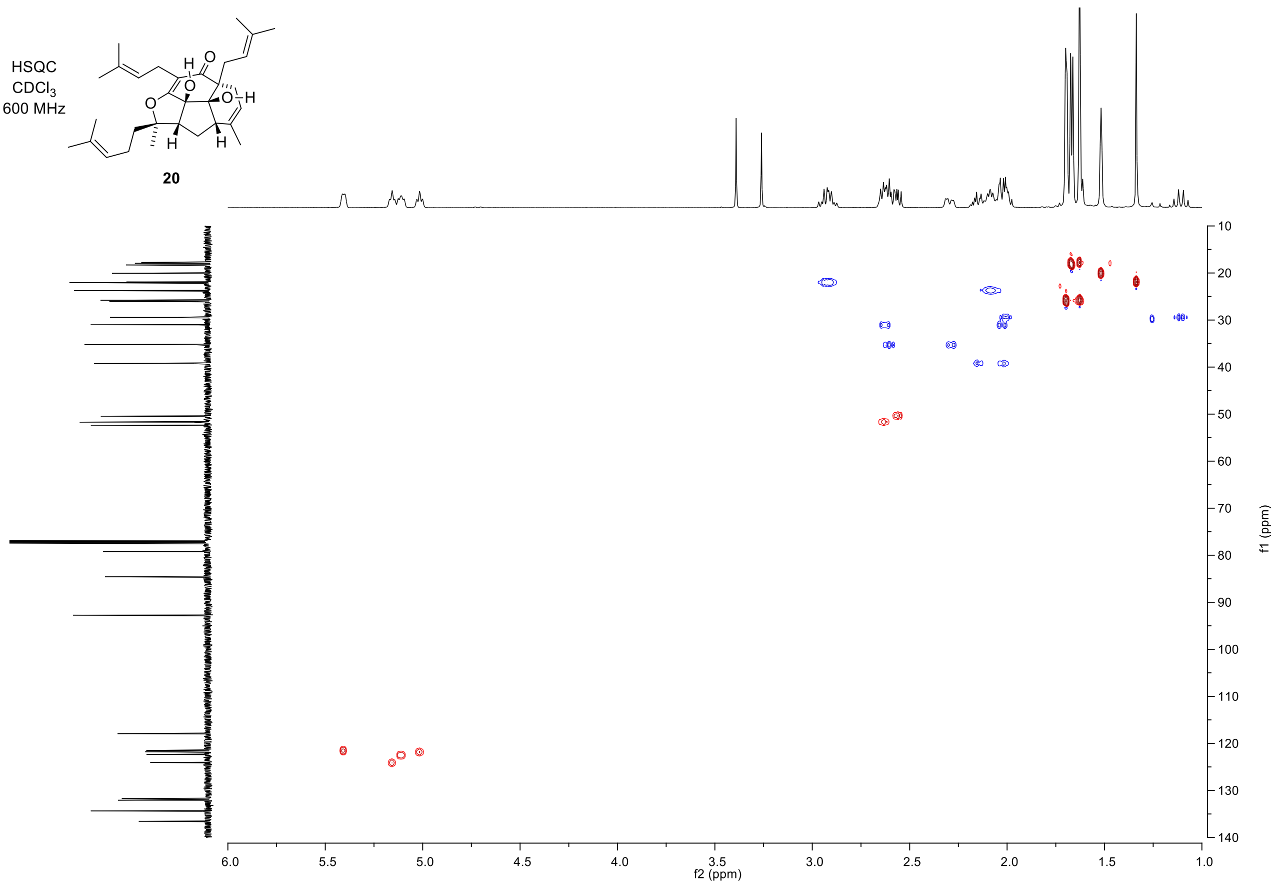

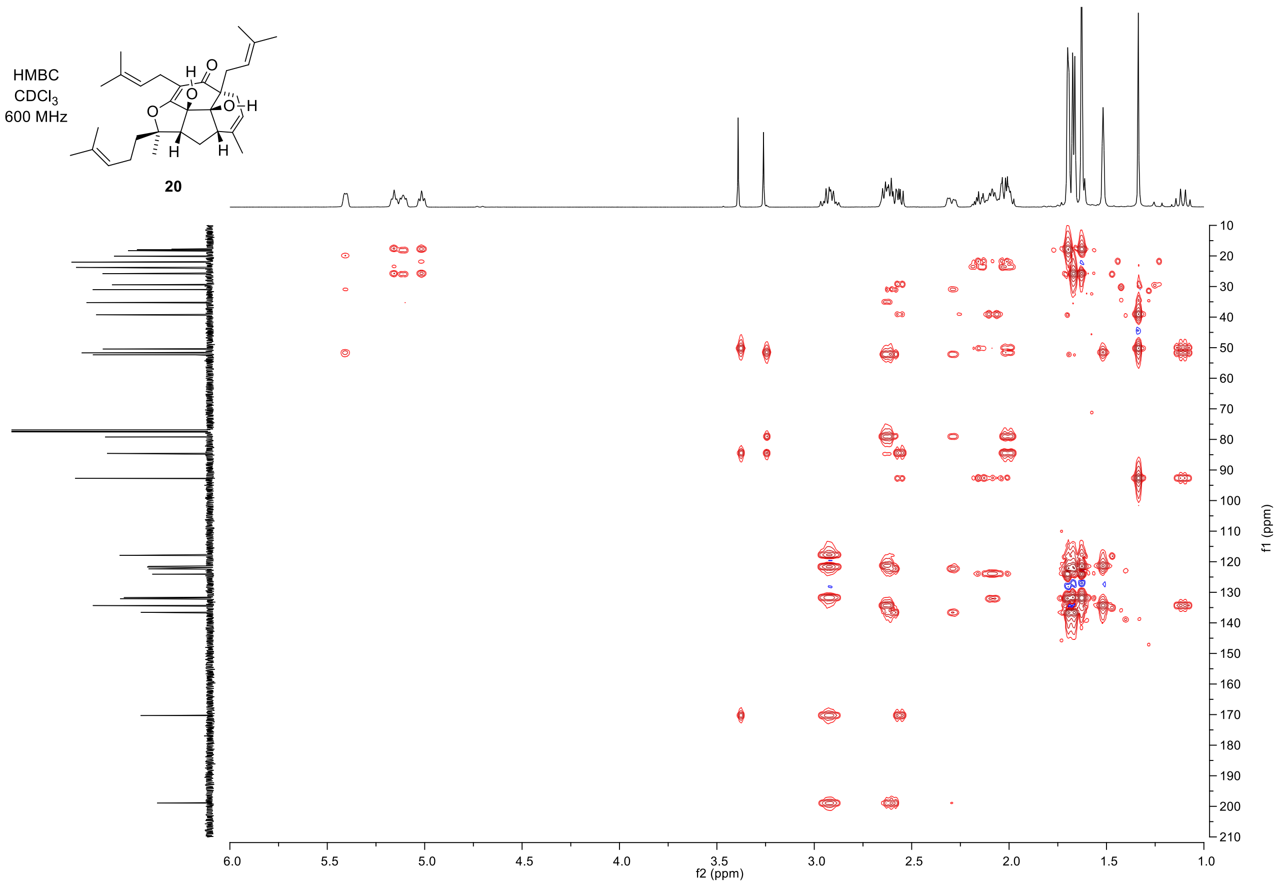
HMBC
CDCl3
600 MHz
20
f2 (ppm)
f1 (ppm)

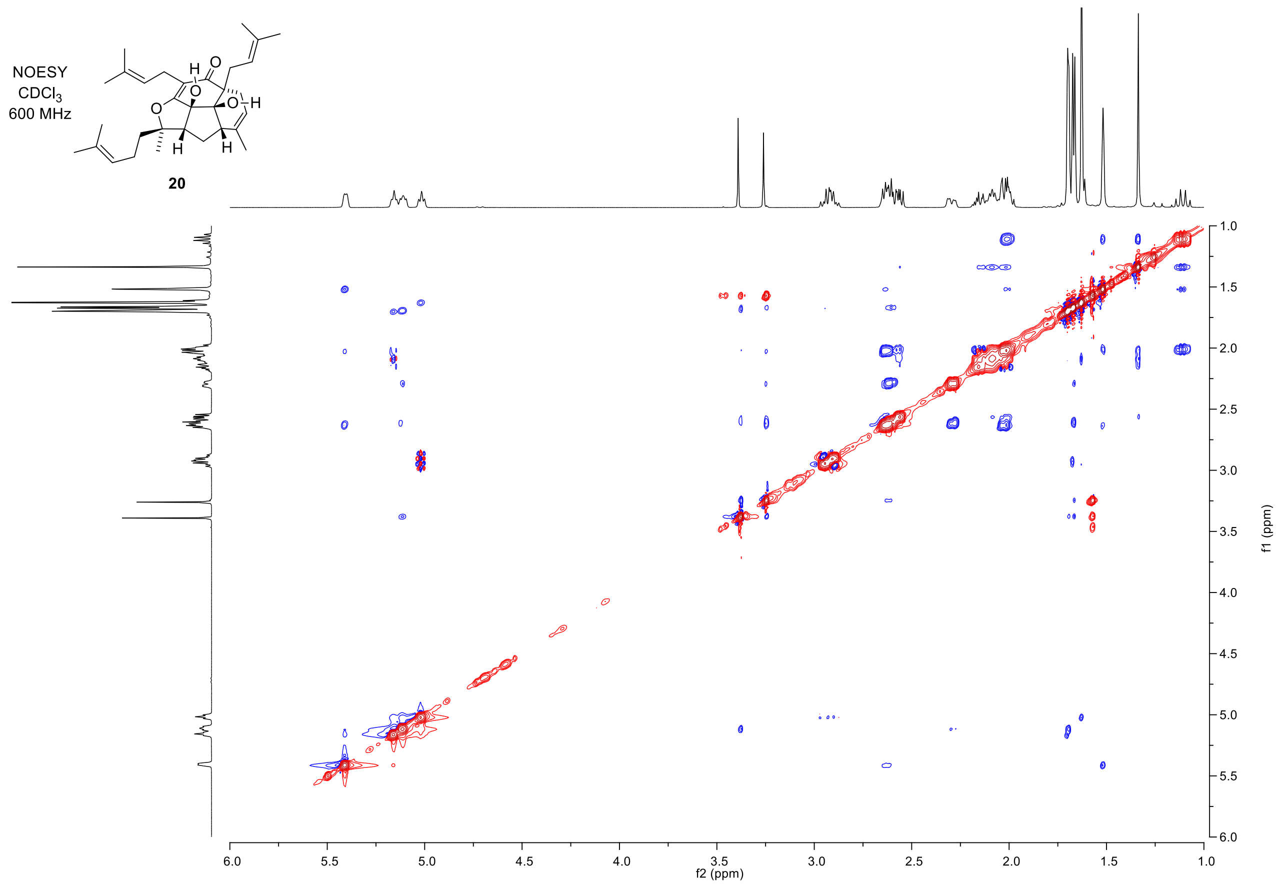
NOESY
CDCl3
600 MHz
20
f2 (ppm)
f1 (ppm)

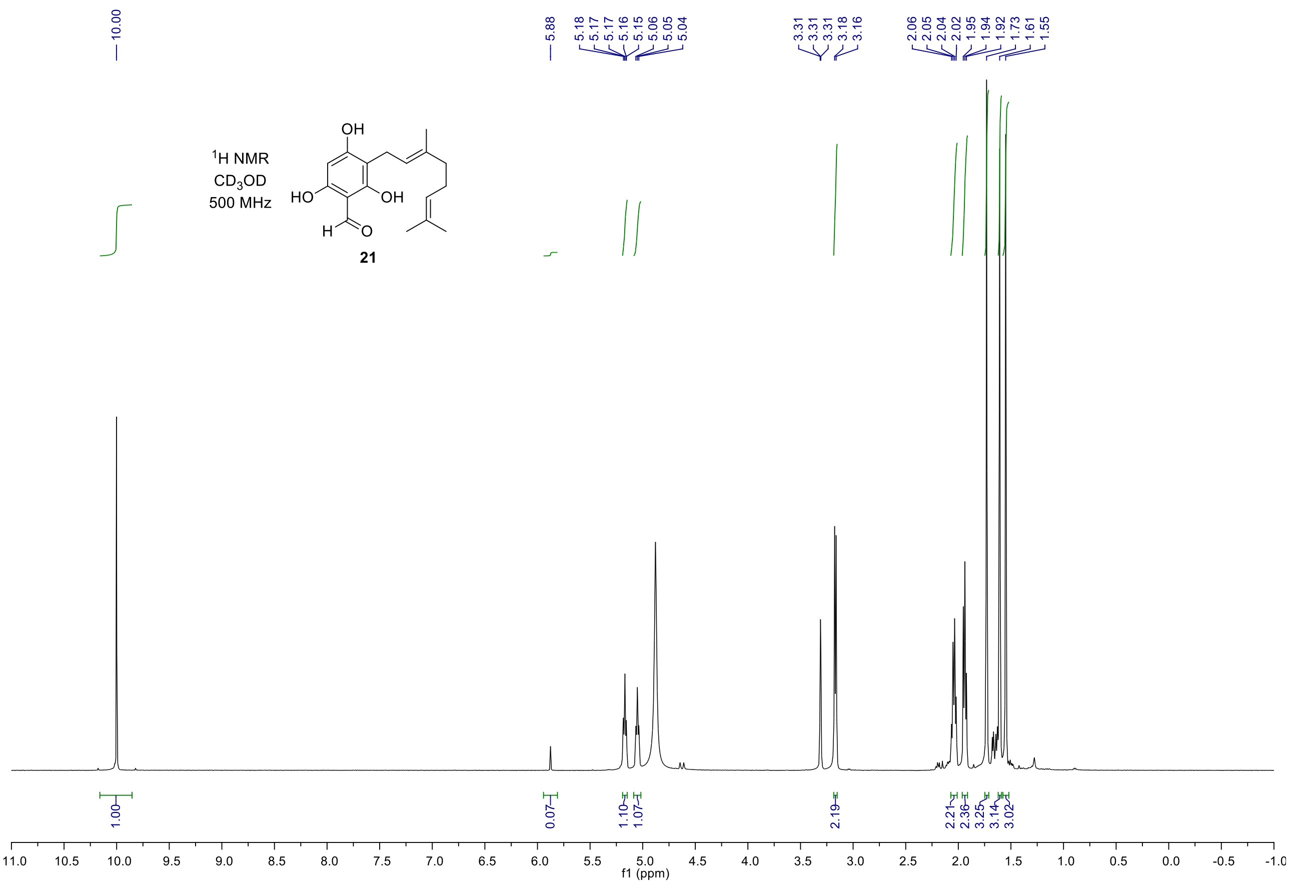

^{1}H NMR
CD_3OD
500 MHz
21
10.00
5.88
5.18 5.17 5.17 5.16 5.15 5.06 5.05 5.04
3.31 3.31 3.31 3.18 3.16
2.06 2.05 2.04 2.02 1.95 1.94 1.92 1.73 1.61 1.55
1.00 0.07 1.10 1.07 2.19 2.21 2.36 3.25 3.14 3.02
f1 (ppm)

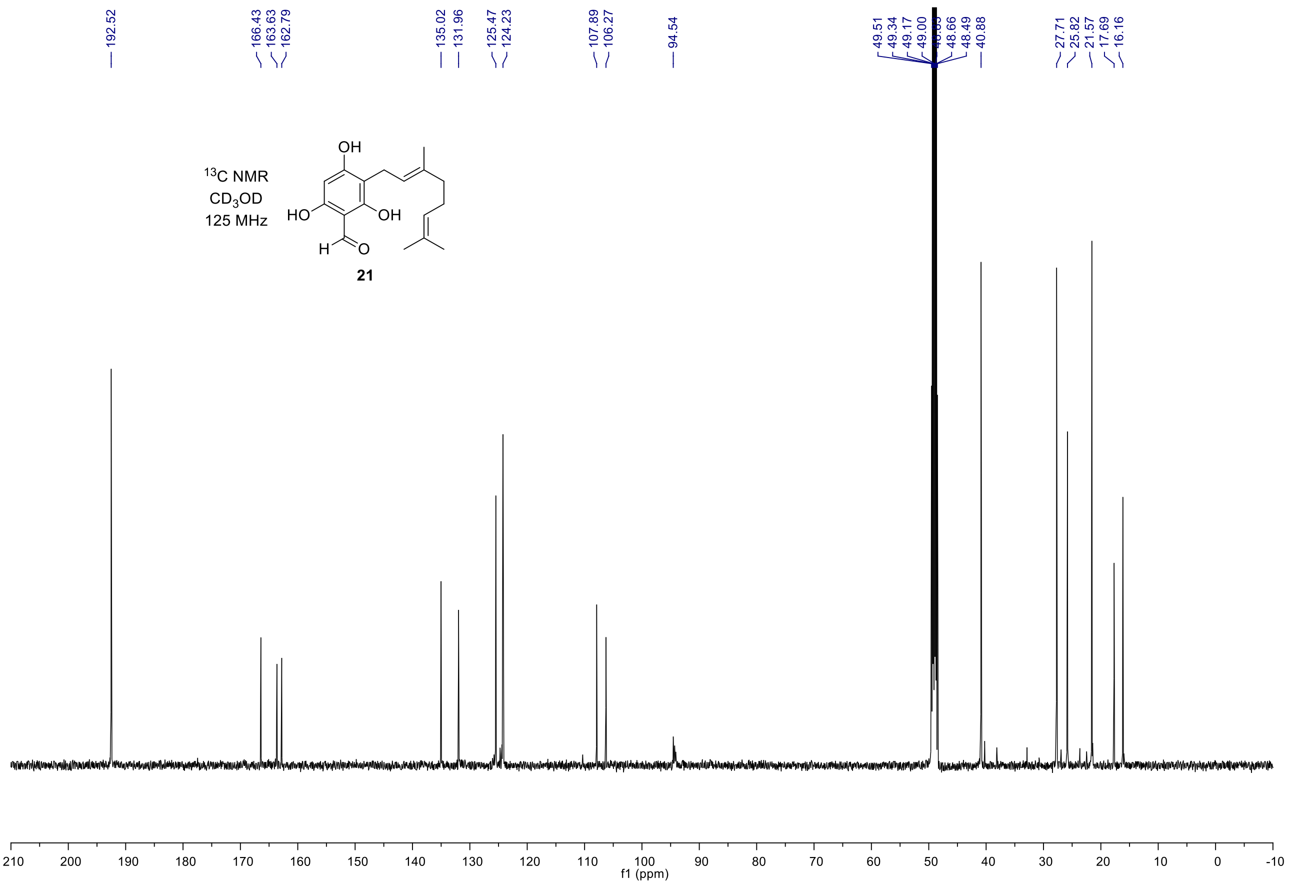

^{13}C NMR
CD_3OD
125 MHz
21
192.52
166.43 163.63 162.79
135.02 131.96 125.47 124.23
107.89 106.27
94.54
49.51 49.34 49.17 49.00 48.66 48.49 40.88
27.71 25.82 21.57 17.69 16.16
f1 (ppm)

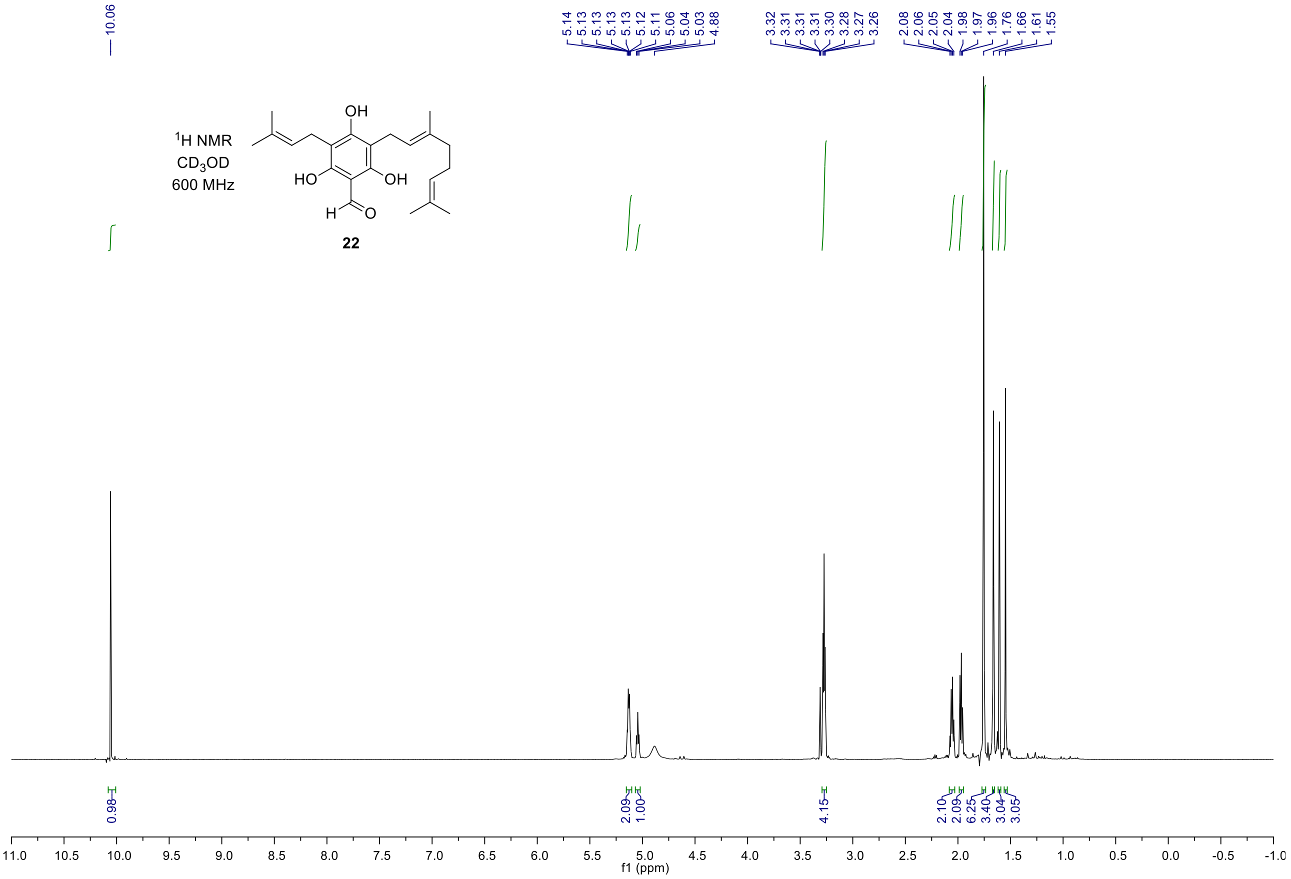
10.06
5.14 5.13 5.13 5.13 5.13 5.12 5.11 5.06 5.04 5.03 4.88
3.32 3.31 3.31 3.31 3.30 3.28 3.27 3.26
2.08 2.06 2.05 2.04 1.98 1.97 1.96 1.76 1.66 1.61 1.55
1H NMR
CD3OD
600 MHz
OH
HO
OH
H
O
22
0.98
2.09
1.00
4.15
2.10
2.09
6.25
3.40
3.04
3.05
11.0 10.5 10.0 9.5 9.0 8.5 8.0 7.5 7.0 6.5 6.0 5.5 5.0 4.5 4.0 3.5 3.0 2.5 2.0 1.5 1.0 0.5 0.0 -0.5 -1.0
f1 (ppm)

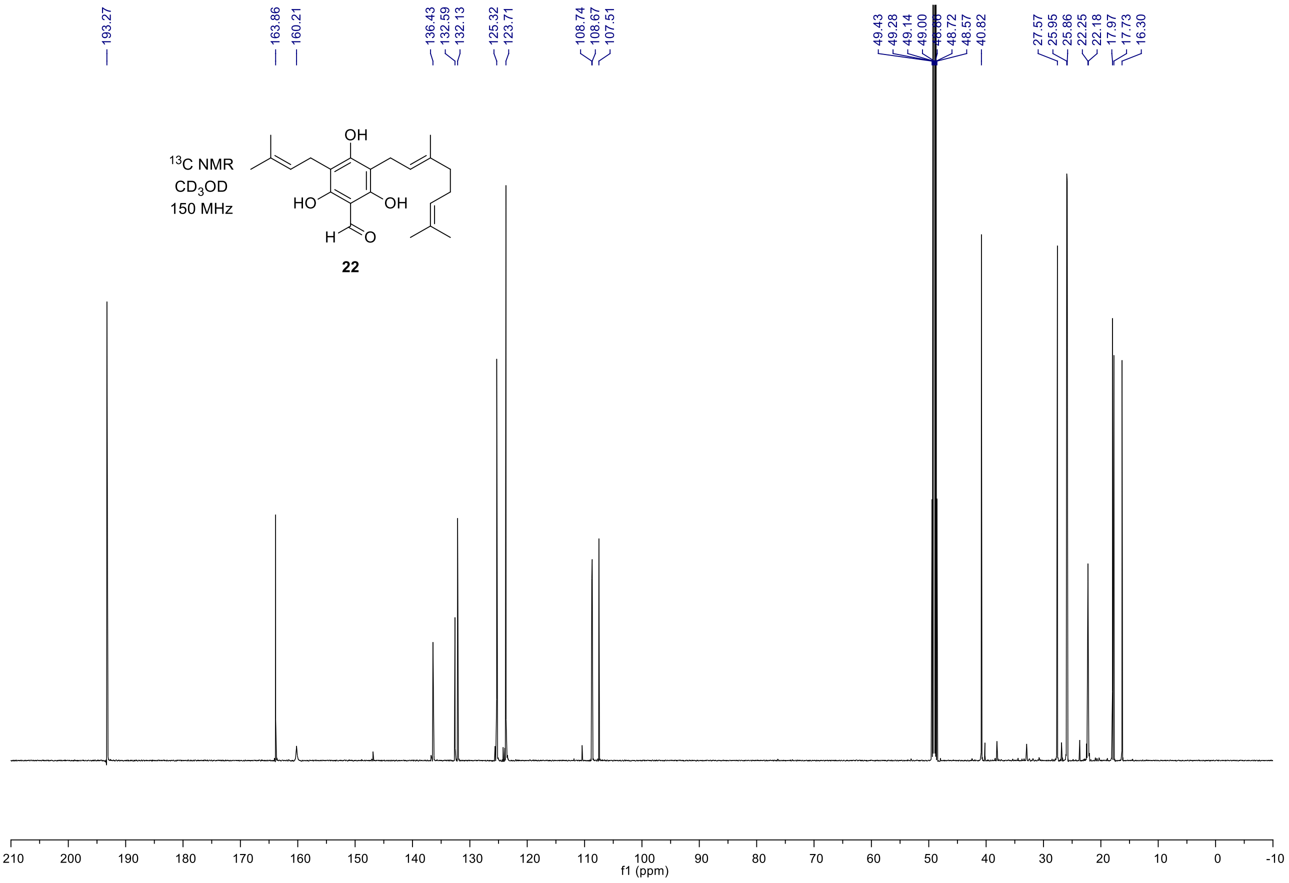
193.27
163.86
160.21
136.43 132.59 132.13
125.32 123.71
108.74 108.67 107.51
49.43 49.28 49.14 49.00 48.86 48.72 48.57 40.82
27.57 25.95 25.86 22.25 22.18 17.97 17.73 16.30
13C NMR
CD3OD
150 MHz
OH
HO
OH
H
O
22
210 200 190 180 170 160 150 140 130 120 110 100 90 80 70 60 50 40 30 20 10 0 -10
f1 (ppm)

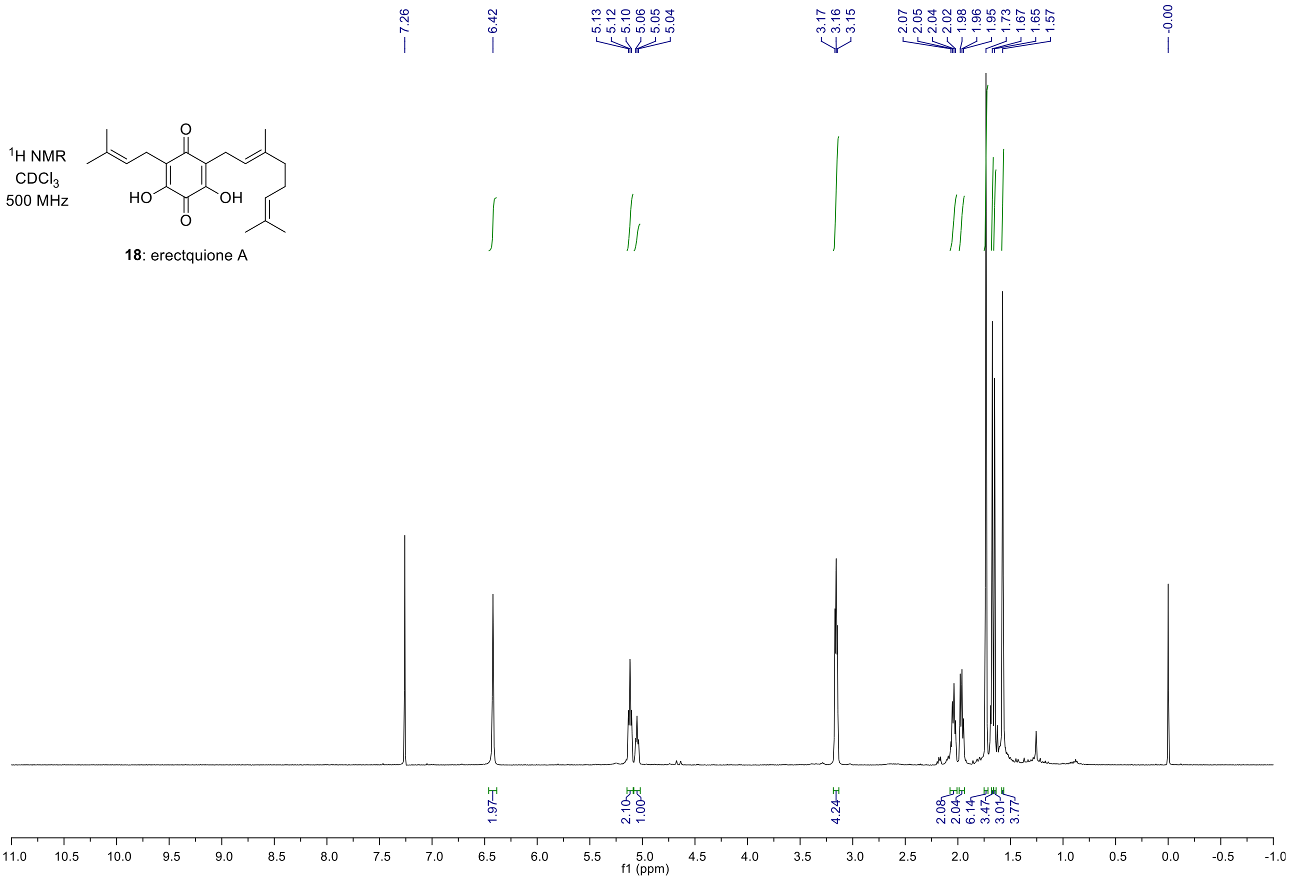
7.26
6.42
5.13
5.12
5.10
5.06
5.05
5.04
3.17
3.16
3.15
2.07
2.05
2.04
2.02
1.98
1.96
1.95
1.73
1.67
1.65
1.57
-0.00
¹H NMR
CDCl₃
500 MHz
HO
OH
O
O
18: erectquione A
1.97
2.10
1.00
4.24
2.08
2.04
6.14
3.47
3.01
3.77
f1 (ppm)

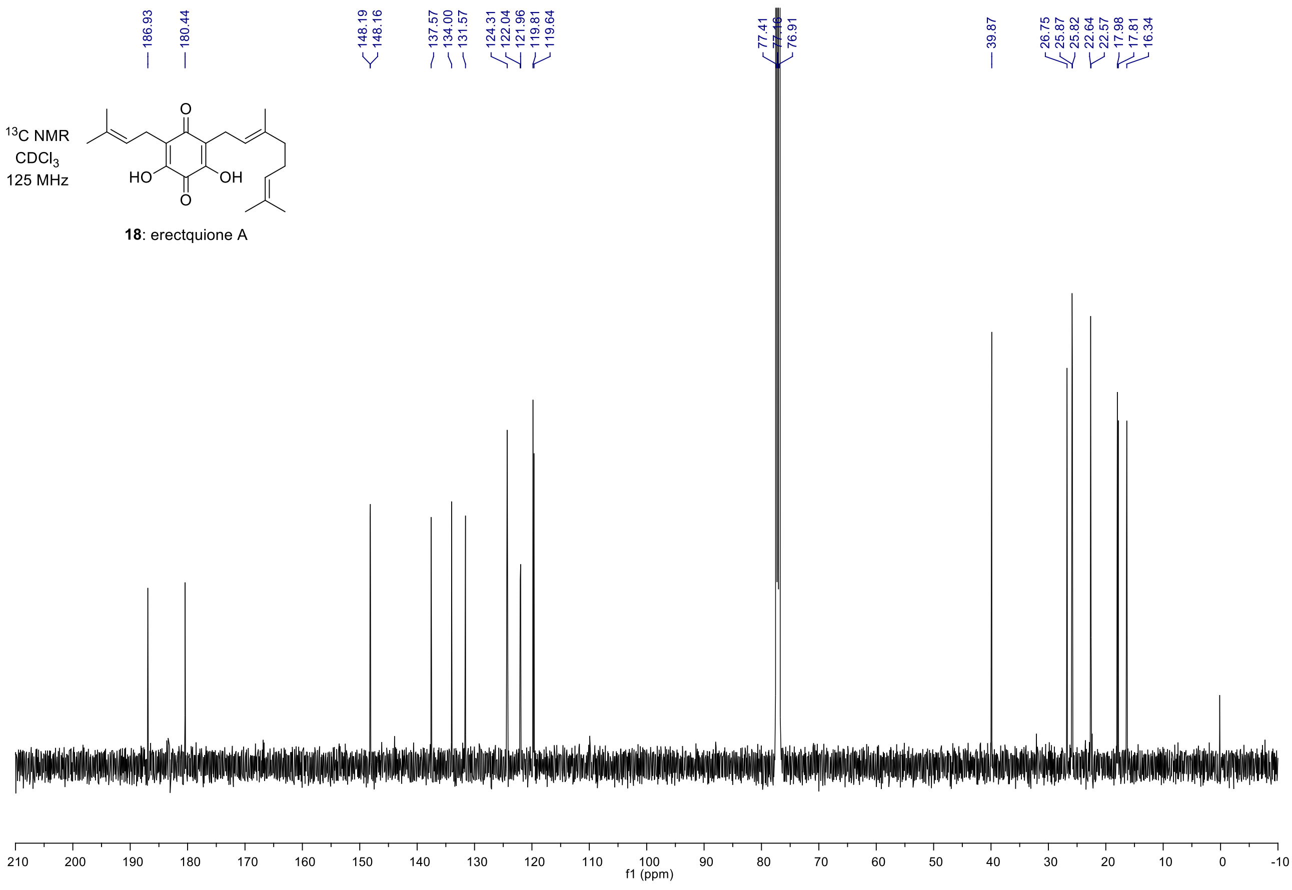
186.93
180.44
148.19
148.16
137.57
134.00
131.57
124.31
122.04
121.96
119.81
119.64
77.41
77.16
76.91
39.87
26.75
25.87
25.82
22.64
22.57
17.98
17.81
16.34
¹³C NMR
CDCl₃
125 MHz
HO
OH
O
O
18: erectquione A
f1 (ppm)

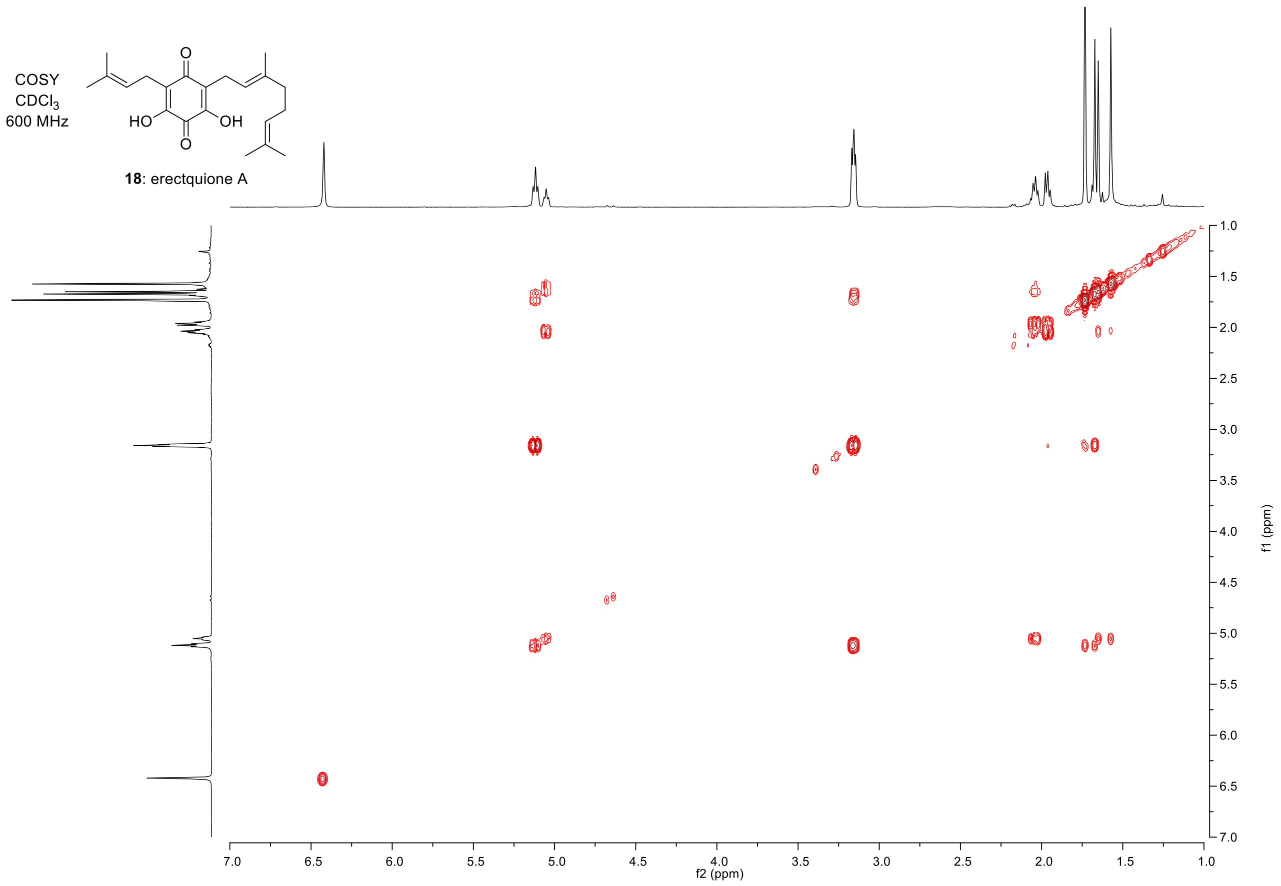
COSY
$CDCl_3$
600 MHz
HO
OH
O
O
18: erectquione A
f1 (ppm)
f2 (ppm)

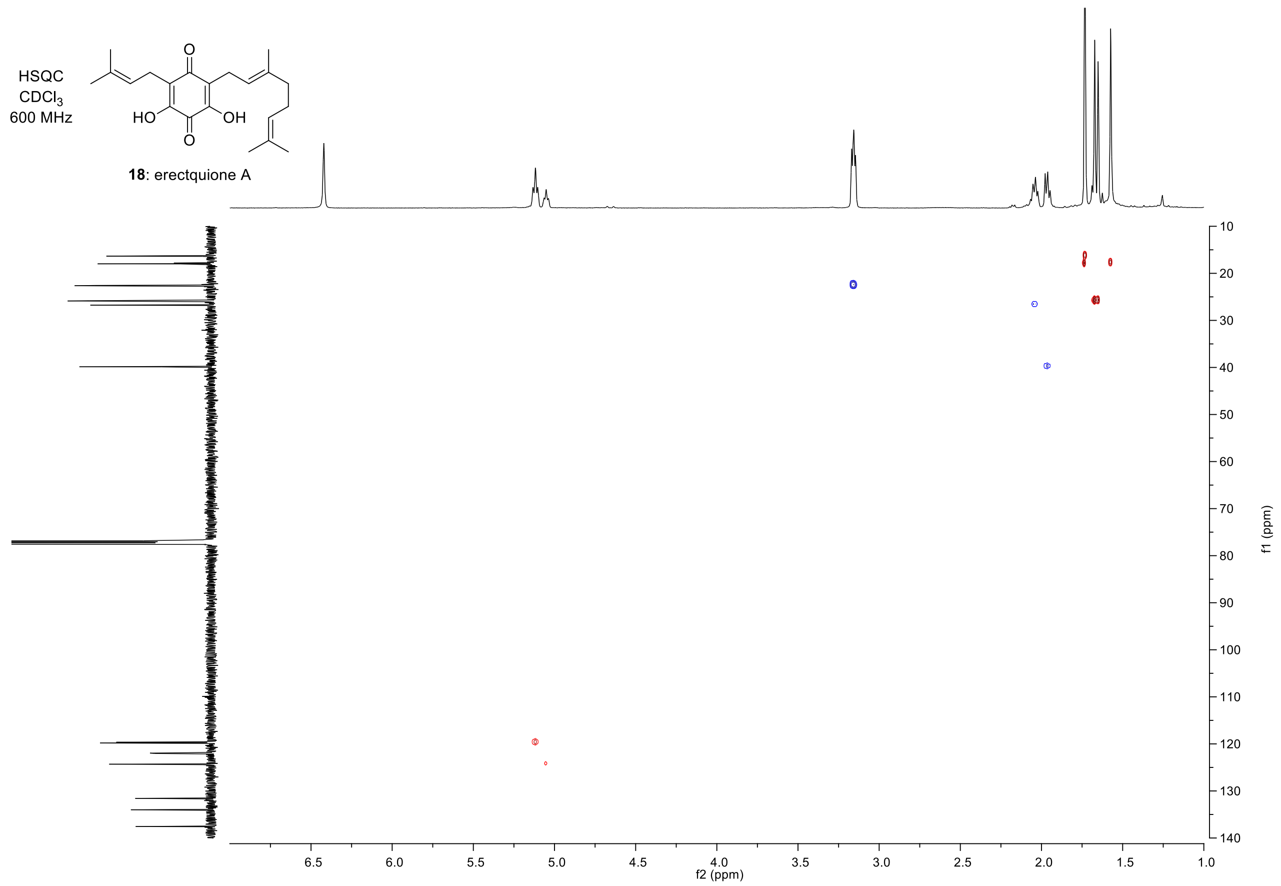
HSQC
$CDCl_3$
600 MHz
HO
OH
O
O
18: erectquione A
f1 (ppm)
f2 (ppm)

HMBC
$CDCl_3$
600 MHz

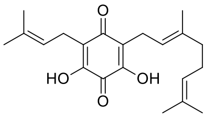

18: erectquione A

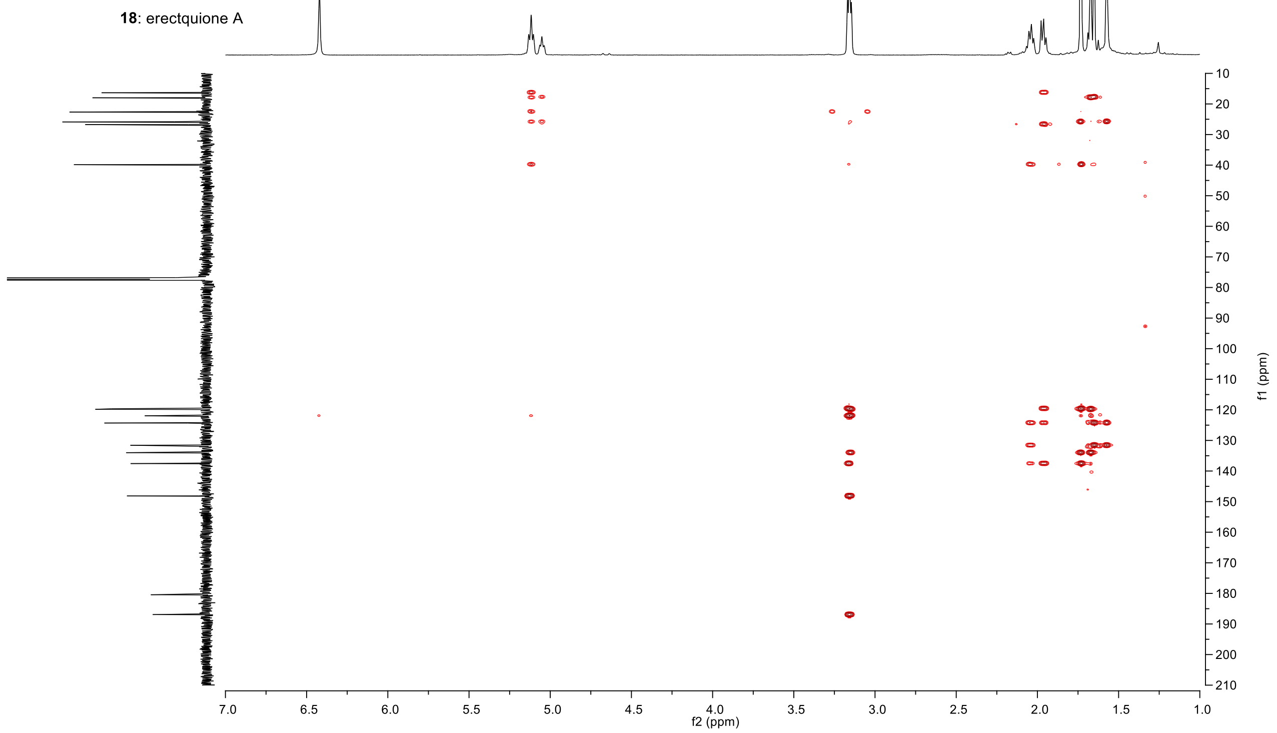

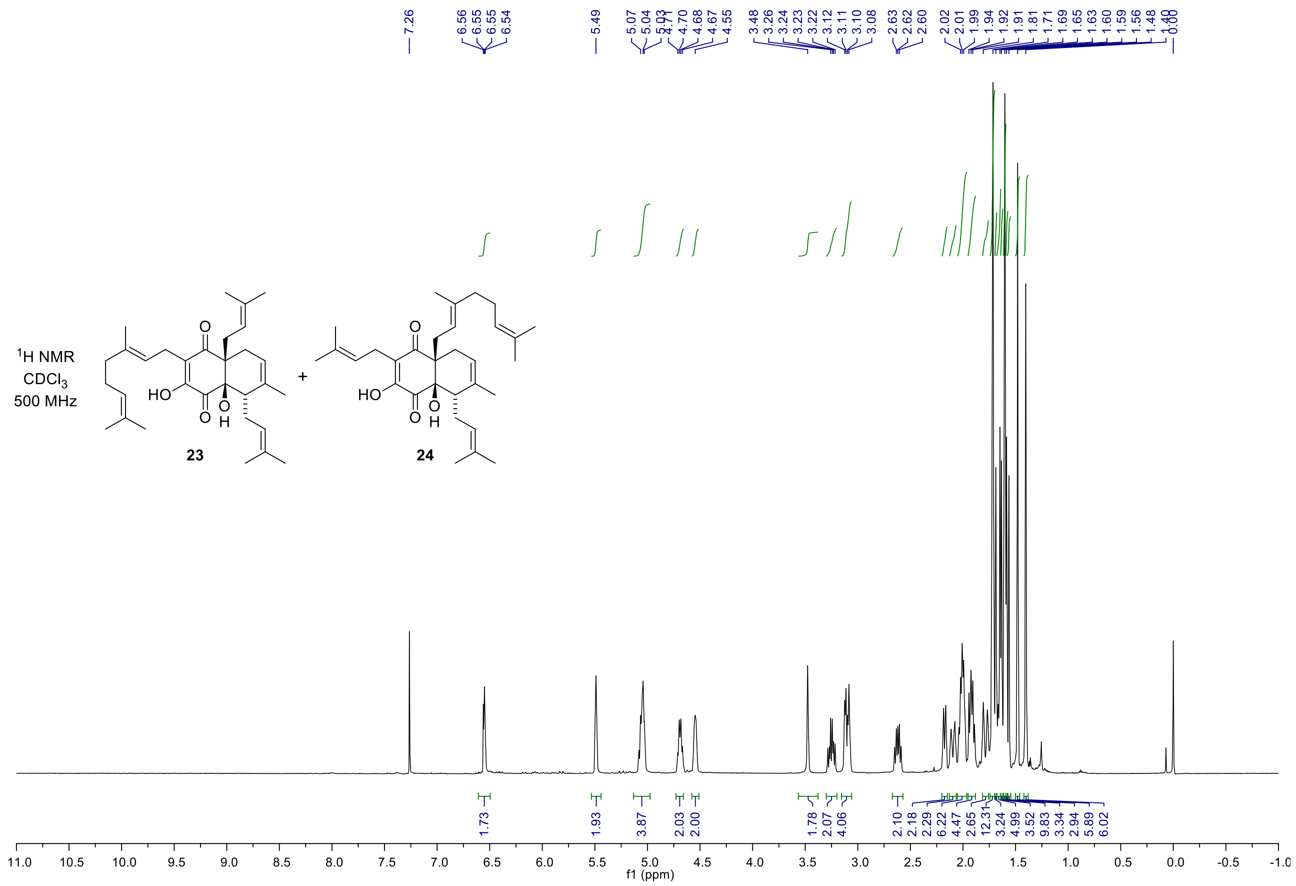
1H NMR
CDCl3
500 MHz
23
24
f1 (ppm)

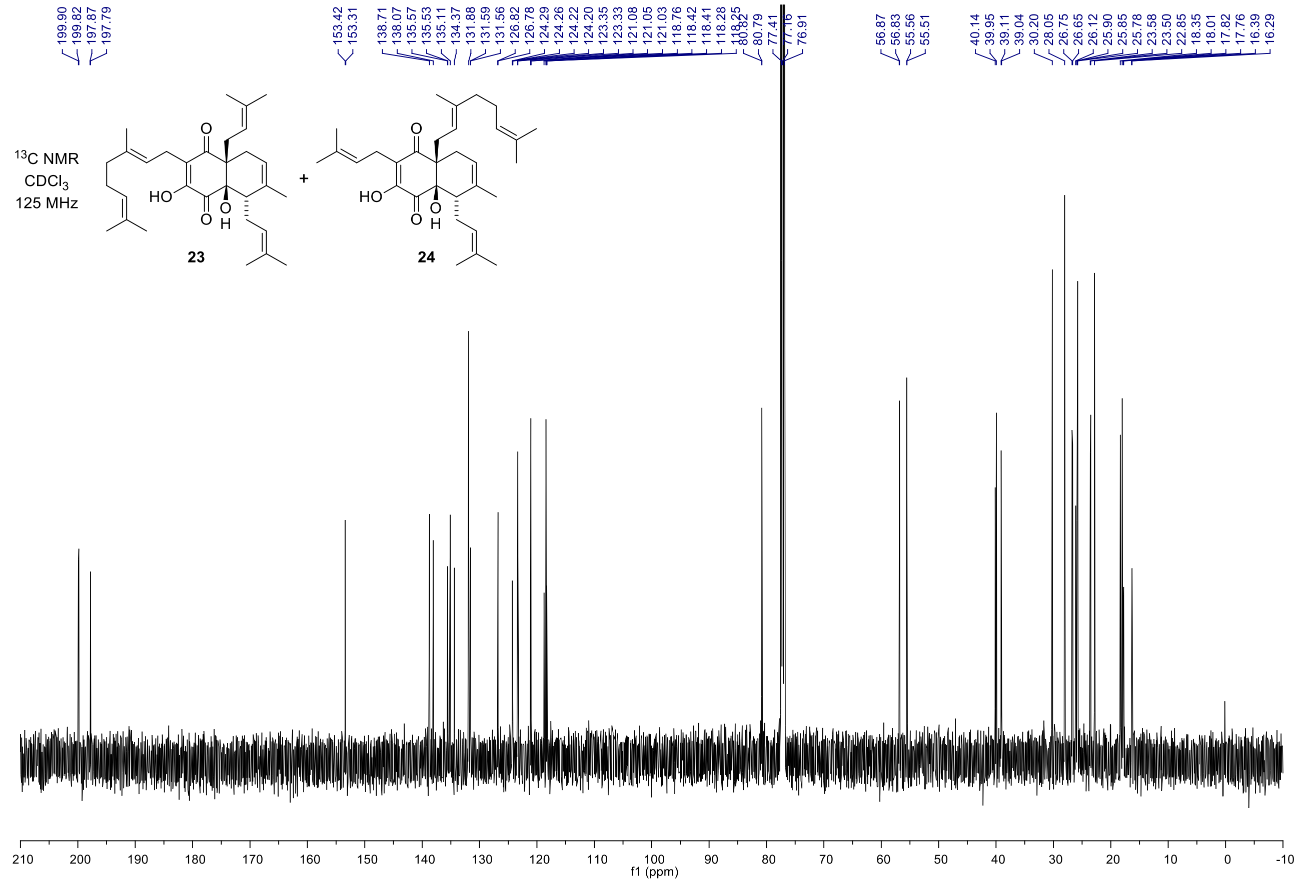
13C NMR
CDCl3
125 MHz
23
24
f1 (ppm)

^{1}H NMR
$CDCl_3$
600 MHz

2: erectone A

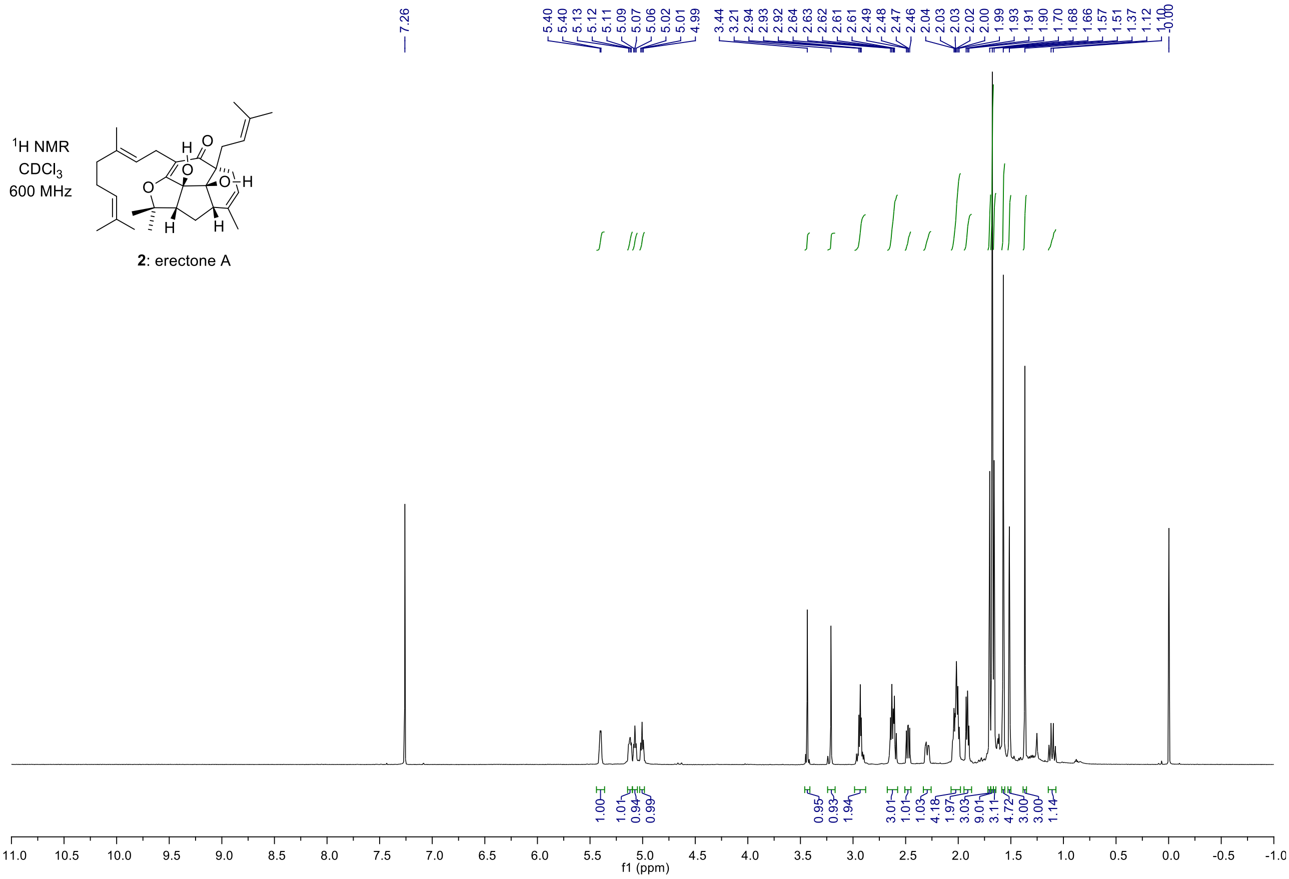

^{13}C NMR
$CDCl_3$
150 MHz

2: erectone A

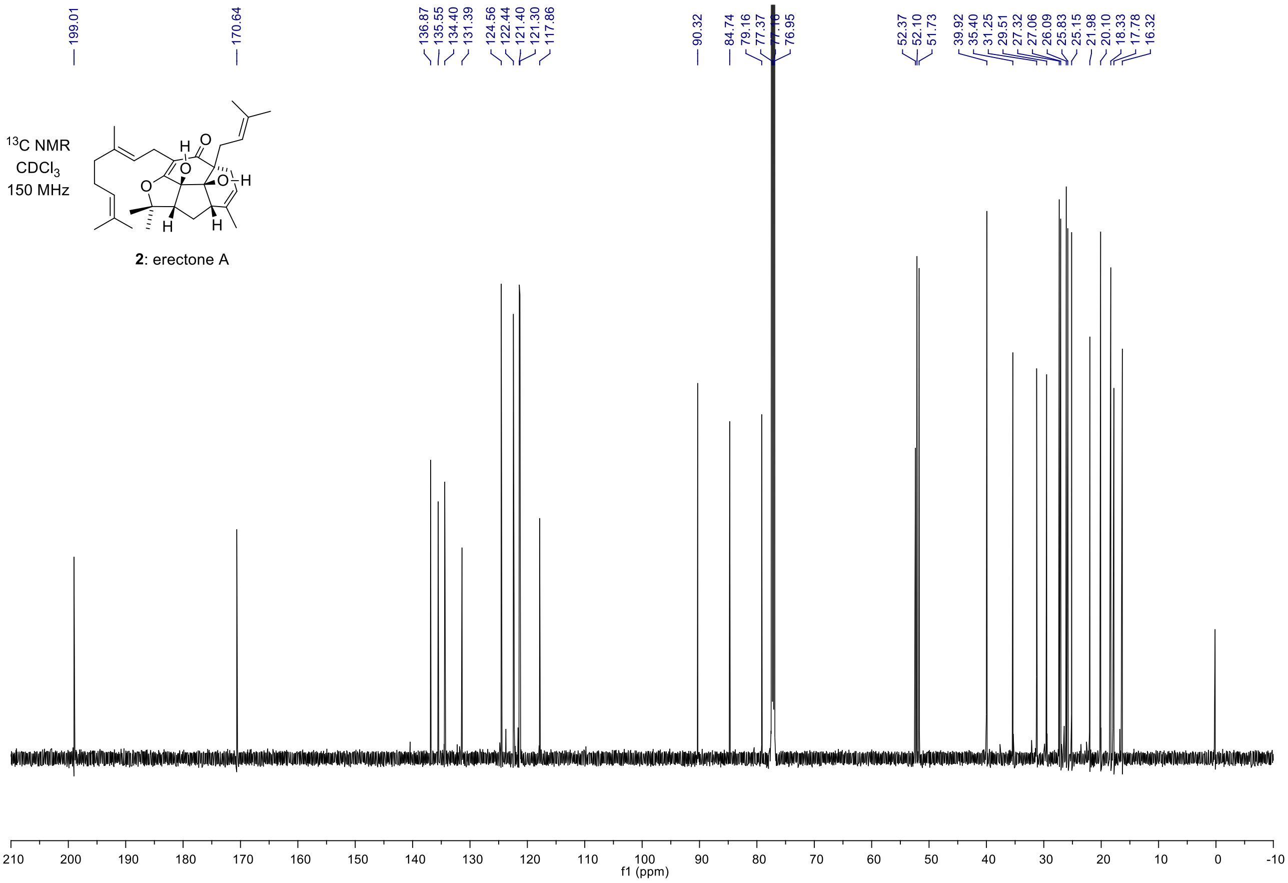

COSY
$CDCl_3$
600 MHz

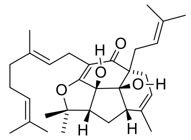

2: erectone A

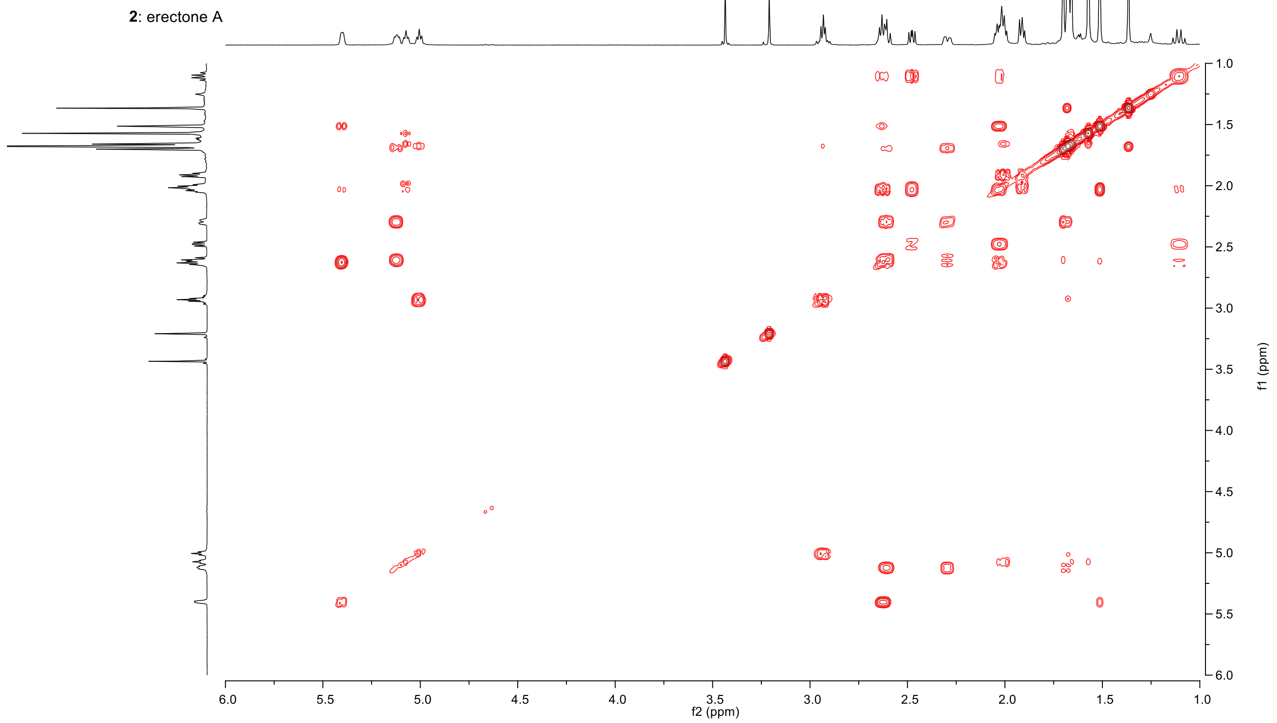

HSQC
$CDCl_3$
600 MHz

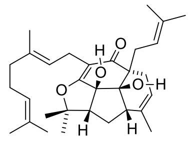

2: erectone A

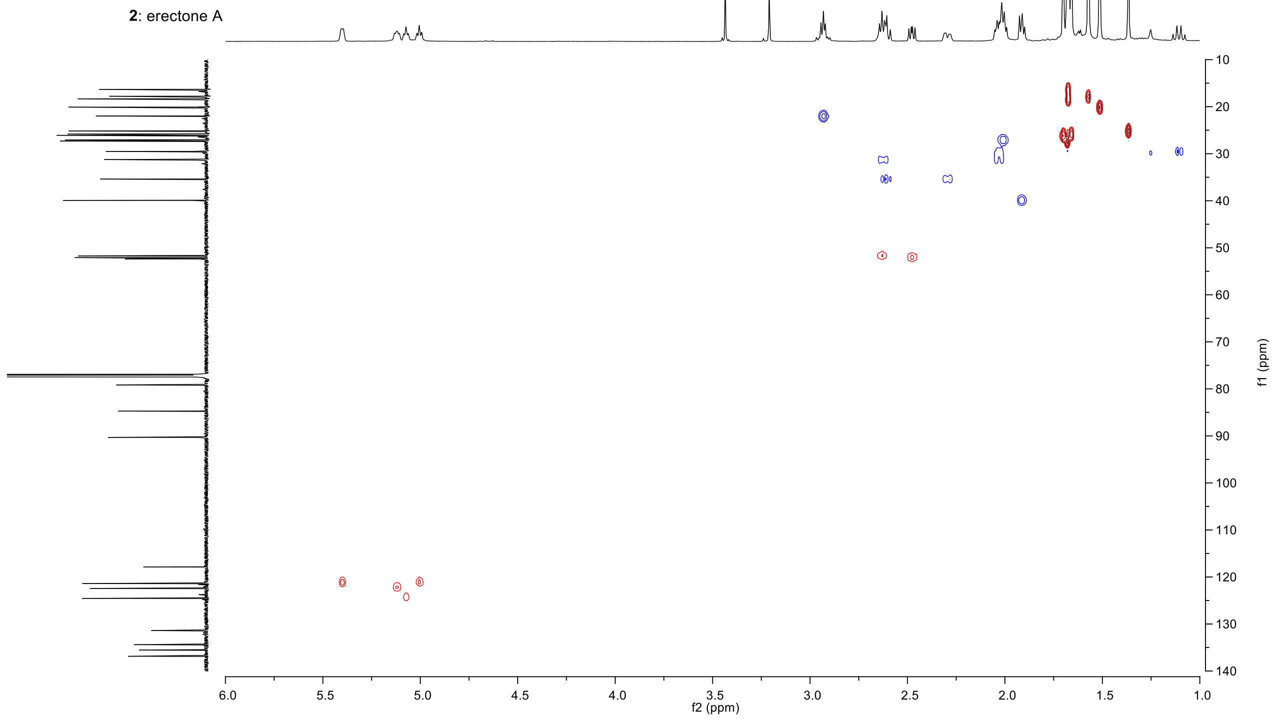

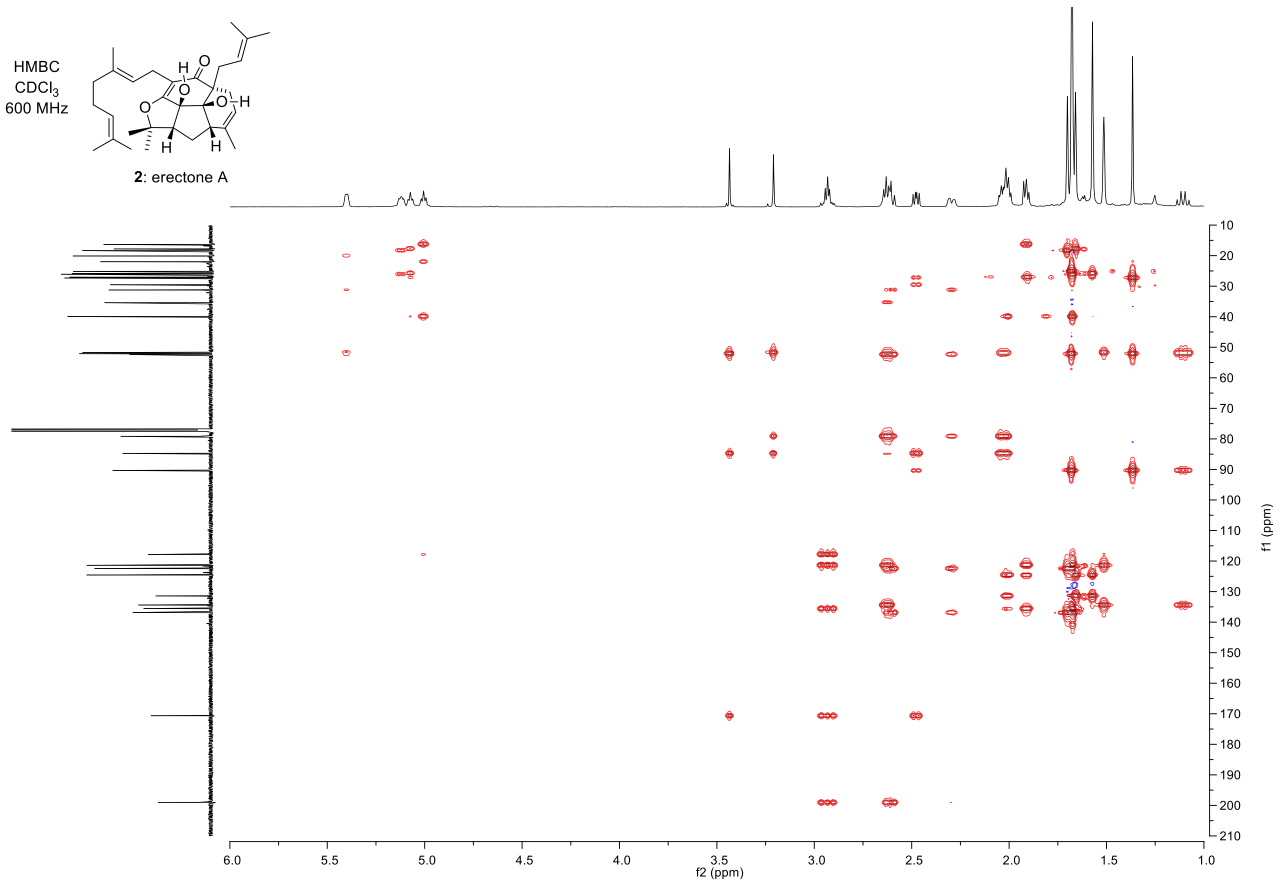
HMBC
$CDCl_3$
600 MHz
2: erectone A
f2 (ppm)
f1 (ppm)

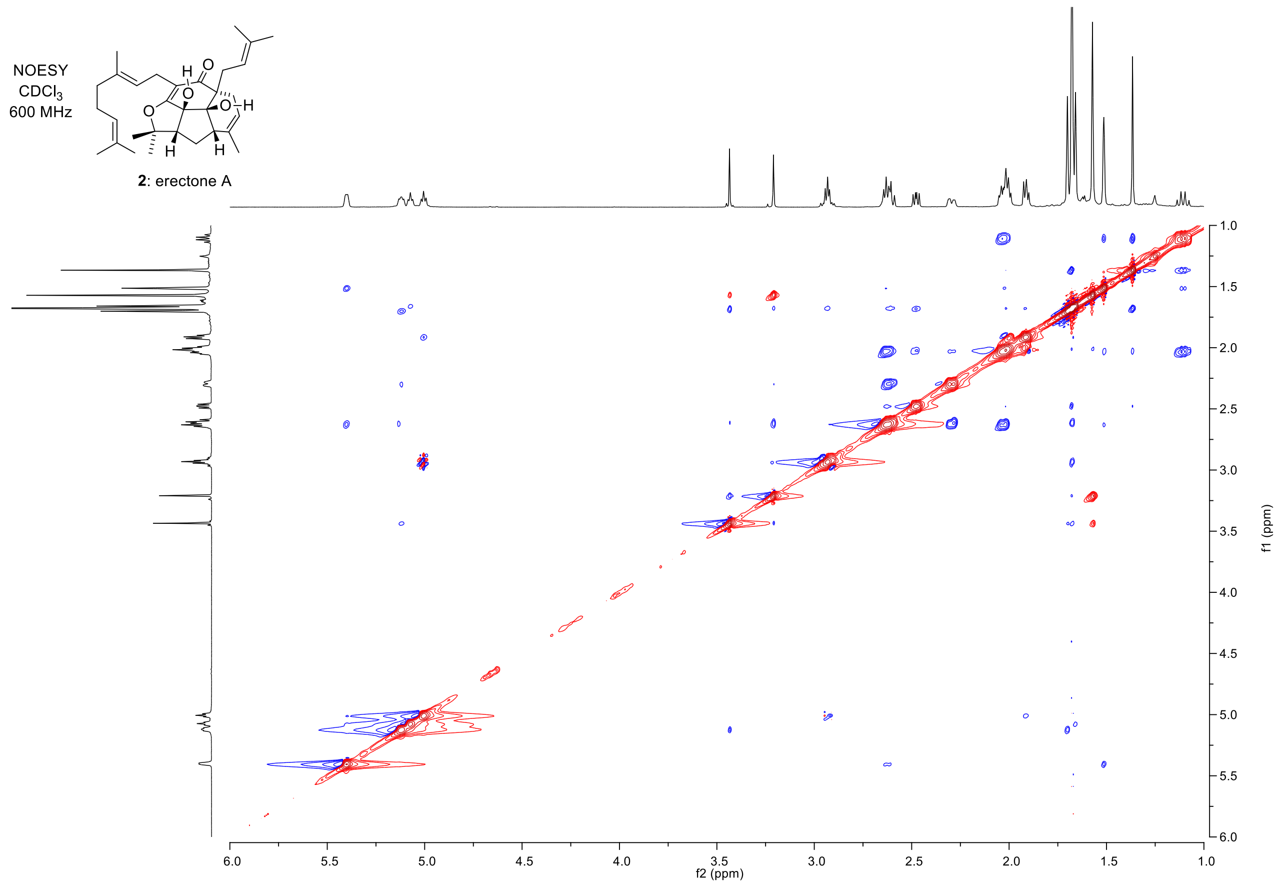
NOESY
$CDCl_3$
600 MHz
2: erectone A
f2 (ppm)
f1 (ppm)

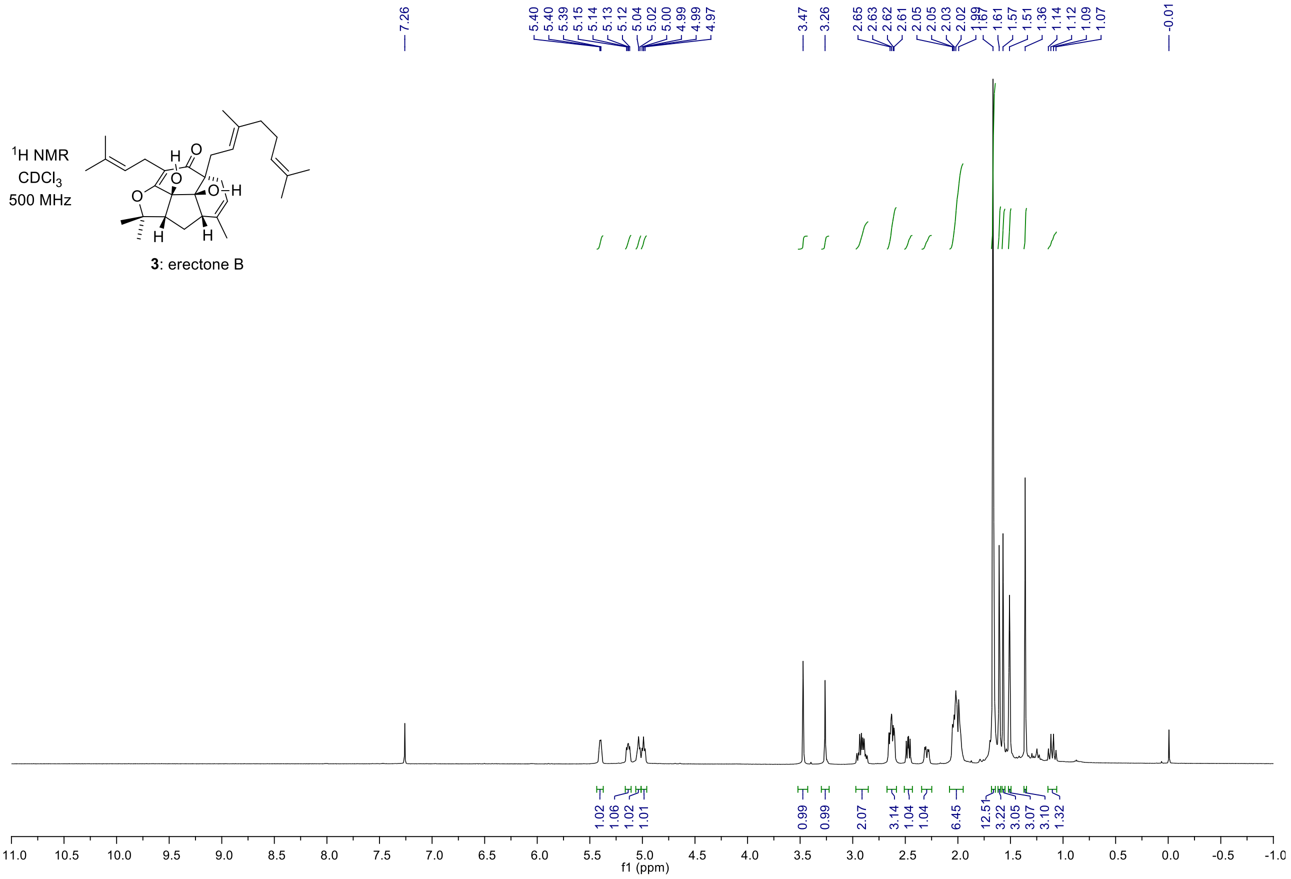
1H NMR
CDCl3
500 MHz
3: erectone B
7.26
5.40 5.40 5.39 5.15 5.14 5.12 5.04 5.02 5.00 4.99 4.97
3.47 3.26
2.65 2.63 2.62 2.61 2.05 2.05 2.03 2.02 1.99 1.97 1.61 1.57 1.51 1.36 1.14 1.12 1.09 1.07
-0.01
1.02 1.06 1.02 1.01 0.99 0.99 2.07 3.14 1.04 1.04 6.45 12.51 3.22 3.05 3.07 3.10 1.32
f1 (ppm)

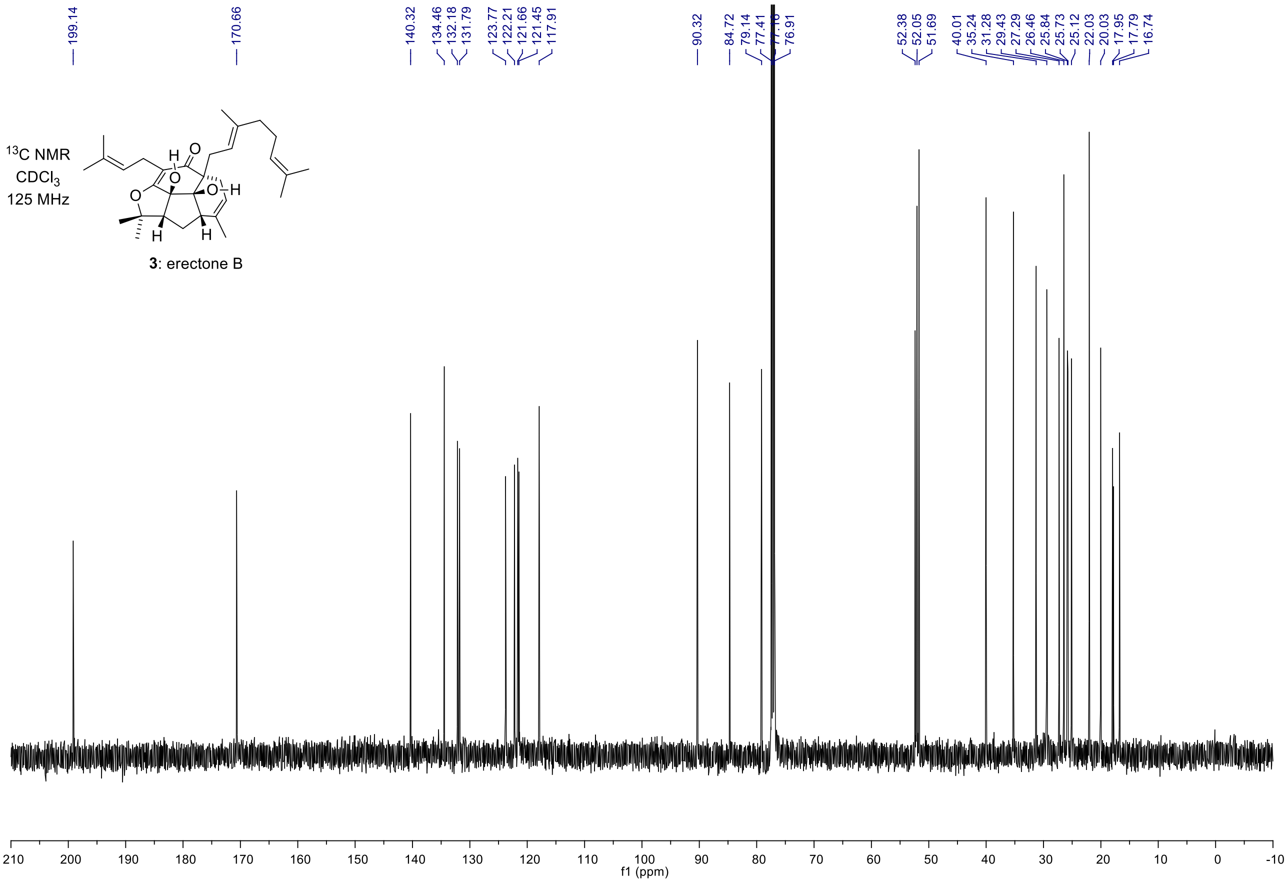
13C NMR
CDCl3
125 MHz
3: erectone B
199.14
170.66
140.32 134.46 132.18 131.79 123.77 122.21 121.66 121.45 117.91
90.32
84.72 79.14 77.41 77.16 76.91
52.38 52.05 51.69
40.01 35.24 31.28 29.43 27.29 26.46 25.84 25.73 25.12 22.03 20.03 17.95 17.79 16.74
f1 (ppm)

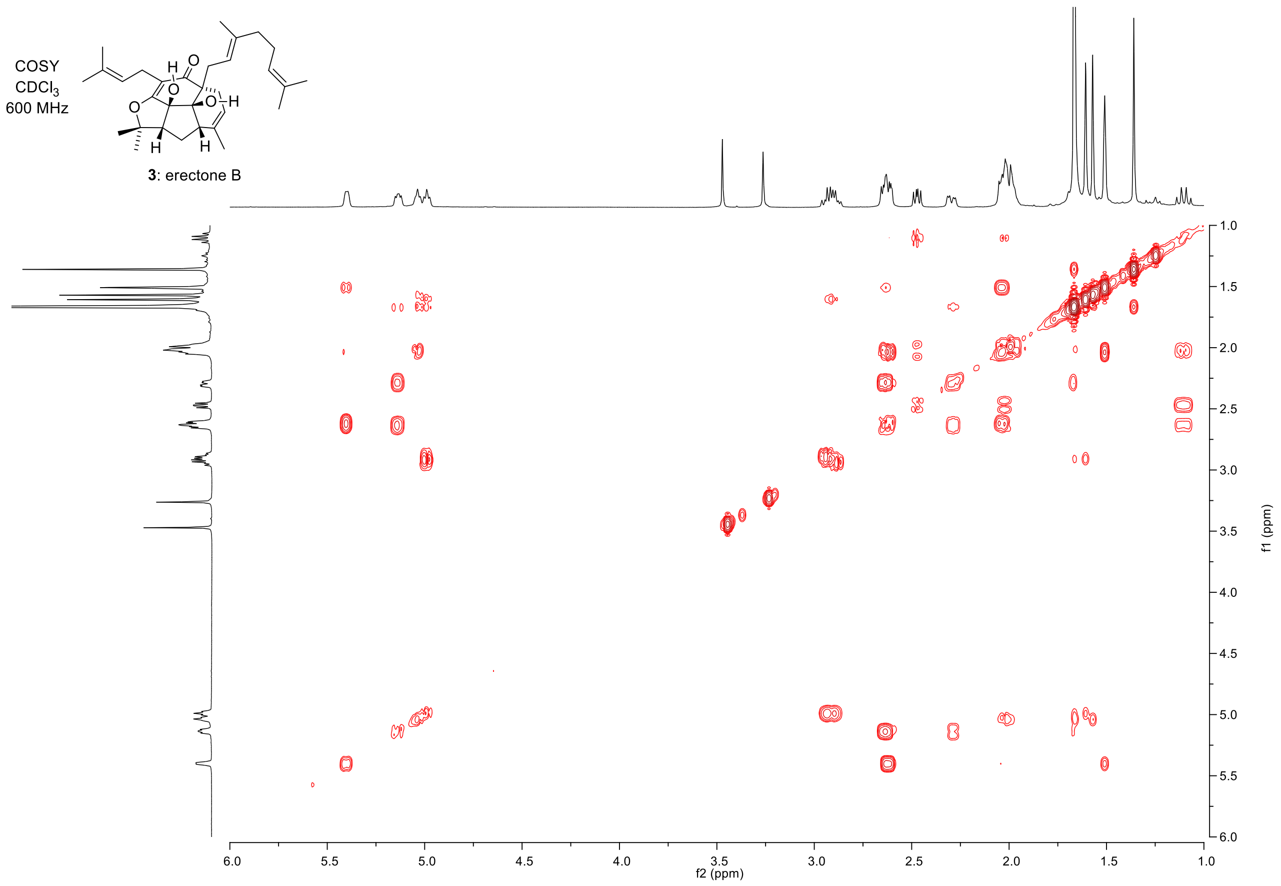

COSY
CDCl3
600 MHz
3: erectone B
f2 (ppm)
f1 (ppm)

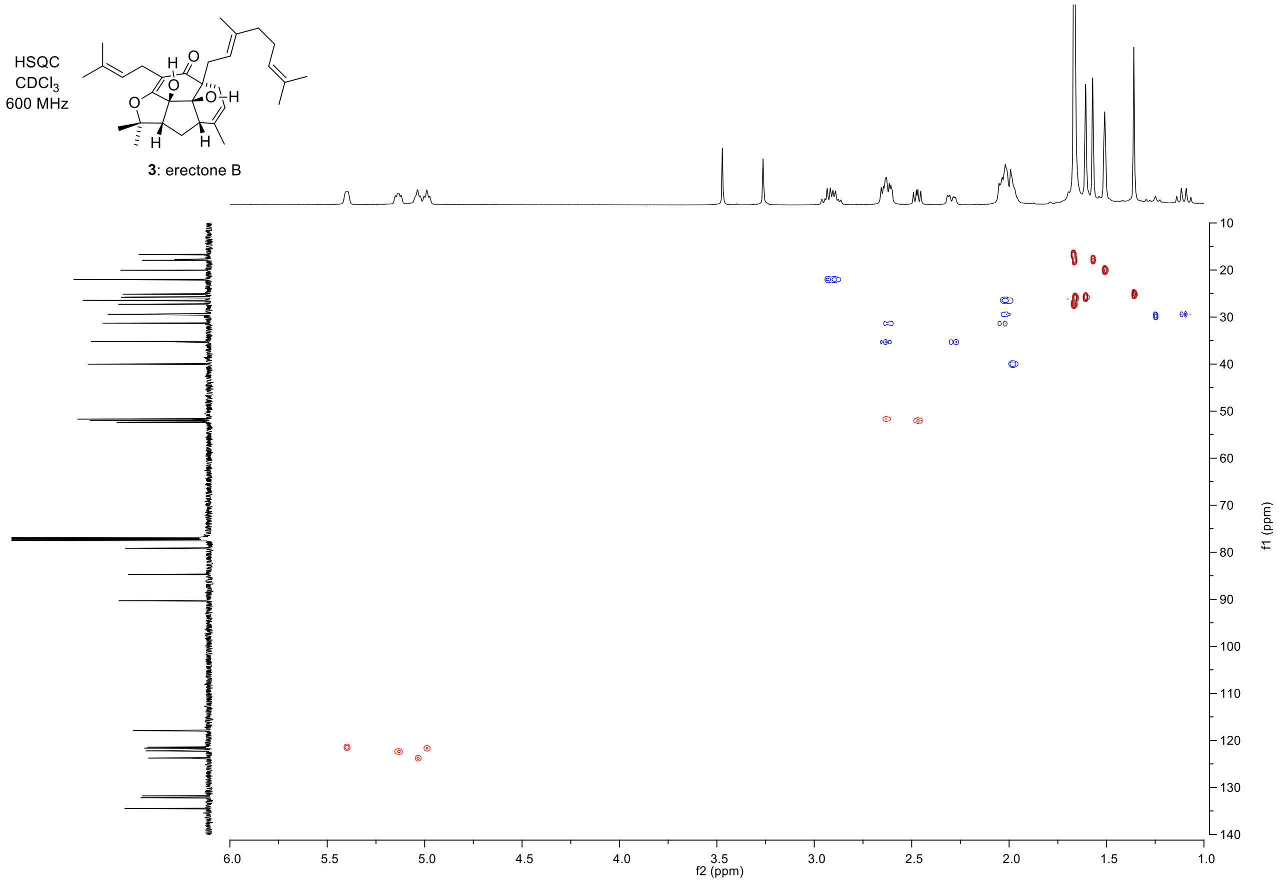

HSQC
CDCl3
600 MHz
3: erectone B
f2 (ppm)
f1 (ppm)

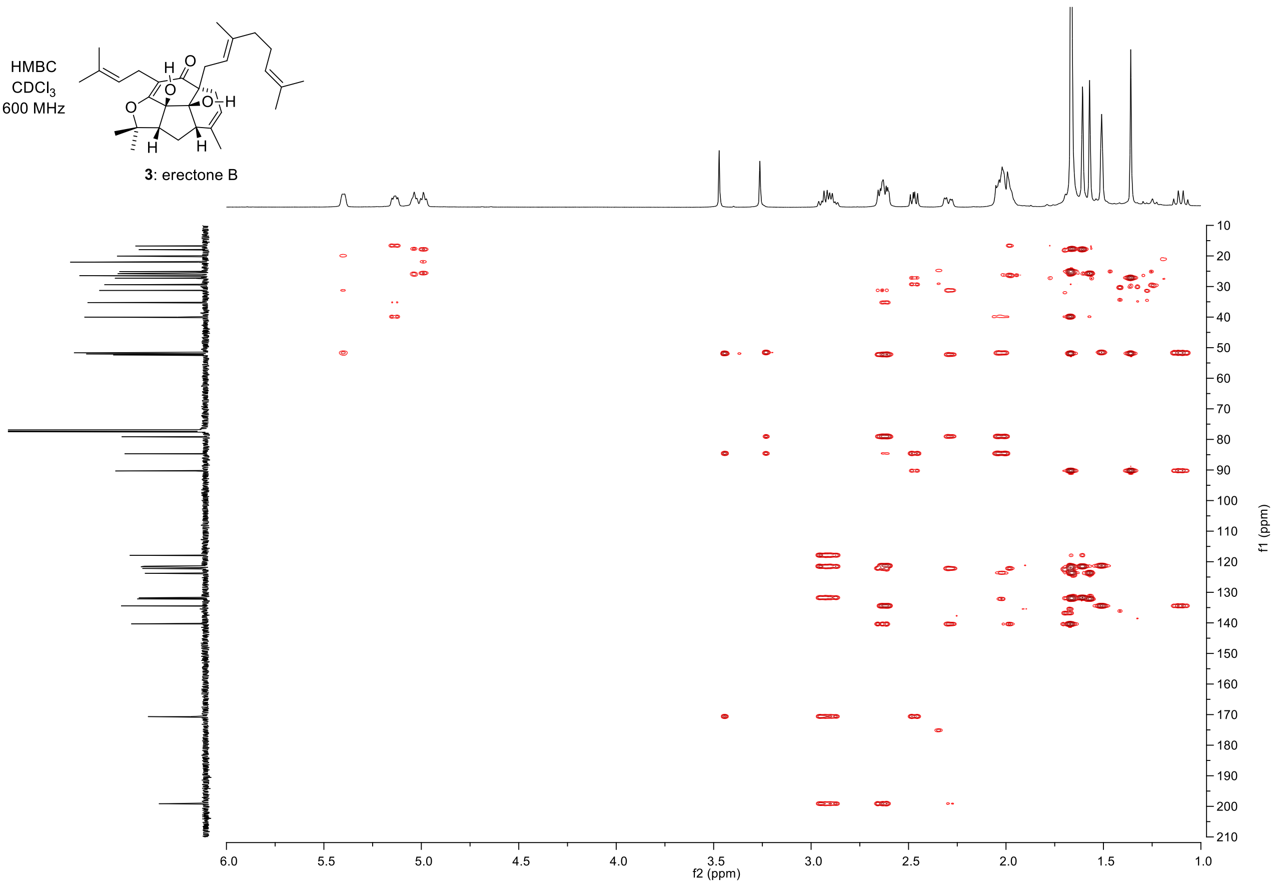
HMBC
$CDCl_3$
600 MHz
3: erectone B
f2 (ppm)
f1 (ppm)

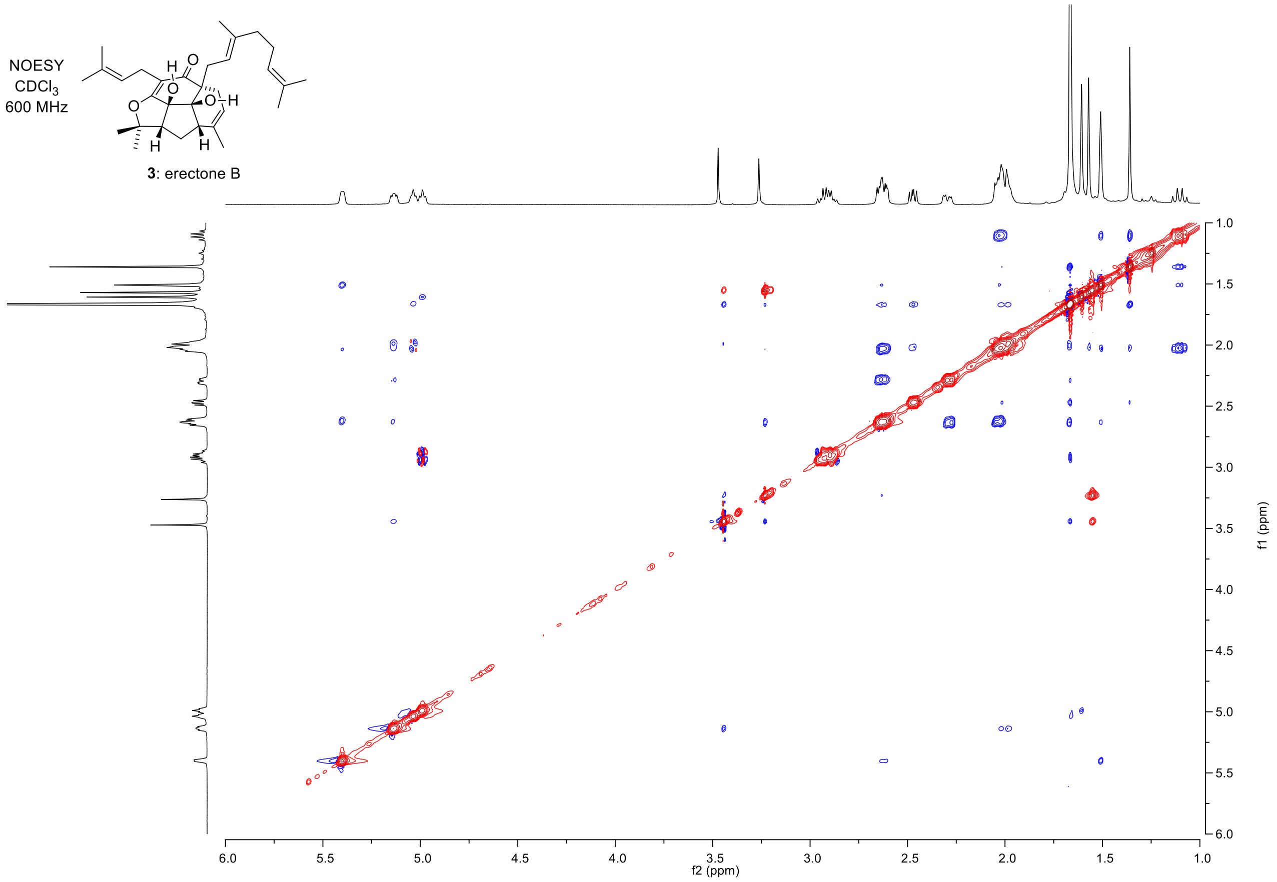
NOESY
$CDCl_3$
600 MHz
3: erectone B
f2 (ppm)
f1 (ppm)

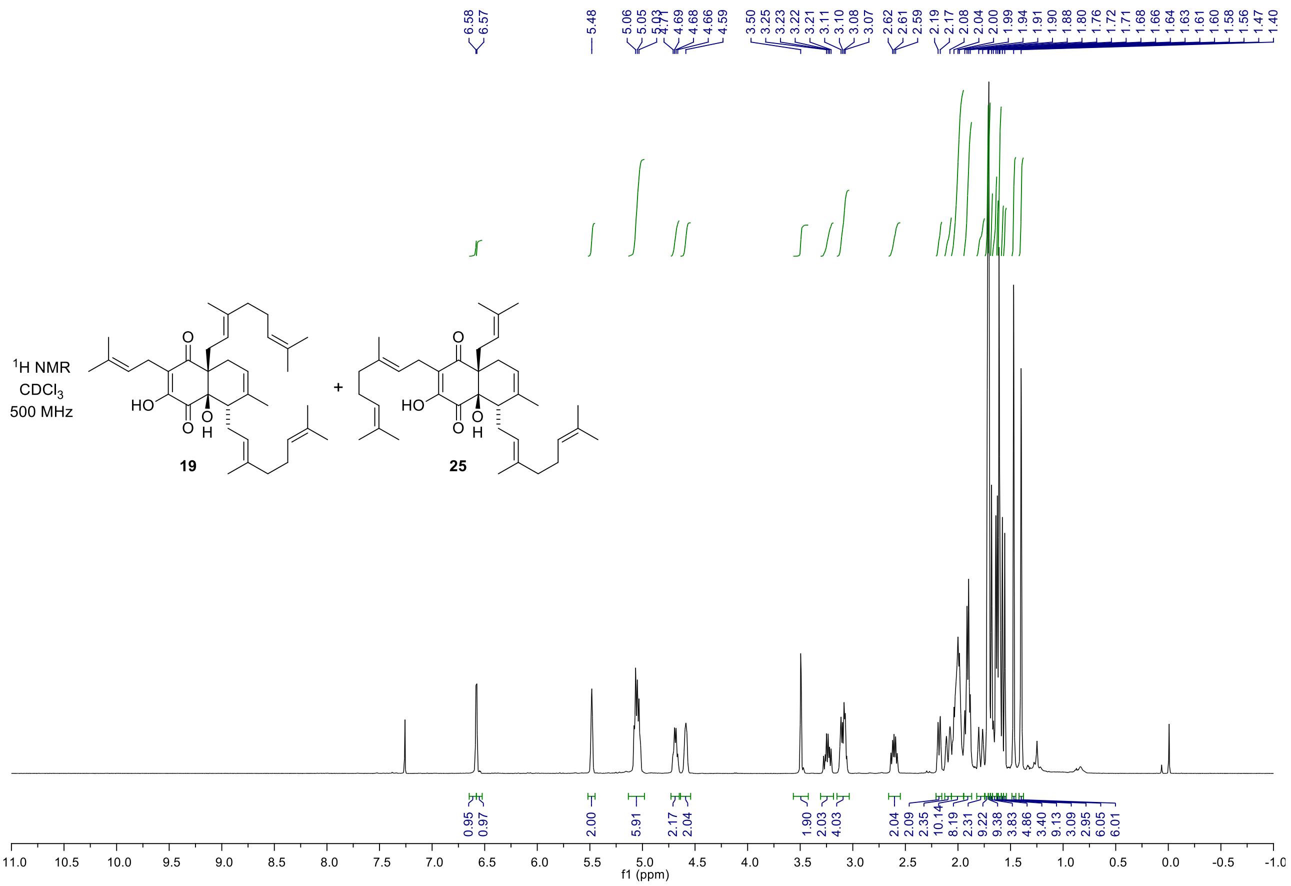
6.58
6.57
5.48
5.06
5.05
4.69
4.68
4.66
4.59
3.50
3.25
3.23
3.22
3.21
3.11
3.10
3.08
3.07
2.62
2.61
2.59
2.19
2.17
2.08
2.04
2.00
1.99
1.94
1.91
1.90
1.88
1.80
1.76
1.72
1.71
1.68
1.66
1.64
1.63
1.61
1.60
1.58
1.56
1.47
1.40
1H NMR
CDCl3
500 MHz
O
HO
O
H
19
+
25
0.95
0.97
2.00
5.91
2.17
2.04
1.90
2.03
4.03
2.04
2.09
2.35
10.14
8.19
2.31
9.22
9.38
3.83
4.86
3.40
9.13
3.09
2.95
6.05
6.01
f1 (ppm)

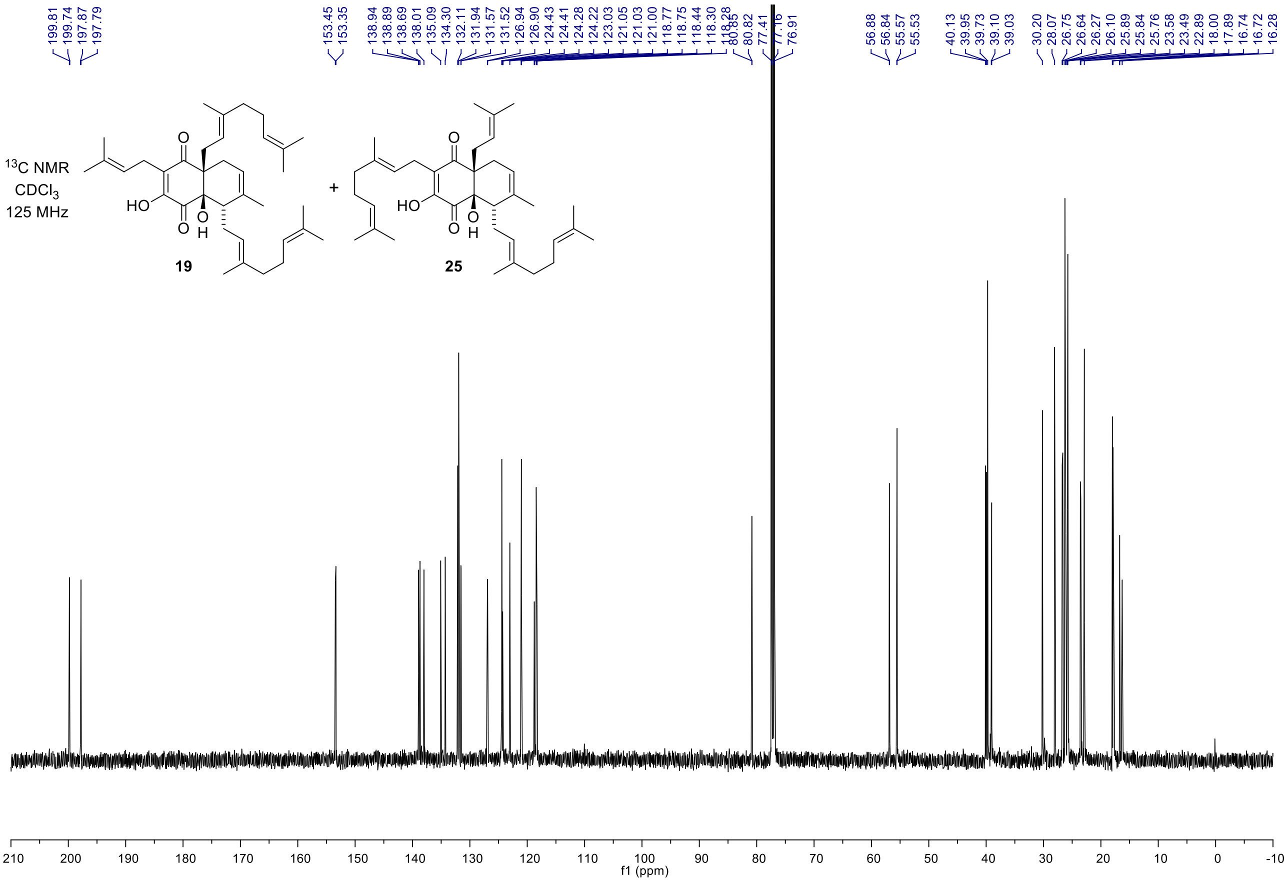
199.81
199.74
197.87
197.79
153.45
153.35
138.94
138.89
138.69
138.01
135.09
134.30
132.11
131.94
131.57
131.52
126.94
126.90
124.43
124.41
124.28
124.22
123.03
121.05
121.03
121.00
118.77
118.75
118.44
118.30
80.82
77.41
77.16
76.91
56.88
56.84
55.57
55.53
40.13
39.95
39.73
39.10
39.03
30.20
28.07
26.75
26.64
26.27
26.10
25.89
25.84
25.76
23.58
23.49
22.89
18.00
17.89
16.74
16.72
16.28
13C NMR
CDCl3
125 MHz
O
HO
O
H
19
+
25
f1 (ppm)

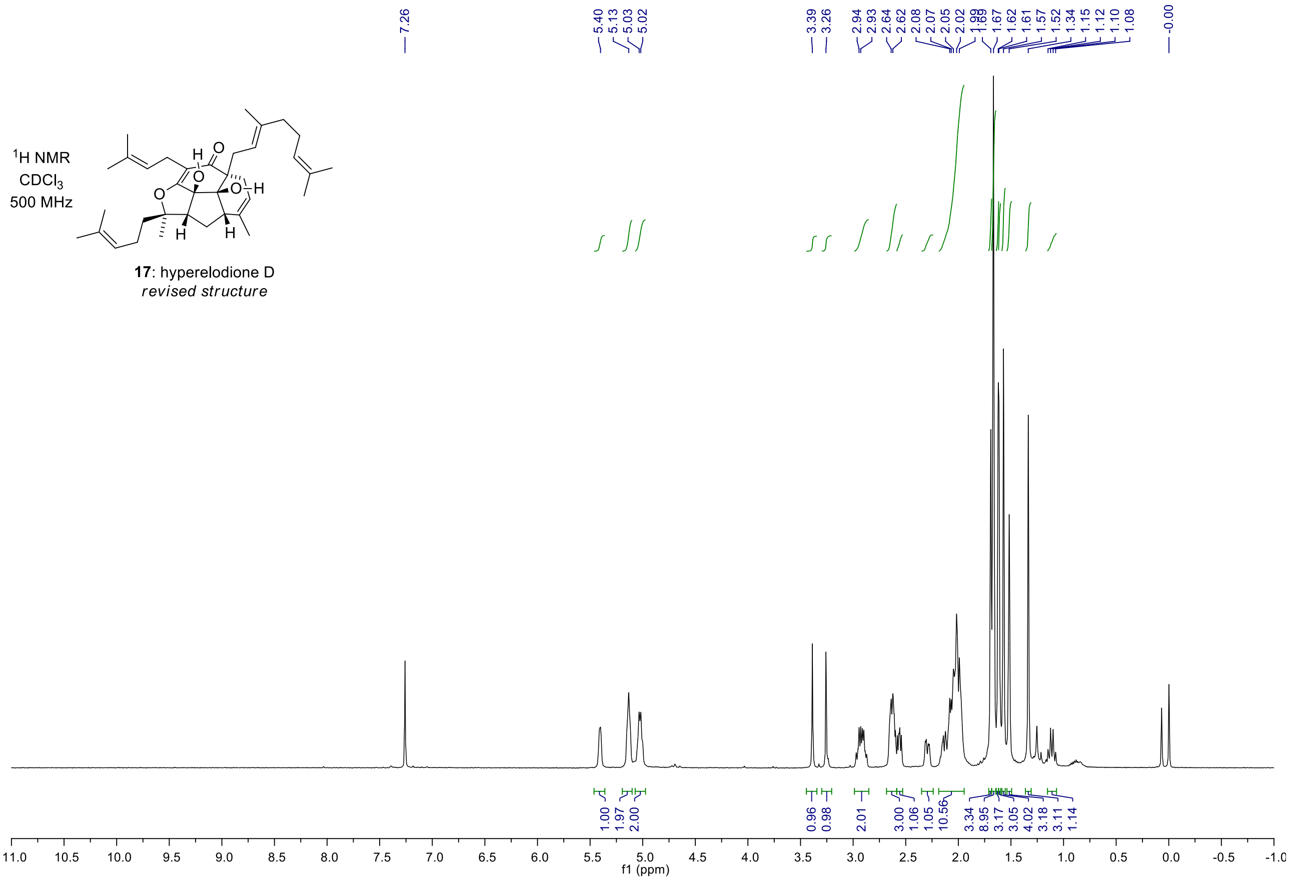

^{1}H NMR
$CDCl_3$
500 MHz
17: hyperelodione D
revised structure
7.26
5.40 5.13 5.03 5.02
3.39 3.26
2.94 2.93 2.64 2.62 2.08 2.07 2.05 2.02 1.67 1.62 1.61 1.57 1.52 1.34 1.15 1.12 1.10 1.08
-0.00
1.00 1.97 2.00 0.96 0.98 2.01 3.00 1.06 1.05 10.56 3.34 8.95 3.17 3.05 4.02 3.18 3.11 1.14
f1 (ppm)

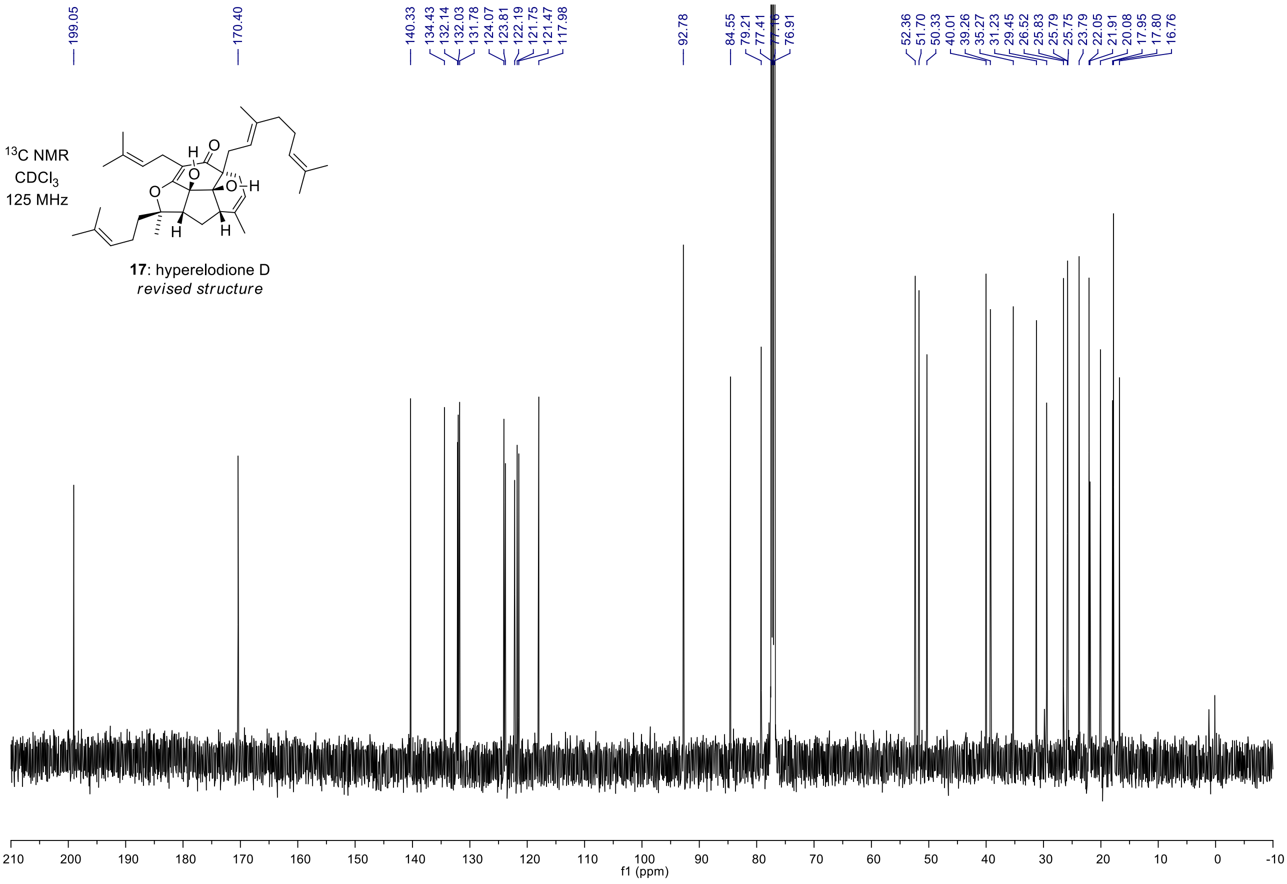

^{13}C NMR
$CDCl_3$
125 MHz
17: hyperelodione D
revised structure
199.05
170.40
140.33 134.43 132.14 132.03 131.78 124.07 123.81 122.19 121.75 121.47 117.98
92.78
84.55 79.21 77.41 76.91
52.36 51.70 50.33 40.01 39.26 35.27 31.23 29.45 26.52 25.83 25.79 25.75 23.79 22.05 21.91 20.08 17.95 17.80 16.76
f1 (ppm)

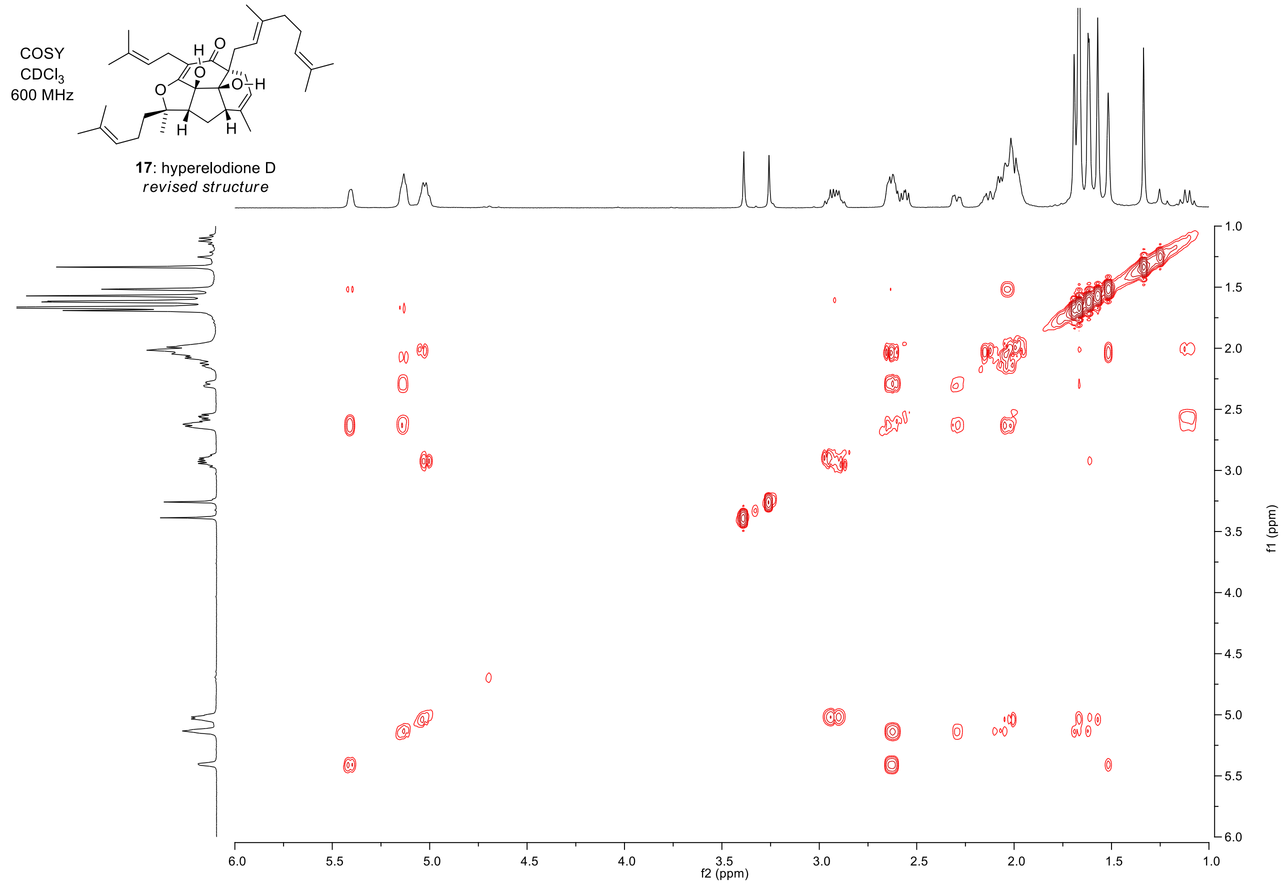
COSY
CDCl3
600 MHz
H
O
O
O
H
O
H
H
17: hyperelodione D
revised structure
f2 (ppm)
f1 (ppm)

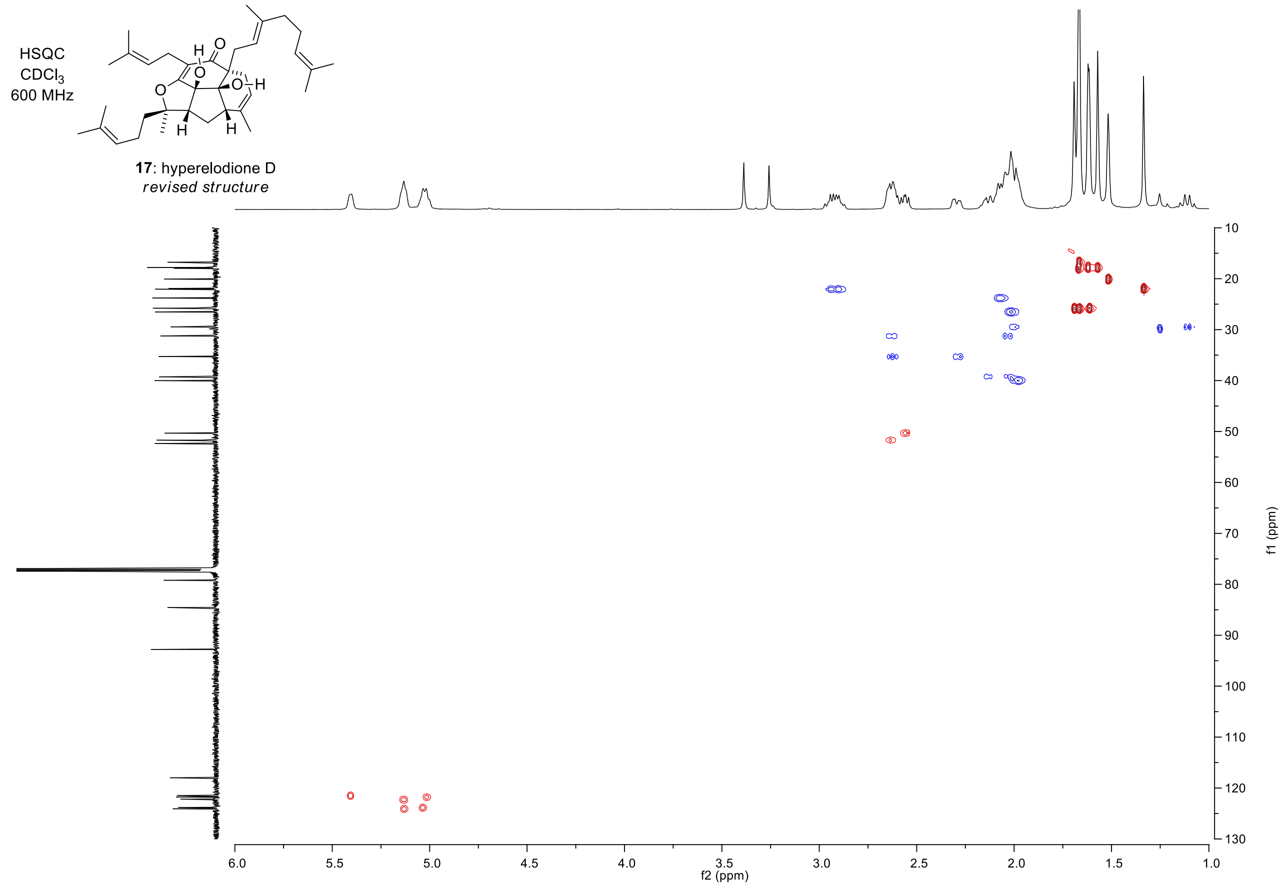
HSQC
CDCl3
600 MHz
H
O
O
O
H
O
H
H
17: hyperelodione D
revised structure
f2 (ppm)
f1 (ppm)

HMBC
$CDCl_3$
600 MHz

17: hyperelodione D
revised structure

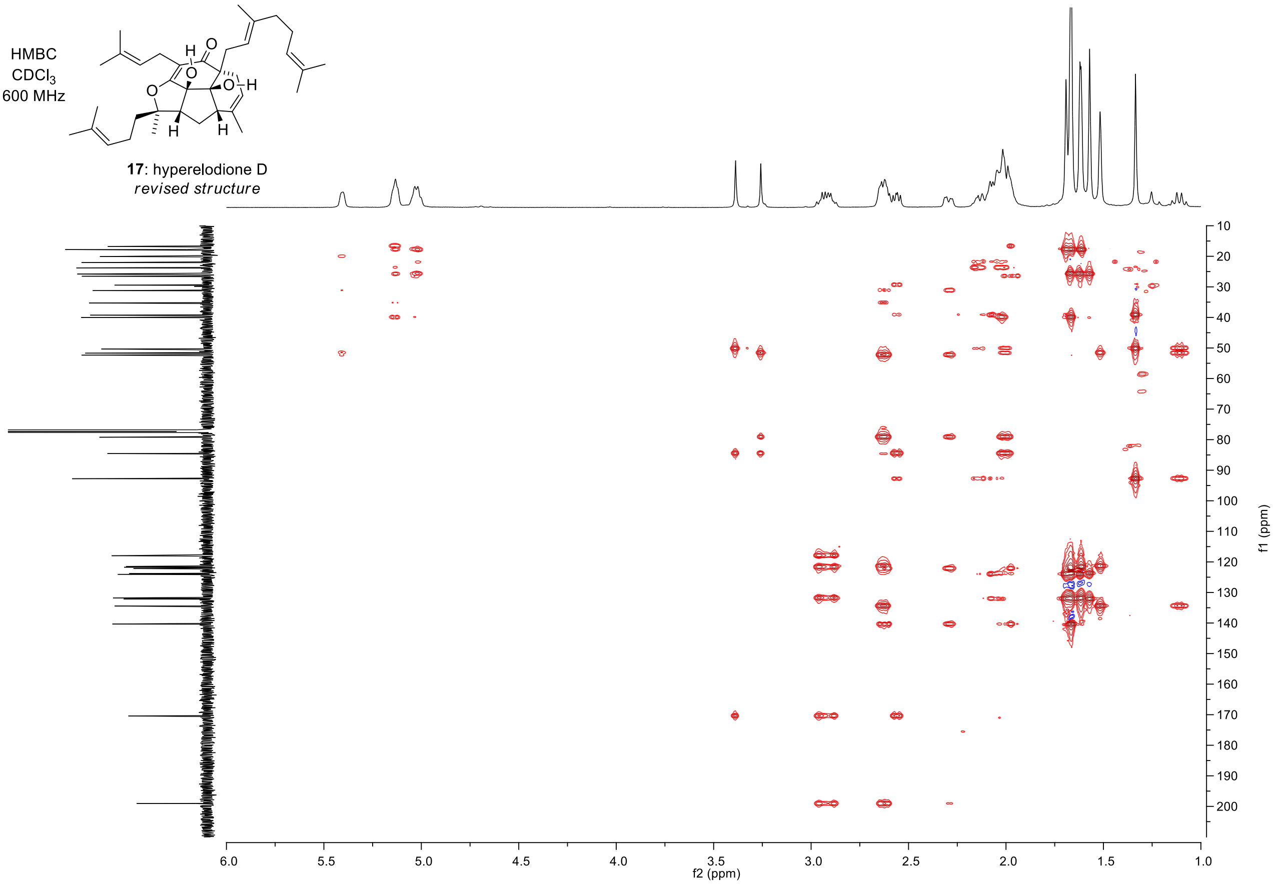

NOESY
$CDCl_3$
600 MHz

17: hyperelodione D
revised structure

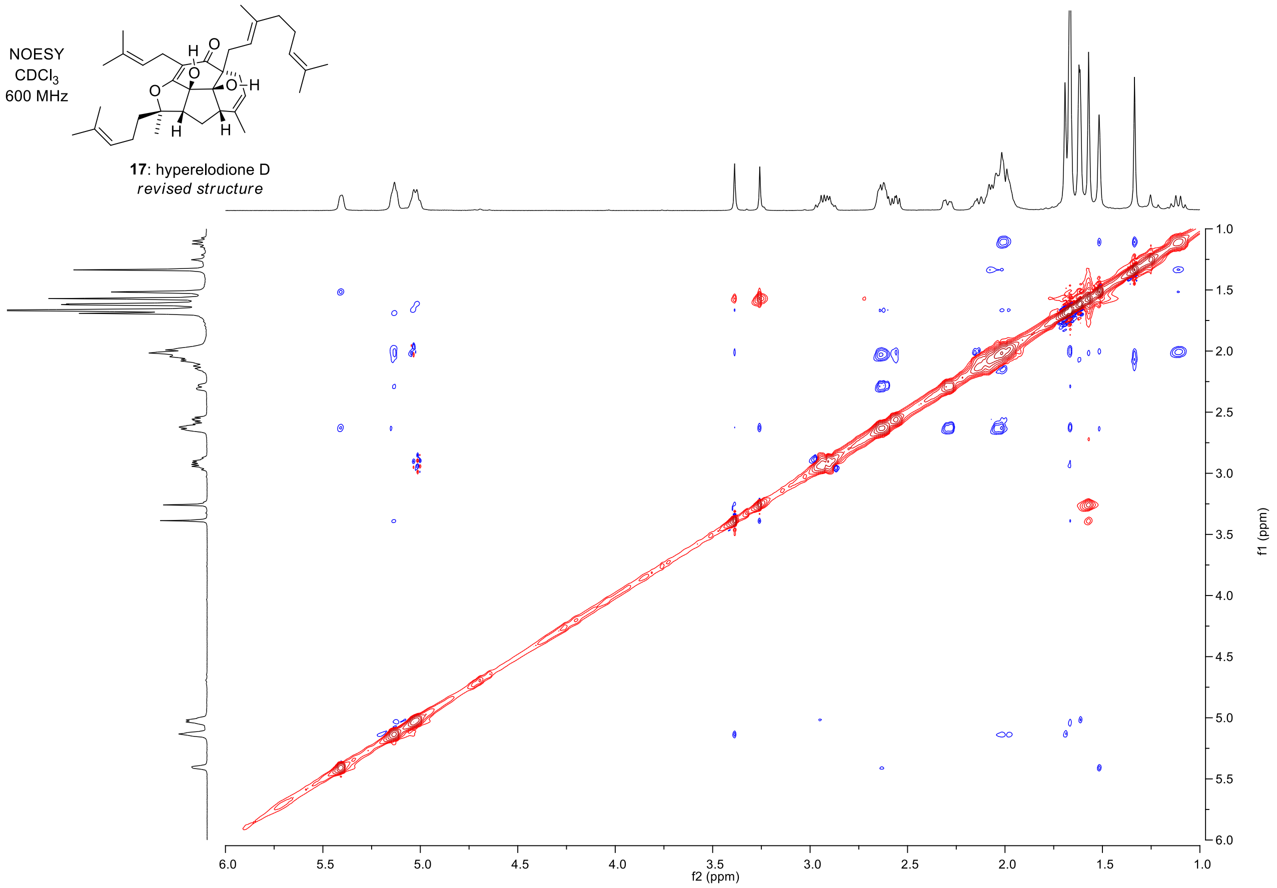

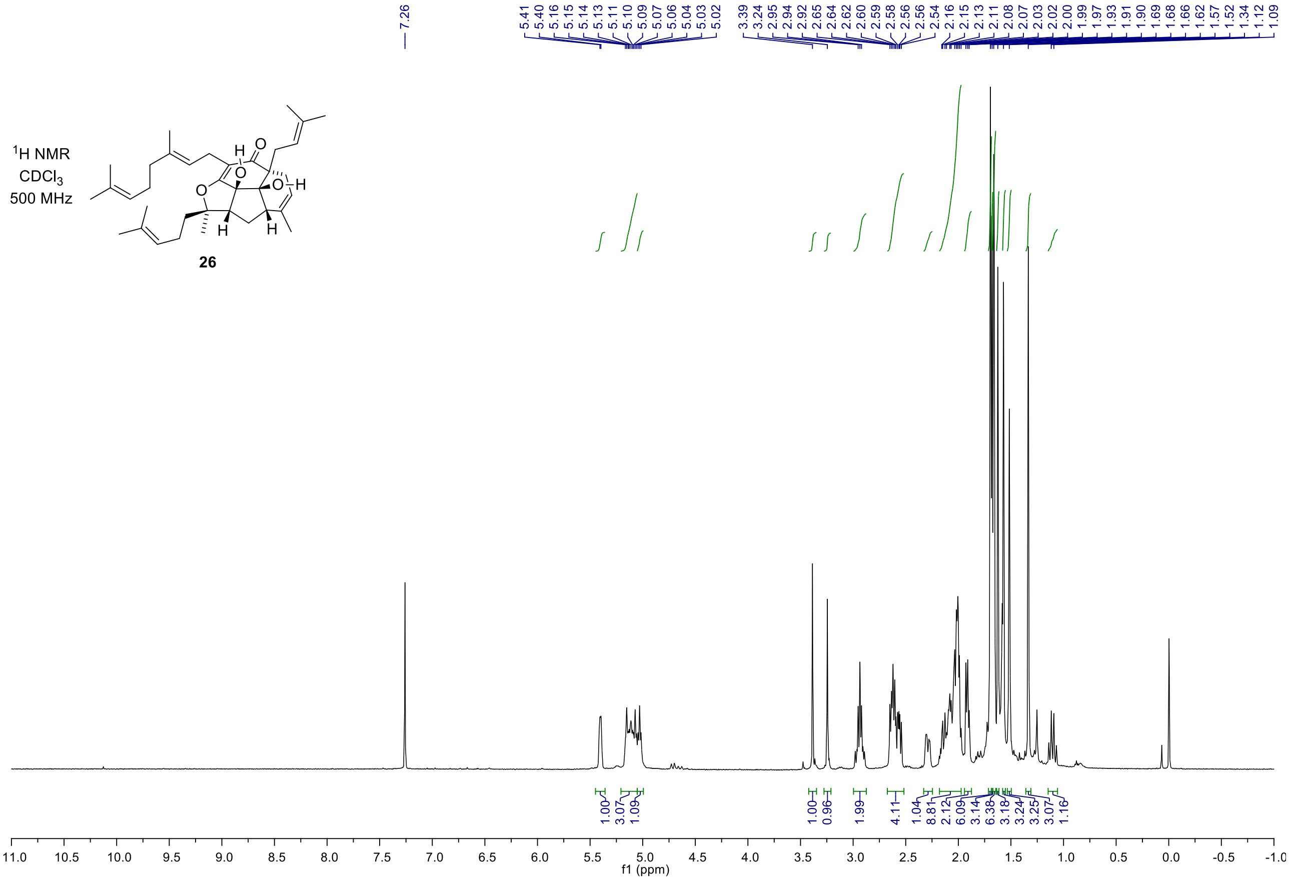
^{1}H NMR
$CDCl_3$
500 MHz
26
7.26
5.41 5.40 5.16 5.15 5.14 5.13 5.11 5.10 5.09 5.07 5.06 5.04 5.03 5.02
3.39 3.24 2.95 2.94 2.92 2.65 2.64 2.62 2.60 2.59 2.58 2.56 2.56 2.54 2.16 2.15 2.13 2.11 2.08 2.07 2.03 2.02 2.00 1.99 1.97 1.93 1.91 1.90 1.69 1.68 1.66 1.62 1.57 1.52 1.34 1.12 1.09
1.00 3.07 1.09 1.00 0.96 1.99 4.11 1.04 8.81 2.12 6.09 3.14 6.38 3.18 3.24 3.25 3.07 1.16
f1 (ppm)

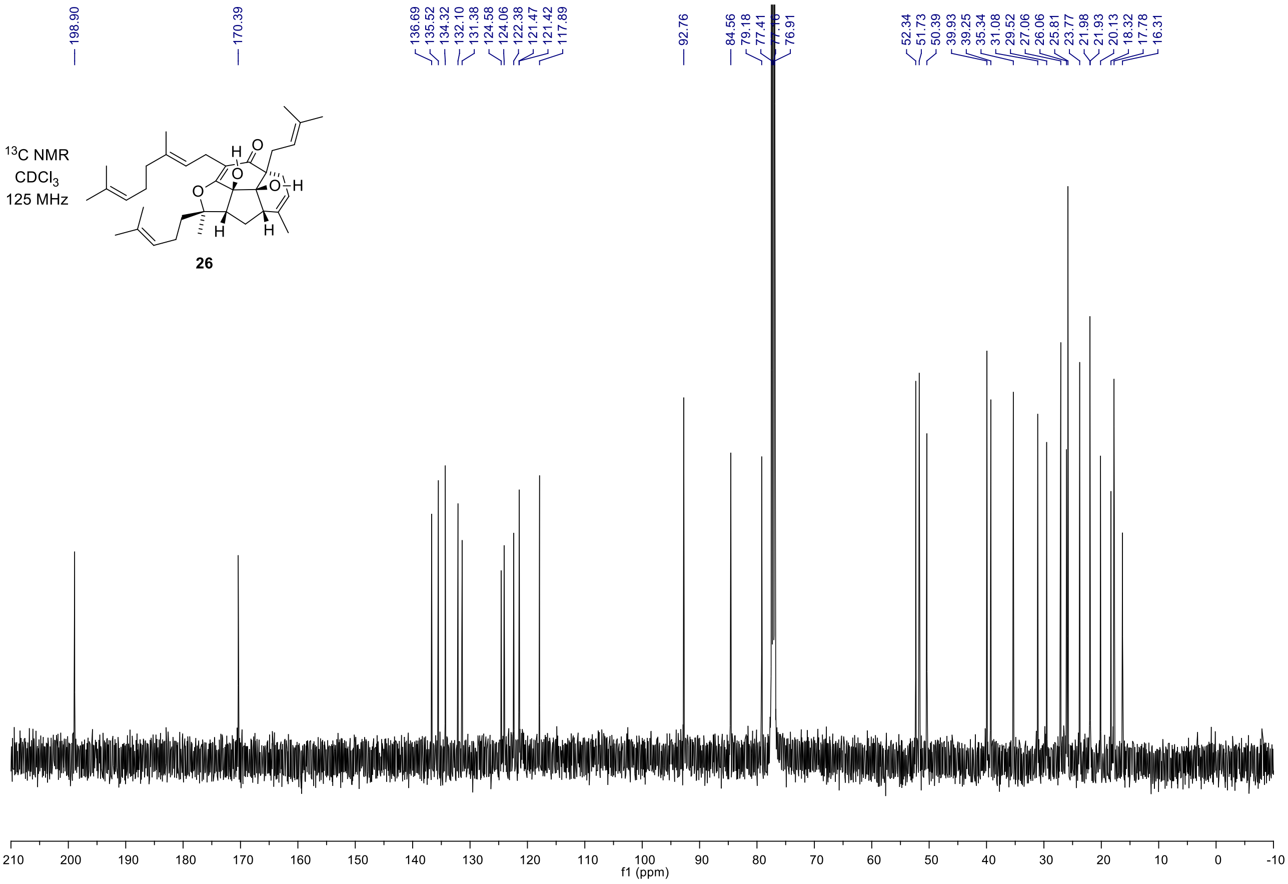
^{13}C NMR
$CDCl_3$
125 MHz
26
198.90
170.39
136.69 135.52 134.32 132.10 131.38 124.58 124.06 122.38 121.47 121.42 117.89
92.76
84.56 79.18 77.41 76.91
52.34 51.73 50.39 39.93 39.25 35.34 31.08 29.52 27.06 26.06 25.81 23.77 21.98 21.93 20.13 18.32 17.78 16.31
f1 (ppm)

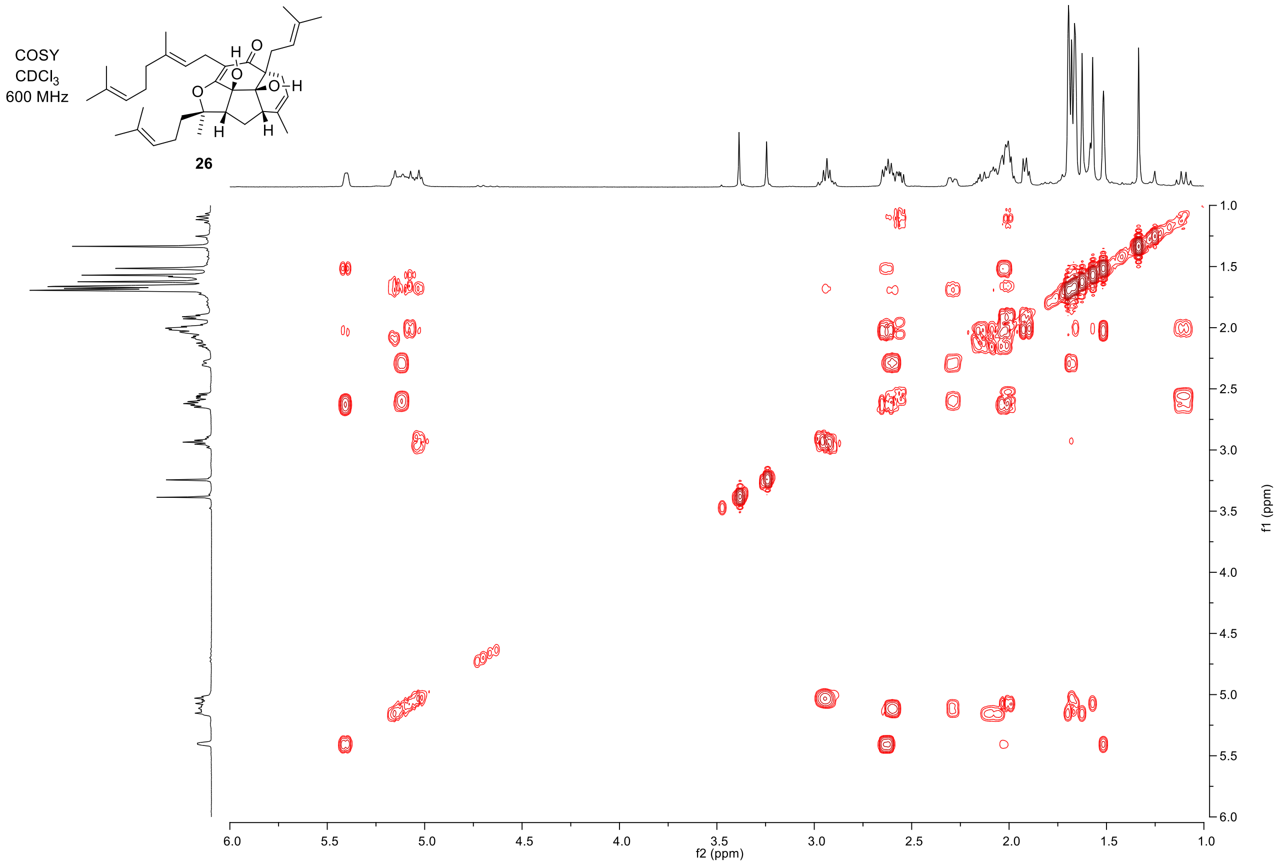
COSY
CDCl3
600 MHz
26
f2 (ppm)
f1 (ppm)

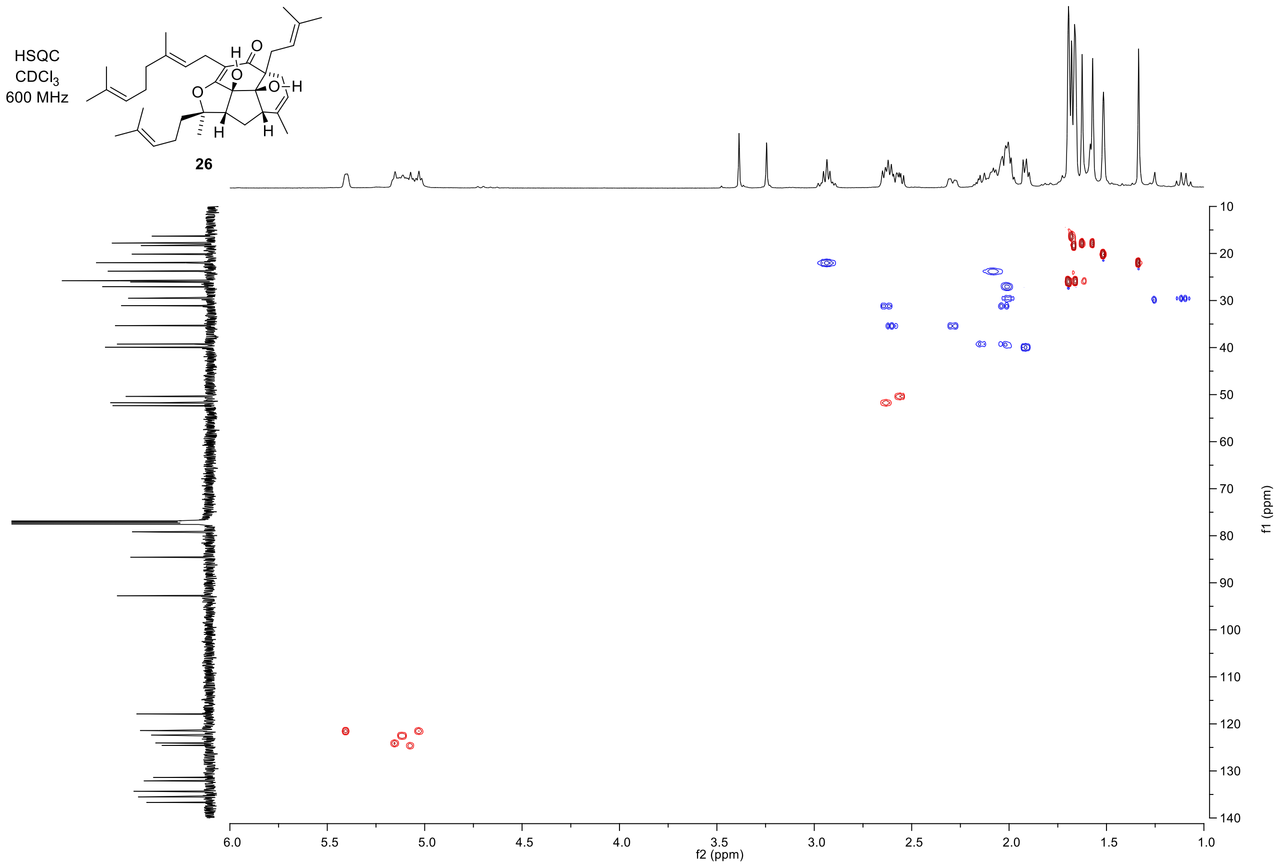
HSQC
CDCl3
600 MHz
26
f2 (ppm)
f1 (ppm)

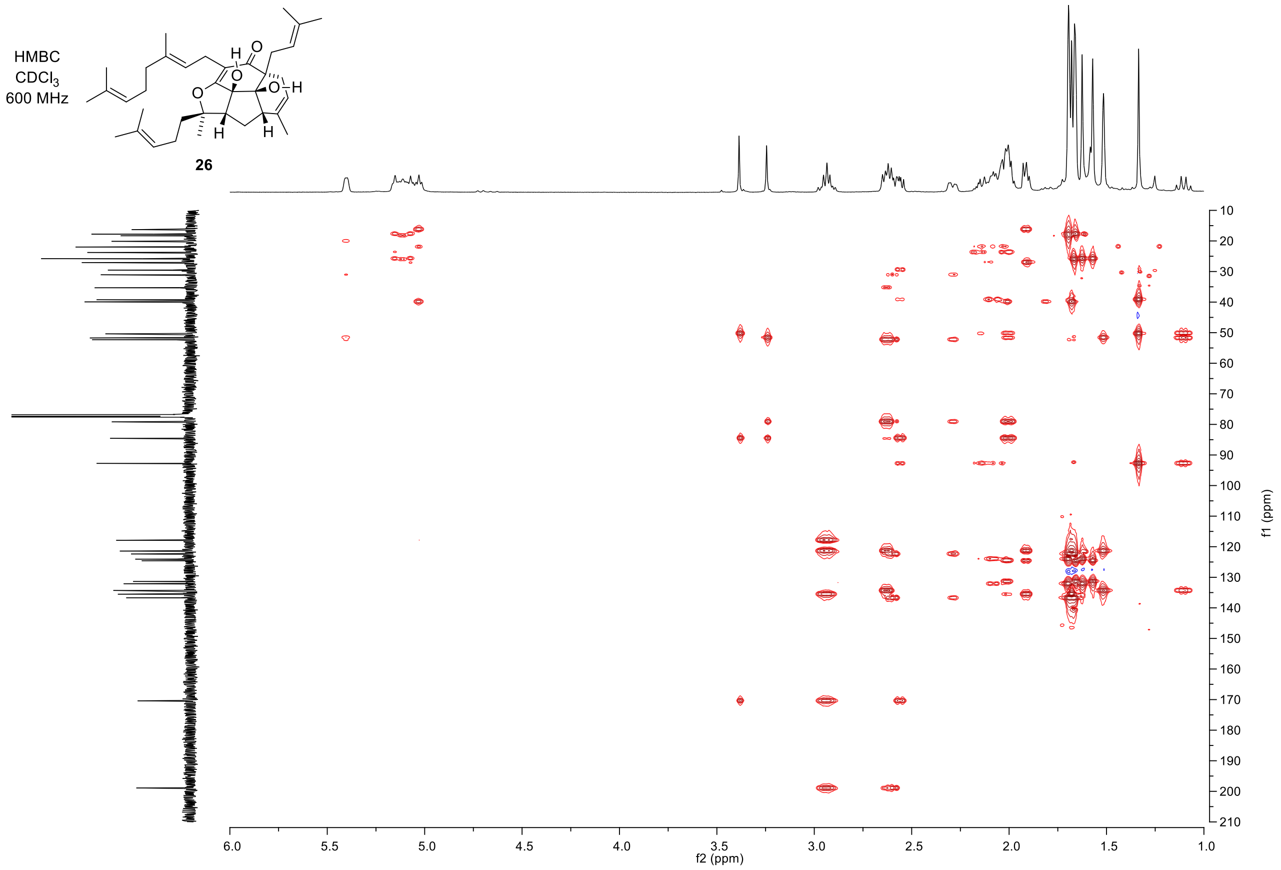
HMBC
CDCl$_3$
600 MHz
26
f2 (ppm)
f1 (ppm)

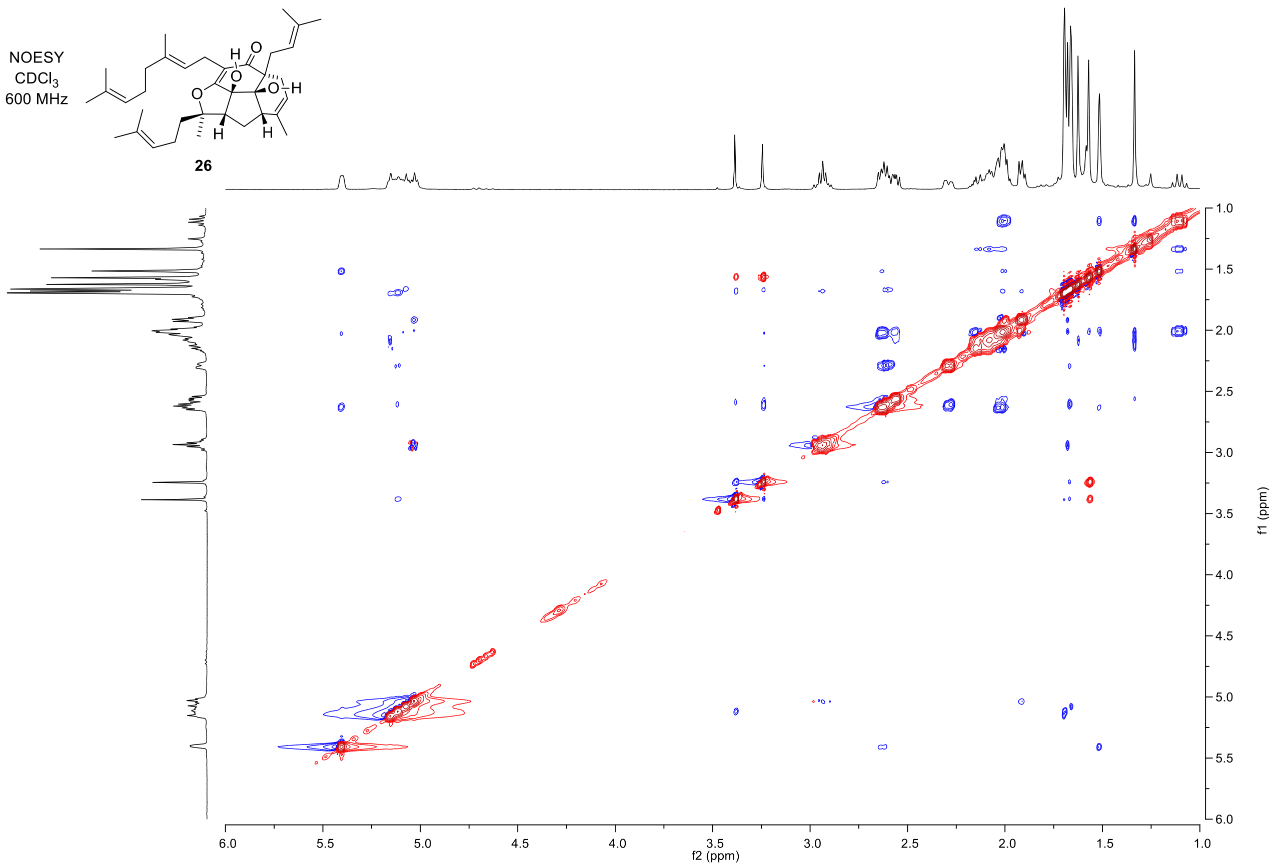
NOESY
CDCl$_3$
600 MHz
26
f2 (ppm)
f1 (ppm)

4. Tables of NMR Data

Table of ^1H and ^{13}C NMR spectroscopic data for natural[3] and synthetic erectquione A (**18**).

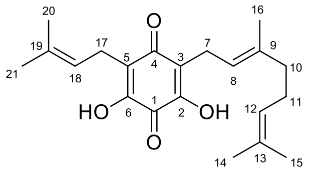

18: erectquione A

	^{13}C NMR ($CDCl_3$): δ_C ppm		^1H NMR ($CDCl_3$): δ_H ppm, mult. (J in Hz)	
Position	Natural **18**[a]	Synthetic **18**[b]	Natural **18**[a]	Synthetic **18**[b]
1	180.2	180.4		
2	147.9	148.2		
3	121.7	122.0		
4	186.7	186.9		
5	121.7	122.0		
6	147.9	148.2		
7	22.3	22.6	3.13, m	3.16, m
8	119.6	119.8	5.02, m	5.05, t (J = 6.6)
9	137.3	137.6		
10	39.6	39.9	1.94, m	1.96, m
11	26.5	26.7	2.01, m	2.04, m
12	124.1	124.3	5.08, m	5.12, t (J = 7.1)
13	131.3	131.6		
14	25.6	25.8[c]	1.67, s	1.65, s
15	17.6	17.8	1.59, s	1.57, s
16	16.1	16.3	1.51, s[d]	1.73, s
17	22.3	22.6	3.13, m	3.16, m
18	119.4	119.6	5.08, m	5.12, t (J = 7.1)
19	133.8	134.0		
20	25.6	25.9[c]	1.66, s	1.67, s
21	17.7	18.0	1.79, s	1.73, s
2-OH			6.47, s	6.42, s
6-OH			6.47, s	6.42, s

[a] ^{13}C and ^1H NMR spectroscopic data obtained at 100 MHz and 400 MHz, respectively.
[b] ^{13}C and ^1H NMR spectroscopic data obtained at 125 MHz and 500 MHz, respectively.
[c] Assignments interchangeable.
[d] This chemical shift reported by the isolation chemists is a suspected typographical error.

[3] T.-Y. An, M.-D. Shan, L.-H. Hu, S.-J. Liu, Z.-L. Chen, *Phytochemistry* **2002**, *59*, 395.

Table of ^{1}H and ^{13}C NMR spectroscopic data for natural[4] and synthetic erectone A (**2**).

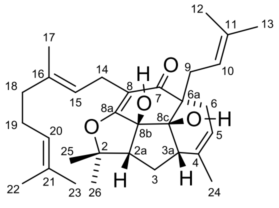

2: erectone A

	^{13}C NMR ($CDCl_3$): δ_C ppm		^{1}H NMR ($CDCl_3$): δ_H ppm, mult. (*J* in Hz)	
Position	Natural **2**[a]	Synthetic **2**[b]	Natural **2**[a,c]	Synthetic **2**[b]
2	90.2	90.3		
2a	52.0	52.1	2.45, dd (*J* = 11.3, 7.4)	2.48, dd (*J* = 11.3, 7.4)
3	29.4	29.5	β: 1.95, m	β: 2.03, m
			α: 1.07, m	α: 1.10, m
3a	51.6	51.7	2.56, m	2.63, m
4	134.5	134.4		
5	121.3	121.4	5.34, m	5.40, m
6	31.1	31.2	β: 2.53, m	β: 2.61, m
			α: 1.98, m	α: 2.03, m
6a	52.3	52.4		
7	198.9	199.0		
8	117.7	117.9		
8a	170.6	170.7		
8b	84.6	84.7		
8c	79.0	79.2		
9	35.2	35.4	2.53, m	2.61, m
			2.25, m	2.29, dd (*J* = 14.9, 5.5)
10	122.3	122.4	5.03, m	5.12, m
11	136.7	136.9		
12	25.9	26.1	1.58, s	1.66, s
13	18.2	18.3	1.63, s	1.70, s
14	21.8	22.0	2.87, m	2.93, m
15	121.2	121.3	5.01, t (*J* = 7.5)	5.07, t (*J* = 6.9)
16	135.4	135.6		
17	16.1	16.3	1.60, s	1.68, s
18	39.8	39.9	1.85, m	1.91, m
19	26.9	27.1	1.95, m	2.01, m
20	124.4	124.6	4.98, m	5.00, t (*J* = 6.9)
21	131.2	131.4		
22	25.6	25.8	1.60, s	1.68, s
23	17.0	17.8	1.50, s	1.57, s
24	19.9	20.1	1.45, s	1.51, s
25	27.2	27.3	1.60, s	1.68, s
26	25.0	25.1	1.30, s	1.36, s
8b-OH			3.37, br s	3.43, s
8c-OH			3.37, br s	3.21, s

[a] ^{13}C and ^{1}H NMR spectroscopic data obtained at 100 MHz and 400 MHz, respectively.

[b] ^{13}C and ^{1}H NMR spectroscopic data obtained at 150 MHz and 600 MHz, respectively.

[c] The isolation chemists appear to have referenced their ^{1}H NMR spectrum to $CHCl_3$ δ_H = 7.20 ppm; all ^{1}H NMR chemical shifts for synthetic **2** are approximately 0.06 ppm downfield of those reported for natural **2**.

[4] T.-Y. An, L.-H. Hu, Z.-L. Chen, K.-Y. Sim, *Tetrahedron Lett.* **2002**, *43*, 163.

Table of ^{1}H and ^{13}C NMR spectroscopic data for natural[4] and synthetic erectone B (**3**).

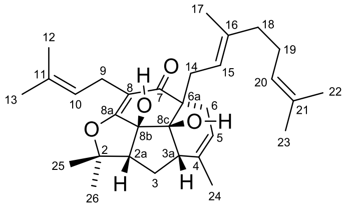

3: erectone B

	^{13}C NMR ($CDCl_3$): δ_C ppm		^{1}H NMR ($CDCl_3$): δ_H ppm, mult. (J in Hz)	
Position	Natural **3**[a]	Synthetic **3**[b]	Natural **3**[a,c]	Synthetic **3**[b]
2	90.3	90.3		
2a	52.0	52.1	2.41, dd (J = 11.3, 7.4)	2.47, dd (J = 11.2, 7.5)
3	29.4	29.4	β: 1.95, m	β: 2.02, m
			α: 1.07, m	α: 1.10, m
3a	51.6	51.7	2.57, m	2.63, m
4	134.4	134.5		
5	121.4	121.4	5.35, m	5.40, m
6	31.3	31.3	β: 2.54, m	2.63, m
			α: 1.95, m	2.02, m
6a	52.3	52.4		
7	199.1	199.1		
8	117.9	117.9		
8a	170.6	170.7		
8b	84.7	84.7		
8c	79.1	79.1		
9	22.0	22.0	2.85, m	2.91, m
10	121.6	121.7	5.02, m	5.04, m
11	131.7	131.8		
12	25.8	25.8	1.51, s[d]	1.67, s
13	17.9	18.0	1.60, s[d]	1.57, s
14	35.2	35.2	2.54, m	2.63, m
			2.24, m	2.30, dd (J = 14.6, 5.0)
15	122.2	122.2	5.05, m	5.14, dd (J = 9.0, 6.0)
16	140.3	140.3		
17	16.7	16.7	1.60, s	1.67, s
18	40.0	40.0	1.92, m	2.02, m
19	26.4	26.5	1.97, m	2.02, m
20	123.7	123.8	4.96, m	4.99, dd (J = 7.8, 6.8)
21	132.1	132.2		
22	25.7	25.7	1.55, s	1.61, s
23	17.5	17.8	1.60, s	1.67, s
24	20.0	20.0	1.45, s	1.51, s
25	27.2	27.3	1.60, s	1.67, s
26	25.0	25.1	1.30, s	1.36, s
8b-OH			3.36, br s	3.47, s
8c-OH			3.36, br s	3.26, s

[a] ^{13}C and ^{1}H NMR spectroscopic data obtained at 100 MHz and 400 MHz, respectively.

[b] ^{13}C and ^{1}H NMR spectroscopic data obtained at 125 MHz and 500 MHz, respectively.

[c] The isolation chemists appear to have referenced their ^{1}H NMR spectrum to $CHCl_3$ δ_H = 7.20 ppm; all ^{1}H NMR chemical shifts for synthetic **3** are approximately 0.06 ppm downfield of those reported for natural **3**.[4]

[d] The chemical shifts of C12 and C13 reported by the isolation chemists appear to be erroneously switched.

Table of ^{1}H and ^{13}C NMR spectroscopic data for natural[5] and synthetic hyperelodione D (**17**).

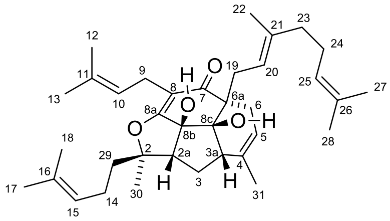

17: hyperelodione D
revised structure

	**^{13}C NMR ($CDCl_3$): δ_C ppm**		**^{1}H NMR ($CDCl_3$): δ_H ppm, mult. (*J* in Hz)**	
Position	Natural **17**[a]	Synthetic **17**[b]	Natural **17**[a]	Synthetic **17**[b]
2	92.8	92.8		
2a	50.3	50.3	2.56, dd (*J* = 11.4, 7.8)	2.56, dd (*J* = 11.3, 7.4)
3	29.4	29.4	α: 1.12, m	α: 1.12, m
			β: 2.01, m	β: 2.01, m
3a	51.7	51.7	2.64, m	2.64, m
4	134.4	134.4		
5	121.4	121.5	5.41, m	5.41, m
6	31.2	31.2	2.02, m	2.04, m
			2.64, m	2.64, m
6a	52.3	52.4		
7	199.1	199.1		
8	118.0	118.0		
8a	170.4	170.4		
8b	84.5	84.6		
8c	79.2	79.2		
9	22.0	22.0	2.92, m	2.92, m
10	121.7	121.8	5.03, m	5.04, m
11[c]	131.8	131.8		
12[d]	17.8	17.8	1.61, s	1.61, s
13[d]	25.8	25.8	1.62, s	1.62, s
14	23.8	23.8	2.07, m	2.08, m
15	124.0	124.1	5.13, m	5.14, m
16[c]	132.0	132.0		
17[d]	25.8	25.8	1.69, s	1.69, s
18[d]	18.0	17.9	1.67, s	1.67, s
19	35.3	35.3	2.29, dd (*J* = 14.4, 4.8)	2.29, dd (*J* = 15.1, 5.6)
			2.62, m	2.63, m
20	122.2	122.2	5.13, m	5.13, m
21	140.4	140.3		
22	16.8	16.8	1.67, s	1.67, s
23	40.0	40.0	1.98, m	1.98, m
24	26.5	26.5	2.02, m	2.02, m
25	123.8	123.8	5.02, m	5.02, m
26[c]	132.2	132.1		
27[d]	25.8	25.8	1.67, s	1.67, s
28[d]	17.8	17.8	1.57, s	1.57, s
29	39.2	39.3	2.14, m	2.05, m
				2.14, m
30	21.9	21.9	1.34, s	1.34, s

[5] D.-R. Qiu, M. Zhou, X.-Z. Liu, J.-J. Chen, G.-H. Wang, T. Lin, F.-R. Yu, R. Ding, C.-L. Sun, W.-J. Tian, H.-F. Chen, *Bioorganic Chemistry*, **2021**, *107*, 104578.

31	20.1	20.1	1.52, s	1.52, s
8b-OH			3.39, s	3.39, s
8c-OH			3.26, s	3.26, s

[a] ^{13}C and ^{1}H NMR spectroscopic data obtained at 150 MHz and 600 MHz, respectively.
[b] ^{13}C and ^{1}H NMR spectroscopic data obtained at 125 MHz and 500 MHz, respectively.
[c] Assignments interchangeable.
[d] Assignments interchangeable.

Comment on stereochemical assignment of tetracycles **1**, **2**, **3**, **15**, **17**, **20** and **26**:

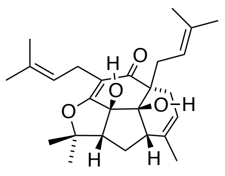

15

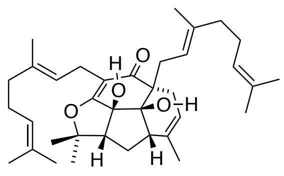

1

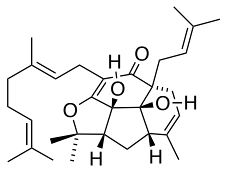

2: erectone A

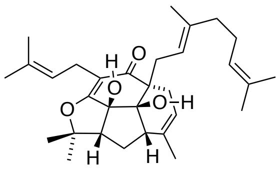

3: erectone B

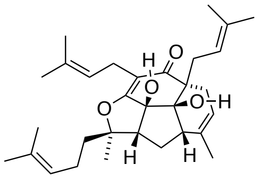

20

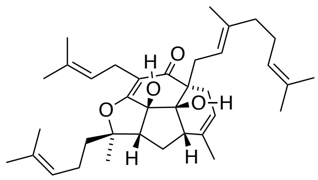

17: hyperelodione D
revised structure

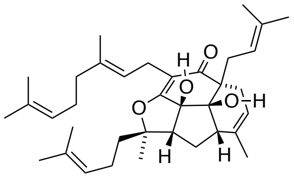

26

The structure of **15** was proven by X-ray crystallography. ^{1}H and ^{13}C NMR signals around the tetracyclic core **1**, **2**, and **3** are almost identical to **15**, *suggesting* that they have the same relative configuration.

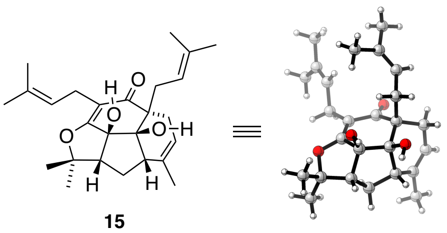

15

The key NOESY correlations (blue arrows) were observed for erectone A (**2**) and erectone B (**3**) to conclusively prove their relative configurations. Although most of these correlations were reported in the isolation paper, the resolution of the sharp 8b-OH and 8c-OH signals (which were originally reported as broad singlets) helped our analysis.

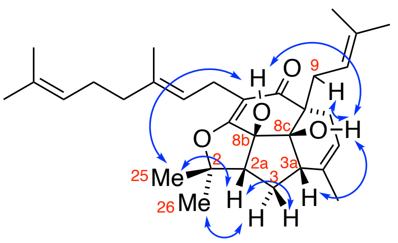

2: erectone A

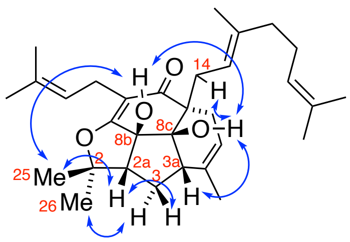

3: erectone B

The structures of **20**, **17** and **26** have an additional stereocentre at C2. This is clearly assigned as shown below by the observation of a strong NOESY correlation between Me-30 and the shielded H-3α in all three compounds. Some key correlations involving 8b-OH and 8c-OH were also visible.

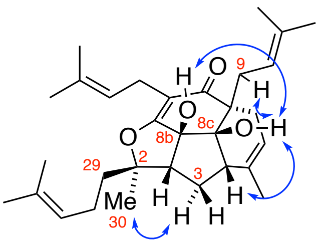

20

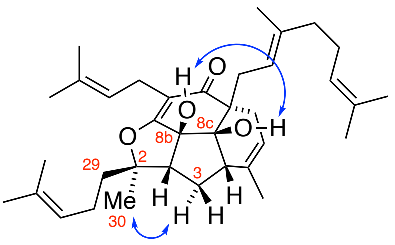

17: hyperelodione D
revised structure

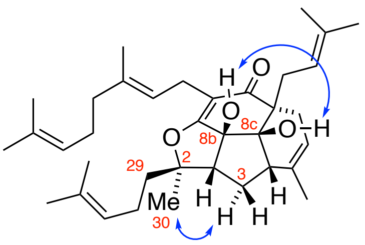

26

5. Single Crystal X-ray Data

A single crystal of **15** was mounted in Paratone-N oil on a MiTeGen micromount. X-ray diffraction data were collected at 150(2) K on an Oxford X-calibur single crystal diffractometer using Mo Kα radiation.[6] The data set was corrected for absorption using a multi-scan method, and the structure solved by direct methods (SHELXS)[7] and refined by full-matrix least squares on F2 by SHELXL,[8] interfaced through the programs X-Seed (version 4)[9] and Olex2.[10] All non-hydrogen atoms were refined anisotropically and hydrogen atoms were included as invariants at geometrically estimated positions.

Table S1 lists the X-ray experimental data and refinement parameters for the crystal structures. Perspective views of the structure of **15** is shown in Figure S1.

Full details of the structure determination have been deposited with the Cambridge Crystallographic Data Centre as CCDC 2116192 (**15**). Copies of this information may be obtained free of charge from The Director, CCDC, 12 Union Street, Cambridge CB2 1EZ, U.K. (fax, +44-1223-336-033; e-mail, deposit@ccdc.cam.ac.uk).

Table of X-ray experimental data for **15**.

Compound	**15**
CCDC number	2116192
cif	**1LF43**
Empirical formula	$C_{26}H_{36}O_4$
Formula weight	412.55
Crystal system	monoclinic
Space group	$P2_1/n$
a (Å)	12.5645(3)
b (Å)	14.1787(3)
c (Å)	12.7707(3)
α (°)	90
β (°)	101.429(2)
γ (°)	90
Volume (Å^3)	2229.97(9)
Z	4
Density (calc.) (mg/m^3)	1.229
Absorption coefficient (mm^{-1})	0.081
F(000)	896.0
Crystal size (mm^3)	0.75 × 0.51 × 0.24
2θ range for data collection (º)	7.094 to 58.816
Reflections collected	38834
Observed reflections [$R(int)$]	5629 [R_{int} = 0.0459]
Data/restraints/parameters	5629/0/280
Goodness-of-fit on F2	1.030
R_1 [I>2σ(I)]	0.0454
wR_2 (all data)	0.1137
Largest diff. peak and hole (e.Å^{-3})	0.38/-0.20

[6] CrysAlisPro 1.171.38.43d (Rigaku Oxford Diffraction, **2015**)

[7] (a) G. M. Sheldrick, *Acta Cryst.*, **2008**, A64, 112-122. (b) G. M. Sheldrick, SHELXT - Integrated space-group and crystal-structure determination. *Acta Cryst.*, **2015**, A71, 3-8.

[8] G. M. Sheldrick, *Acta. Cryst.*, **2015**, C71, 3-8.

[9] L. J. Barbour, *J. Appl. Cryst.*, **2020**, 53, 1141-1146.

[10] O. V. Dolomanov, L. J. Bourhis, R. J. Gildea, J. A. K. Howard, H. Puschmann, *J. Appl. Cryst.*, **2009**, 42, 339-341.

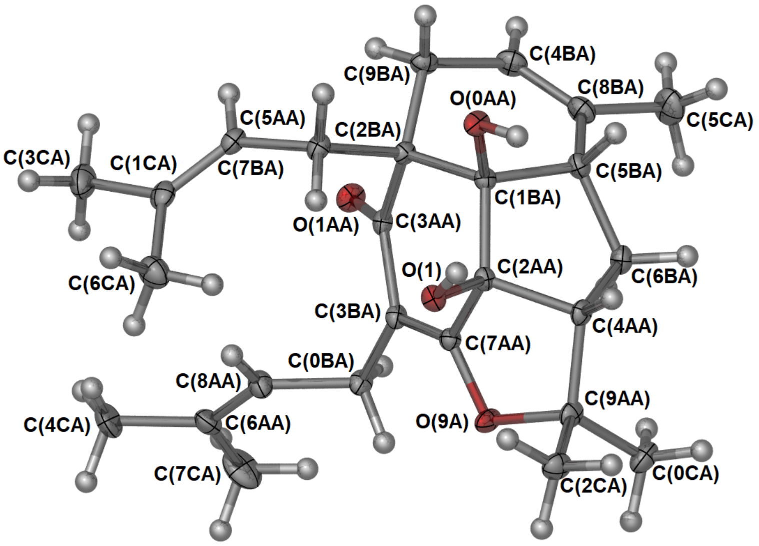

(a)

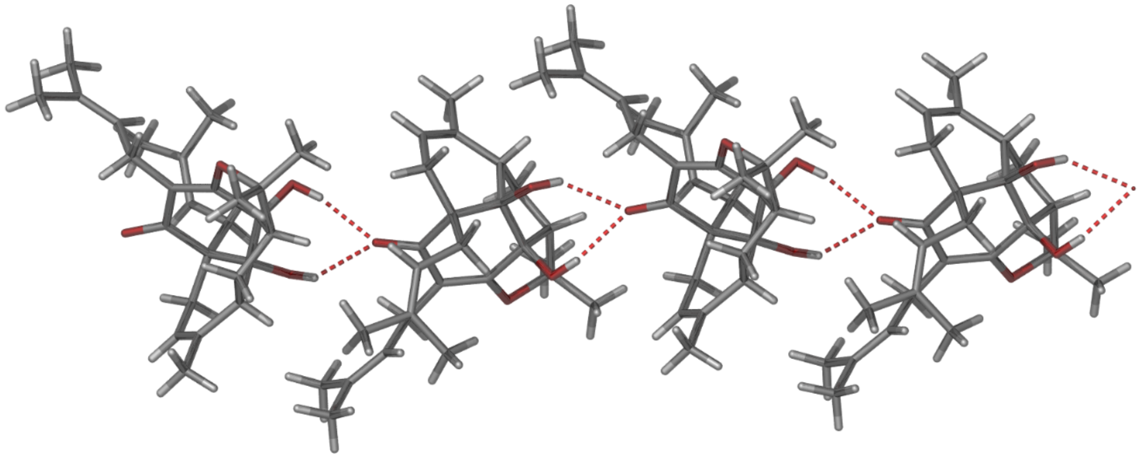

(b)

Figure S1. Perspective views of (a) the labelled asymmetric unit and (b) the hydrogen bonded chains, which are directed along the crystallographic *b* axis (2_1 screw axis), in the structure of **15**. Carbon – grey, hydrogen – white, oxygen – red. The intermolecular hydrogen bonding parameters, which connect the base of one bowl-shaped molecule of **15** to the top of the next, are: $D_{O1\text{-}H1\cdots O1AA}$ = 2.83 Å, $angle_{O1\text{-}H1\cdots O1AA}$ = 143.3° and $D_{O0AA\text{-}H0AA\cdots O1AA}$ = 2.85 Å, $angle_{O0AA\text{-}H0AA\cdots O1AA}$ = 163.2°, respectively.
